# Neural heterogeneity enables adaptive encoding of time sequences

**DOI:** 10.1038/s42005-025-02408-3

**Published:** 2025-11-24

**Authors:** Raphaël Lafond-Mercier, Leonard Maler, Avner Wallach, André Longtin

**Affiliations:** 1 Department of Physics, University of Ottawa, Ottawa, ON Canada; 2 Department of Cellular and Molecular Medicine, University of Ottawa, Ottawa, ON Canada; 3 Brain and Mind Institute, University of Ottawa, Ottawa, ON Canada; 4 Center for Neural Dynamics and Artificial Intelligence, University of Ottawa, Ottawa, ON Canada; 5 Faculty of Biology, Technion – Israel Institute of Technology, Haifa, Israel

**Keywords:** Computational biophysics, Applied mathematics

## Abstract

The timing mechanisms in biological systems operate across a vast range of scales, from microsecond precision for sound localization to annual cycles. A key open question concerns the mechanisms for encoding intermediate time intervals–hundreds of milliseconds to minutes–that are essential for navigation, communication, memory, and prediction. Here we present a theoretical framework that explains how neurons can represent such intervals using a common biophysical property: neural fatigue, where activity diminishes during sustained stimulation. Our Bayesian framework combines parametrically heterogeneous stochastic dynamical modeling with interval priors to predict available timing information independent of the actual decoding mechanism. We find that a trade-off emerges between accurately representing the most recent interval and retaining information about previous ones. We show that cellular diversity is not just tolerated but required to encode sequences of time intervals. Our work highlights the computational role of biological heterogeneity in shaping memory for time, with implications for understanding temporal processing in neural circuits.

## Abstract (web summary)

Biological systems must encode time intervals crucial for tasks like navigation and communication, yet mechanisms for intervals of hundreds of milliseconds to minutes remain unclear. The authors develop a Bayesian framework using neural fatigue and cellular heterogeneity to optimize interval representation, enhancing our understanding of timing memory and its computational roles.

## Introduction

Long before human-made tools^1^, biological systems devised multiple molecular and electric mechanisms to keep time over a wide range of scales^2^. Much is known about the interaction of neural activity and molecular clocks that generates hours to days-long circadian rhythms^3^. Other time scales include those of hundreds of milliseconds to a second, which, together with neural plasticity, are important for speech^4^, song generation in birds^5^, and motor control^6^. Shorter still, merely tens of microseconds are necessary for the auditory circuits in barn owls to pinpoint the direction of a sound^7^.

The intermediate scale of seconds to minutes is relevant, e.g. to memorizing and recalling a path in space, a conversation or executing sequences of movements^8–10^. Much effort is devoted to uncovering how the temporal structure of experience on these time scales, i.e., episodic memory, is stored for later recall and predictions of the future^11,12^. In mammals, spatiotemporal information is available in the hippocampus, mainly area CA1, where time cells fire at given points in time, complementing place cells that fire when the animal is at given points in space^13^. Ongoing work seeks to explain how this information is integrated to produce sequences of time and place-specific activations of neural ensembles over hours and days^14,15^. Time cells can, in principle, represent intervals between contiguous events^13,16,17^, though the associated encoding and decoding mechanisms are not well understood. There is also evidence that the representation of time duration starts before the hippocampus in the sensory cortex^18^.

A different paradigm of time coding has recently been identified in a thalamic structure in weakly electric fish known as the preglomerular complex (PG). It projects to the dorso-lateral pallium^19^, a likely homolog of the mammalian hippocampus^20^, which is highly recurrent^21^. PG cells use a counterintuitive, starkly different algorithm and biophysical substrate, offering a window into time coding in mammals, as they have similar structures. PG cells receive electrosensory information about their murky nocturnal environment from the optic tectum^22^. The electrosense has much in common with vision^23^: the electroreceptors in the skin read out perturbations to the fish’s self-generated electric field caused by objects in the environment^24^. Whenever the fish encounters an object, many PG cells produce brief bursts of firing, regardless of which body part came closest to the object. The number of spikes in a burst depends on the time since the last encounter, and thus the sequence of bursts can be mapped to the sequence of intervals between encounters^19^. This unconventional mechanism relies on deterministic and stochastic dynamics.

Here, we develop its general theory, addressing how it can optimally represent the distribution of time intervals through the heterogeneity of parameters across PG cells. Two-thirds of adaptive PG cells are sensitive to the history of recent encounters and, accordingly, have recovery time constants of tens of seconds. The other third can encode only the last interval. Why are both present? This question relates to the fundamental problem of optimal information processing in the face of available biophysical diversity. Heterogeneity has attracted much attention recently as an essential part of coding schemes^25–28^. We show that the observed diversity among adaptive cells reflects a division of labor in encoding temporal information. Indeed, using a Bayesian formulation and Fisher information analysis, we first determine that the optimal parameter regime for encoding a single interval is a unique time constant with no memory of previous intervals. This is then generalized by considering a continuous prior distribution of intervals and of time constants, at the same time showing that single interval performance is robust to the heterogeneity of time constants. This is convenient because we then find that heterogeneity is mathematically necessary to encode sequences of multiple intervals through the analysis of the invertibility of the set of resulting equations. This is further validated with machine learning-based decoders, showing that their performance is significantly enhanced by heterogeneous adaptation. Our work therefore presents a fundamental computational role for heterogeneity to represent sequences of time intervals.

## Results

**Fig. 1:**
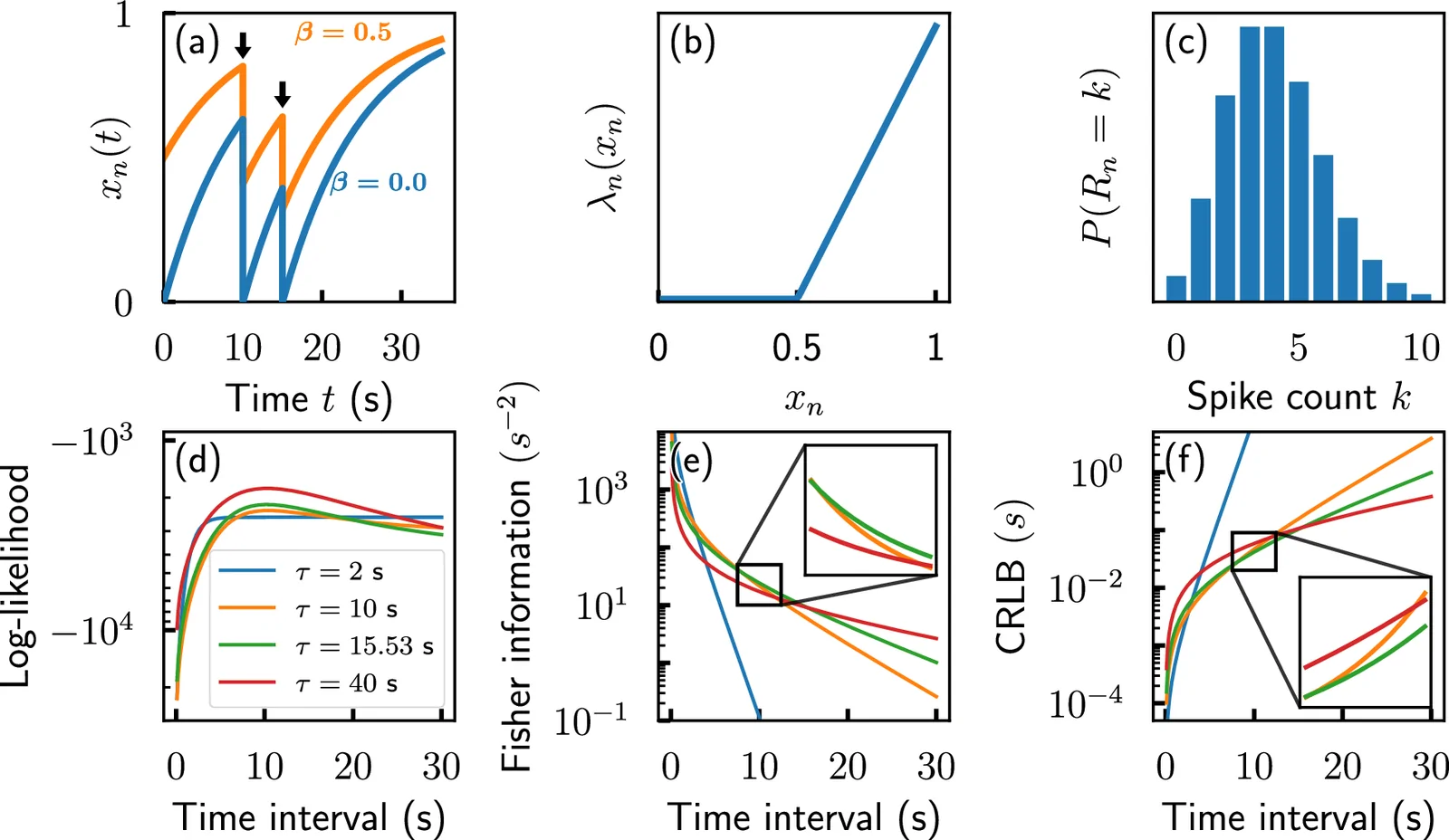
Generating the adaptation responses, estimating time interval and quantifying the performance of maximum likelihood estimation. **a** The resource variable *x* recovers with time constant *τ* and the remaining fraction after an encounter (black arrows) is proportional to the memory parameter *β* as defined by Equation (1). **b** During each encounter, a firing parameter *λ* is computed from the resource variable *x* through a rectified linear transformation with gain parameter *a* and baseline activity *c* as defined by Equation (2). **c** A Poisson distribution with parameter *λ* dictates the number of spikes generated during an encounter as defined by Equation (3). **d** Example of the log-likelihood as defined by Equation (6) for a real time interval of 10 s between encounters, computed with the responses of 1000 identical model PG cells during the last encounter. This is shown for four recovery time constants. The log-likelihood has a maximum around 10 s for most populations. **e** Fisher information (FI) as defined by Equation (8) of 1000 PG cells. **f** Cramér-Rao Lower Bound (CRLB) as defined by Equation (7) of the maximum likelihood estimator of 1000 identical PG cells. **e** and **f** The FI (CRLB) decreases (increases) as *T* increases, and a value of *τ* gives larger (smaller) FI (CRLB) than another one only in a specific range of values of *T*. **d**–**f** The legend is shared among the panels and the parameters are given by *a* = 10, *c* = 0 and *β* = 0.

We considered a network of independent cells receiving brief input stimulation in parallel at times ${t}_{k}={\sum }_{i=1}^{k}{T}_{i}$, where *T*_*i*_ is the time interval between encounters happening at times *t*_*i*−1_ and *t*_*i*_, with *t*_0_ = 0 the time of the first stimulus. We modeled the dynamical adaptation of PG cells using a simple resource-based model^19,29^ with long replenishment time constants. A cell has an amount of resources available to produce spikes during an encounter with an object, which is represented by the variable *x* ∈ [0, 1]. During an encounter, the resource variable is depleted by an amount that depends on the memory parameter *β* ∈ [0, 1], and then recovers at a rate *τ* as shown in Fig. 1a and defined by Eq. (1). The value of this resource variable during an encounter is then passed through a linear rectification with gain *a* and baseline activity *c* to generate a firing parameter *λ* as shown in Fig. 1b and defined by Equation (2). Finally, a Poisson probability distribution with parameter *λ* represents the probability of having a given spiking response during the encounter, as shown in Fig. 1c and defined by Eq. (3). We then use samples from this Poisson distribution to simulate the number of spikes *R*_*n*_ that would be observed in PG cells during an encounter *n*. Since this is an event-based version of an inhomogeneous Poisson process, changing *a* could be seen as changing the observation time window. However, we assume that this window is identical for all cells and, therefore, that the value of *a* corresponds to the sensitivity to available resources. Moreover, *c* could be considered as noise if it is strictly positive. This is usually the case, but not always (see Discussion), which is why we do not call it noise. The process is summarized in the following equations:

1 $${x}_{n}\left({\{{T}_{i}\}}_{i = 1}^{n}\right)=1-{e}^{-{T}_{n}/\tau }(1-\beta {x}_{n-1}),$$

2 $${\lambda }_{n}\left({\{{T}_{i}\}}_{i = 1}^{n}\right)={\left[a{x}_{n}+c\right]}_{+},$$

3 $$P\left({R}_{n}\right)=\frac{{({\lambda }_{n})}^{{R}_{n}}{e}^{-{\lambda }_{n}}}{{R}_{n}!}.$$

It is important to note that the response value *R*_*n*_ of one cell does not directly influence future responses *R*_*n*+1_, *R*_*n*+2_, . . . Specifically, they are conditionally uncorrelated given a choice of successive intervals, but become dependent upon marginalizing over the prior of intervals through the memory in the *x*-process. Indeed, we assume that the resource-based dynamics is a result of pre-synaptic activity, although we can not exclude contributions from intrinsic currents in PG cells. It is also possible that other nuclei downstream from PG – such as the pallium – further influence adaptation time scales through e.g. feedback loops^30^. Therefore, the statistics of all PG neurons dictate future responses, which is what is modeled here.

These neurons project downstream to a decoder that extracts the sequence ${\{{T}_{i}\}}_{i = 1}^{n}$ for further use such as storage, not modeled here. We approached this problem using a Bayesian framework combined with signal detection theory and simple neuron stochastic dynamics with or without memory. Neuron output spikes thus result from a stochastic spike-generating process, with time-dependent parameter set by single-neuron Markovian deterministic dynamics that are driven by external inputs, namely, the sequence of intervals between encounters. Recovering the sequence of time intervals from the responses of PG cells *during the last encounter* can be achieved through maximum likelihood estimation (MLE), which is an efficient estimator given enough cells. In other words, MLE is the estimator yielding the lowest possible error among all possible unbiased estimators, making it a baseline for an idealized context.

From Bayes’ rule, we can write the probability that some sequence of time intervals ${\{{T}_{i}\}}_{i = 1}^{n}$ generated a set of *N* responses ${\{{R}_{n}^{j}\}}_{j = 1}^{N}$ during the *n*-th encounter as

4 $$P\left({\{{T}_{i}\}}_{i = 1}^{n}\big| {\{{R}_{n}^{j}\}}_{j = 1}^{N}\right)=\frac{P\left({\{{R}_{n}^{j}\}}_{j = 1}^{N}\big| {\{{T}_{i}\}}_{i = 1}^{n}\right)P\left({\{{T}_{i}\}}_{i = 1}^{n}\right)}{P\left({\{{R}_{n}^{j}\}}_{j = 1}^{N}\right)}.$$

When treating multiple neurons or populations, the superscript will be the index of an adaptive neuron or population, while the subscript will be the index of an encounter. Parentheses will be used to differentiate indices from exponents when necessary. However, to lighten up the notation, expressions with only one set of neuron parameters (i.e. single homogeneous population) will not have this differentiation; superscripts will be considered as exponents.

Finding the set of intervals ${\{{T}_{i}\}}_{i = 1}^{n}$ that maximizes Equation (4) yields the maximum a posteriori estimator. This estimator makes use of prior information on the sequence of time intervals through $P\left({\{{T}_{i}\}}_{i = 1}^{n}\right)$. However, since we want to quantify how much of the sequence of time intervals is encoded solely in the response of PG neurons, we assume for now that no prior information is available for the estimation. Maximizing Equation (4) then becomes equivalent to maximizing the likelihood

5 $$L\left({\{{T}_{i}\}}_{i = 1}^{n}\big| {\{{R}_{n}^{j}\}}_{j = 1}^{N}\right)=P\left({\{{R}_{n}^{j}\}}_{j = 1}^{N}\big| {\{{T}_{i}\}}_{i = 1}^{n}\right)$$

or maximizing the log-likelihood (LL)

6 $$\ell \left({\{{T}_{i}\}}_{i = 1}^{n}\big| {\{{R}_{n}^{j}\}}_{j = 1}^{N}\right)={\sum }_{j=1}^{N}\left({R}_{n}^{j}\ln {\lambda }_{n}^{j}\left({\{{T}_{i}\}}_{i = 1}^{n}\right)-{\lambda }_{n}^{j}\left({\{{T}_{i}\}}_{i = 1}^{n}\right)-\ln {R}_{n}^{j}!\right).$$

An example of LL computation for a single time interval is shown in Fig. 1d.

### Cell parameters can be optimized through Fisher information

We first assessed how the parameters of the network affect the time interval estimation. To do so, we used the Fisher information (FI), which is a measure of the maximum precision of an unbiased estimator. Indeed, it is used to compute a lower bound of the root mean squared error (RMSE) made by the estimator called the Cramér-Rao lower bound (CRLB) as defined by Equation (7).

Since the responses of PG neurons during an encounter are assumed to be independent Poisson variables, we can add the FI of the individual neurons^31^ to get the total FI of a network of PG cells as defined by Eq. (8). The resulting expression depends on the values of the time intervals (*T*_1_, *T*_2_, . . . , *T*_*n*_) in the stimulus sequence as shown in Fig. 1e, f. It also depends on the different gain (*a*^*k*^), baseline activity (*c*^*k*^), memory (*β*^*k*^) and recovery time (*τ*^*k*^) through the firing parameter ${\lambda }_{n}^{k}$:

7 $${\left[{{{\rm{CRLB}}}}\right]}_{ij}=\sqrt{{\left[{{{{\mathcal{I}}}}}^{-1}\right]}_{ij}}\le {\left[{{{\rm{RMSE}}}}\right]}_{ij},$$

where

8 $${\left[{{{\mathcal{I}}}}\right]}_{ij}=\sum_{k=1}^{N}\frac{1}{{\lambda }_{n}^{k}}\left(\frac{\partial {\lambda }_{n}^{k}}{\partial {T}_{i}}\right)\left(\frac{\partial {\lambda }_{n}^{k}}{\partial {T}_{j}}\right).$$

**Fig. 2:**
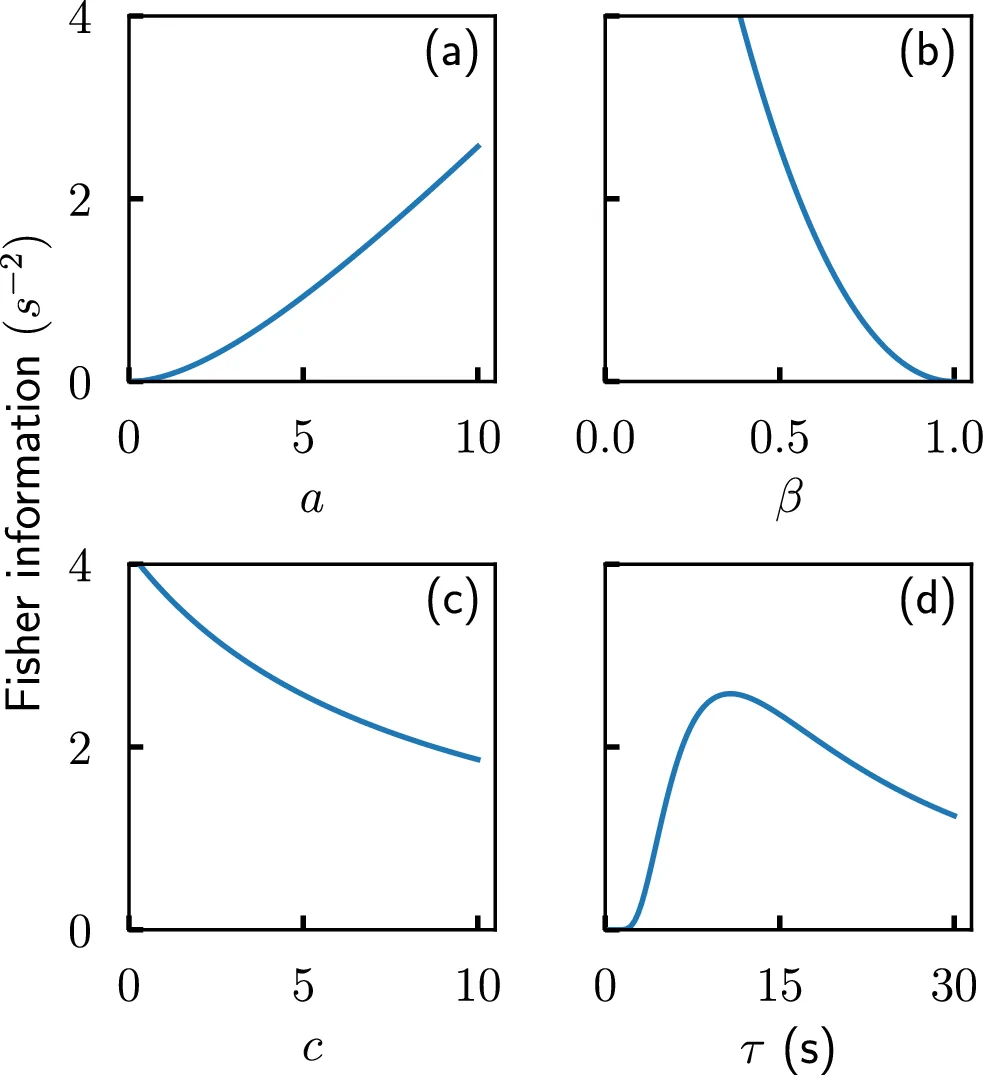
Maximizing the Fisher information for a single time interval in the homogeneous case. Example of the Fisher information (FI) as defined by Equation (8) for *N* = 1000 cells and an interval of 10 s as a function of the **a** gain *a*, **b** memory *β*, **c** baseline activity *c* and **d** recovery time *τ*. Maximum values of FI are reached at either end of the parameter domain with the exception of *τ*. Unless otherwise stated, the values of the parameters are *a* = 10, *c* = 5, *τ* = 10 s, *β* = 0.5 and *x*_0_ = 1. Note that the maximum of *τ* in **d** would be at 15.5 s if *β* and *c* were zero. The y-axis is shared by all panels.

To achieve a comprehensive understanding of this dynamical system, we began by focusing on the simplest case of a single time interval *T* between two consecutive encounters. We neglected previous encounters by setting the initial state of the neurons *x*_0_ = 1, which they eventually reach when there has not been an encounter for a significant time. Optimizing the FI for this specific case is simple and can be done by looking at the partial derivatives of the FI with respect to the different cell parameters (see subsection “Maximizing the Fisher information for a specific time interval" in Methods). We showed that, for any value of the time interval *T*, the partial derivative with respect to the gain parameter *a* is always positive, which means that a gain parameter as large as possible is preferable. Similarly, the derivative with respect to the memory parameter *β* and the baseline activity *c* is always negative; an indication that having no memory (*β* = 0) and no spontaneous activity (*c* = 0) optimally encodes a single time interval. This is represented in Fig. 2a–c.

The situation is different for the derivative with respect to *τ*, where a single value of *τ* maximizes the FI for a specific choice of *T* as shown in Fig. 2d. We numerically computed with Newton’s root finding algorithm that *τ* ≈ 1.5533*T* optimizes the FI when there is no baseline or memory (*β* = *c* = 0). However, the network should not be optimized to estimate a singular time interval value. Rather, it should be able to optimally estimate the wide range of interval values found in nature. To do so, we looked at the expectation value of the CRLB for a given prior of time intervals. For a single population of *N* cells, gain *a*, time constant *τ* and without baseline activity or memory (*c* = *β* = 0), we can explicitly write

9 $${E}_{T}\left[{{{\rm{CRLB}}}}\right]=\frac{{\tau }^{2}}{aN}\left\{{M}_{T}\left(\frac{2}{\tau }\right)-{M}_{T}\left(\frac{1}{\tau }\right)\right\},$$

where *E*_*T*_[ ⋅ ] is the expectation value with respect to *T* and ${M}_{T}(x)={E}_{T}\left[{e}^{xT}\right]$ is the moment-generating function dependent on the chosen prior distribution of time intervals. It is then possible to find the value of *τ* that minimizes this average CRLB. For example, an exponential prior distribution of time intervals with an average of $\hat{T}$ yields an optimal time constant of $\tau =(3+\sqrt{3})\hat{T}\approx 4.73\hat{T}$, while a uniform distribution between 0 and ${T}_{\max }$ yields an optimal time constant of $\tau \approx 1.14{T}_{\max }$ (see Supplementary Note 1). Moreover, we showed that for any prior distribution of time intervals, there can be at most one value of *τ* which minimizes the average CRLB (see Supplementary Note 2). However, it is not clear what the effect of having more populations of cells with different time constants is on the error. This therefore raised the question of what distribution of time constants is optimal given a time interval prior distribution.

### Heterogeneity of time constants has a small negative impact on single interval estimates

**Fig. 3:**
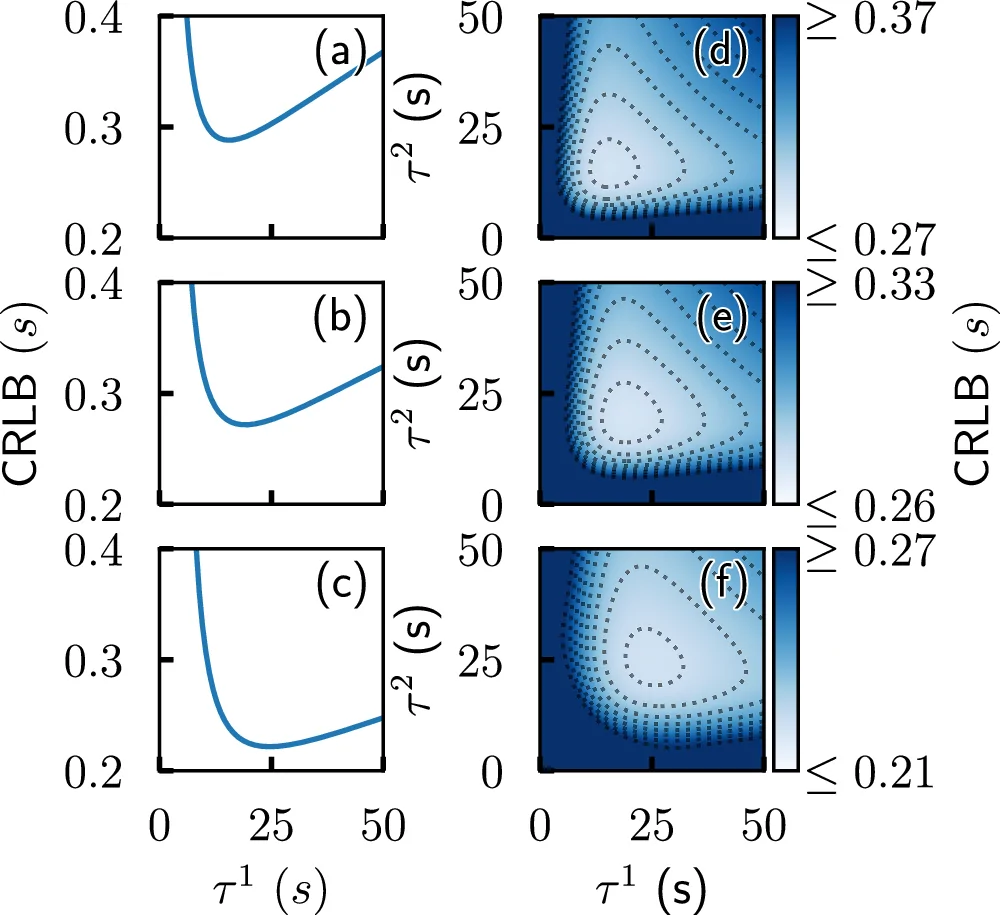
Six cases show how adding new sub-populations affects the error made on single time interval estimates. **a**–**c** Single population with only one recovery time *τ*^1^, gain *a*^1^ = 5, baseline activity *c*^1^ = 0 and memory *β*^1^ = 0. **a** Cramér-Rao lower bound (CRLB) for a time interval of *T* = 10 s. **b** Average of the CRLB as defined by Equation (25) for time intervals of *T* = 10 s and *T* = 15 s. The minimum shifts slightly towards a larger value of *τ*^1^ when compared to **a**. **c** Average of the CRLB for time intervals of *T* = 2 s and *T* = 20 s. The minimum remains unique even with a large difference between time interval values. **d**–**f** Two populations with recovery times *τ*^1^ and *τ*^2^, gains *a*^1^ = 5 and *a*^2^ = 15, baseline activities *c*^1^ = *c*^2^ = 0 and memory *β*^1^ = *β*^2^ = 0. **d** CRLB for a time interval of *T* = 10 s. **e** Average of the CRLB for time intervals of *T* = 10 s and *T* = 15 s. Similarly to **b**, the minimum shifts slightly towards larger values of *τ*^1^ and *τ*^2^. **f** Average of the CRLB for time intervals of *T* = 2 s and *T* = 20 s. The minimum remains unique even with a large difference between time interval values. For **d**–**f**: dashed lines represent contours with the same value of CRLB, and the minimum is on the diagonal *τ*^1^ = *τ*^2^ with the same value as in **a** to **c**, respectively. The x-axis is shared by all panels while the y-axis is shared column-wise.

To determine whether combining different values of *τ* over different cells is beneficial in the estimation of the time interval values of interest, we started by building our intuition using six simplified cases. For all six cases, we chose fixed parameters for *a*, *c* and *β* while looking at the effect of *τ*. The first two cases looked at the CRLB of a network of 1000 cells with a unique value of *τ*^1^ and *a*^1^ = 5. At a specific value of time interval *T* = 10 s, there is a unique maximum at *τ*^1^ ≈ 15.5*T*, which is expected because of the relationship between *τ* and *T* previously mentioned. This is shown in Fig. 3a. We then averaged the CRLB over two distinct values of time intervals *T* = 10 s and *T* = 15 s using Equation (25). We found that no new maximum appears and that the optimal value of *τ*^1^ is simply shifted as shown in Fig. 3b.

The same computations were repeated after adding a second, possibly different value of *τ*. In other words, a network is composed of a sub-population of 500 cells with *τ*^1^ and *a*^1^ = 5 and another sub-population of 500 cells with *τ*^2^ and *a*^2^ = 15. In the case where the CRLB for a single time interval *T* = 10 s is computed, the optimal combination of *τ*^1^ and *τ*^2^ is unique, namely, *τ*^1^ = *τ*^2^ ≈ 15.5*T* as shown in Fig. 3d. This is also the case when averaging the CRLB over two distinct time intervals, where the optimal value of *τ*^1^ = *τ*^2^ is shifted as shown in Fig. 3e. To make sure this is not simply due to the similarity between both intervals, we repeated the same computation with *T* = 2 s and *T* = 20 s as shown in Fig. 3c, f. The observations are the same, even with this large difference in time interval values. These simple cases suggested that a homogeneous distribution of *τ* may actually be optimal if we want to maximize the average performance of the MLE for a single time interval.

Repeating the same exercise for the relative CRLB by dividing the error by the time interval value, the results are similar with the exception of new maxima being added when shorter time intervals are considered. The old maximum becomes a saddle point when a new value of *τ* is added (see Supplementary Fig. 1f).

**Fig. 4:**
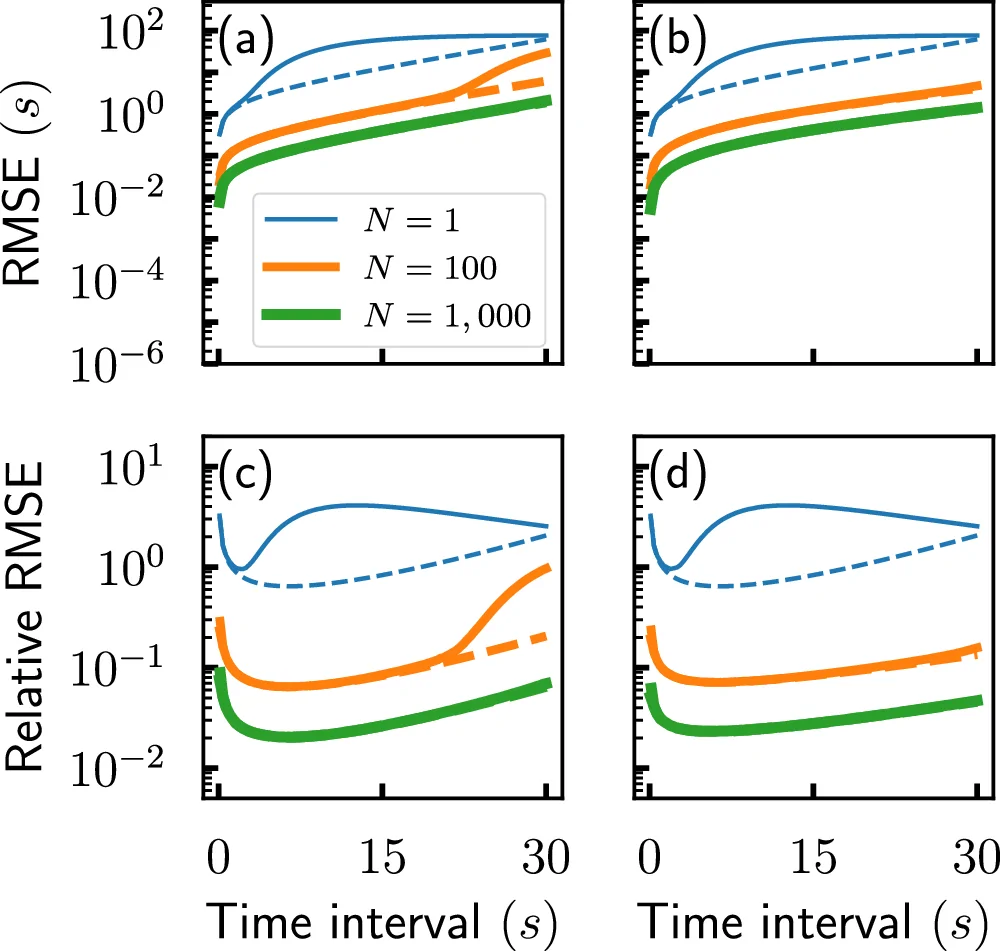
The error tends towards the Cramér-Rao lower bound for a single interval. Monte Carlo computation of the root mean square error (RMSE, solid lines) as defined by Equation (23) and Cramér-Rao lower bound (CRLB, dashed lines) as defined by Equation (26) of networks with increasing cell counts as a function of the value of the time interval between two encounters. **a** Homogeneous network with recovery time *τ* = 10 s for all cells. There is a significant bias causing the RMSE to diverge from the CRLB when the cell count is low. **b** Heterogeneous network with *τ* sampled from a uniform distribution between 0.1 s and 20 s. **c** and **d** Same as **a** and **b**, respectively, but the relative error RMSE/*T* is shown. There is also a bias for the heterogeneous network, but it is reduced more rapidly as the number of cells is increased than in the homogeneous case. For both networks, *a* = 10, *c* = 0 and *β* = 0. The x-axis is shared by all panels, while the y-axis is shared row-wise. The legend is shared by all panels.

To assess the validity of this result, we compared the actual error made with MLE with the CRLB. In the case of one-time interval, the RMSE computed through Monte Carlo simulations tends towards the CRLB as shown in Fig. 4a, b. For a network of 1000 cells, the bias is practically non-existent, and the RMSE becomes close to the CRLB. This justifies the use of FI for the parameter optimization of the MLE. There is a difference in the behavior of the RMSE between a homogeneous network as in Fig. 4a, and a random heterogeneous network as in Fig. 4b. The error made due to the bias decreases faster as *N* increases in the heterogeneous case than in the homogeneous one. *This suggests that heterogeneity helps in a situation where bias is important*. However, since PG contains approximately 60,000 cells^21^ – which suggests bias is small – we decided not to explore this effect further. The same happens when considering the relative RMSE as shown in Fig. 4c, d.

**Fig. 5:**
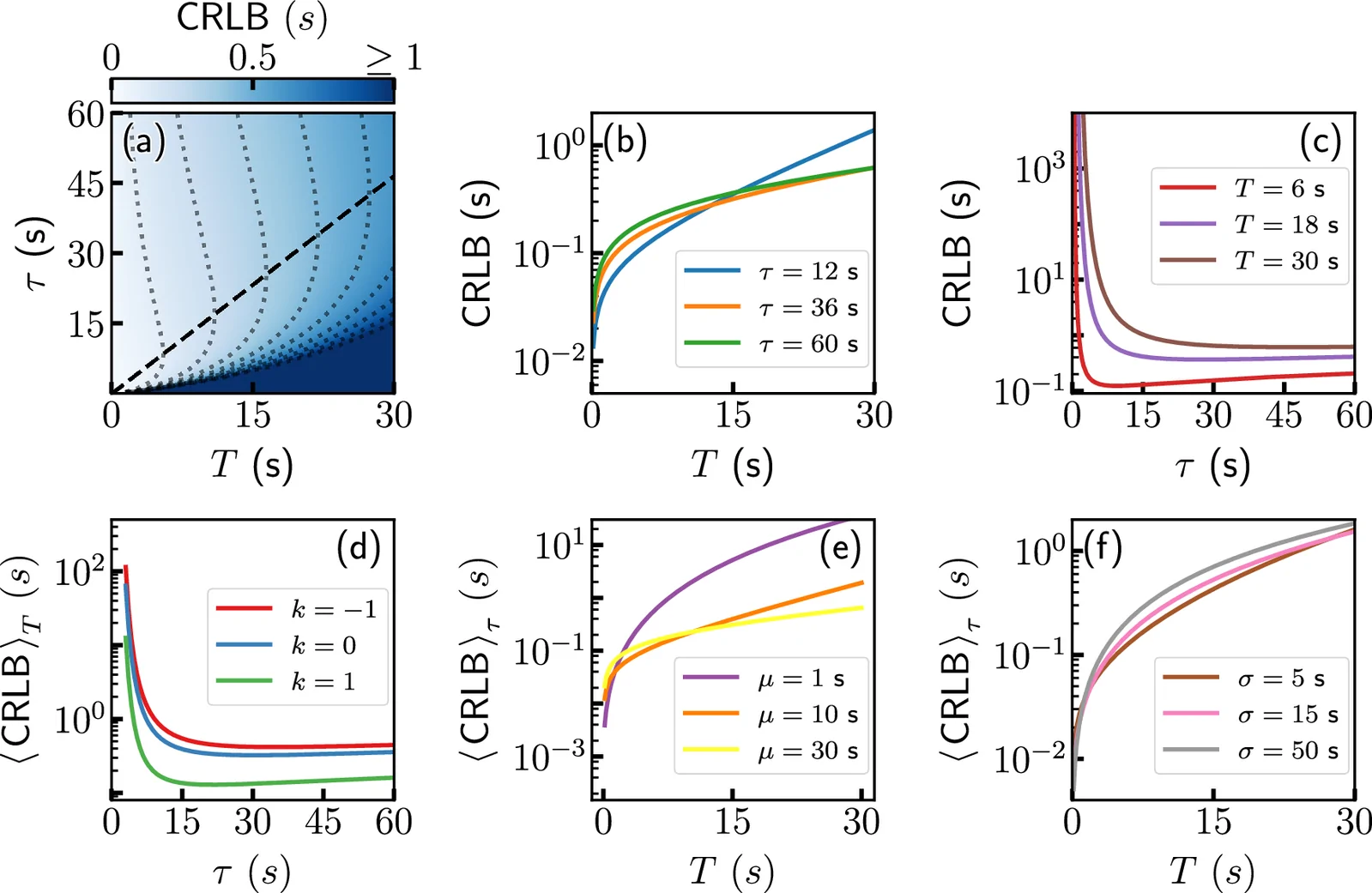
Cramér-Rao lower bound and its averages for a single interval estimated by a homogeneous VS heterogeneous population of 1000 adaptive cells. **a**–**d** Homogeneous network with a single recovery time *τ*. **e**, **f** Heterogeneous network with log-normal distributed time constants *τ* as defined by Eq. (10). **a** Dotted lines are contour lines with identical Cramér-Rao lower bound (CRLB) value. The dashed line is the solution to setting Eq. (22) to 0, i.e., the minimal CRLB for a given value of *T*. **b** Cross sections of the CRLB for various values of *τ* as a function of *T*. The error is lower for smaller values of *τ* when looking at small time intervals, but it quickly grows thereafter (e.g., blue vs. orange curves). **c** Cross sections of the CRLB for various values of *T* as a function of *τ*. The overall error is lower for smaller intervals, and the associated optimal value of *τ* increases with the length of the interval, though the CRLB increases slowly passed this optimum. **d** Averaged CRLB of a homogeneous network where the values of the time intervals are power-law distributed as defined by Eq. (10). **e** CRLB, as a function of *T*, of heterogeneous networks with constant *σ* = 1 s and different values of *μ*. **f** Same as **e**, but with constant *μ* = 10 s and different values of *σ*.

To see if the trends observed with the six previously mentioned specific cases hold in general, we computed the CRLB over a wide range of *τ* and *T* values and display them in Fig. 5a. Increasing *T* increases the error made on the estimates monotonically as shown in Fig. 5b. For a specific *T*, increasing *τ* initially has the effect of decreasing the error significantly until a minimum is reached. The increase after this minimum is then extremely slow, suggesting that a similar behavior is expected from a large non optimal *τ*. This is shown in Fig. 5c.

**Fig. 6:**
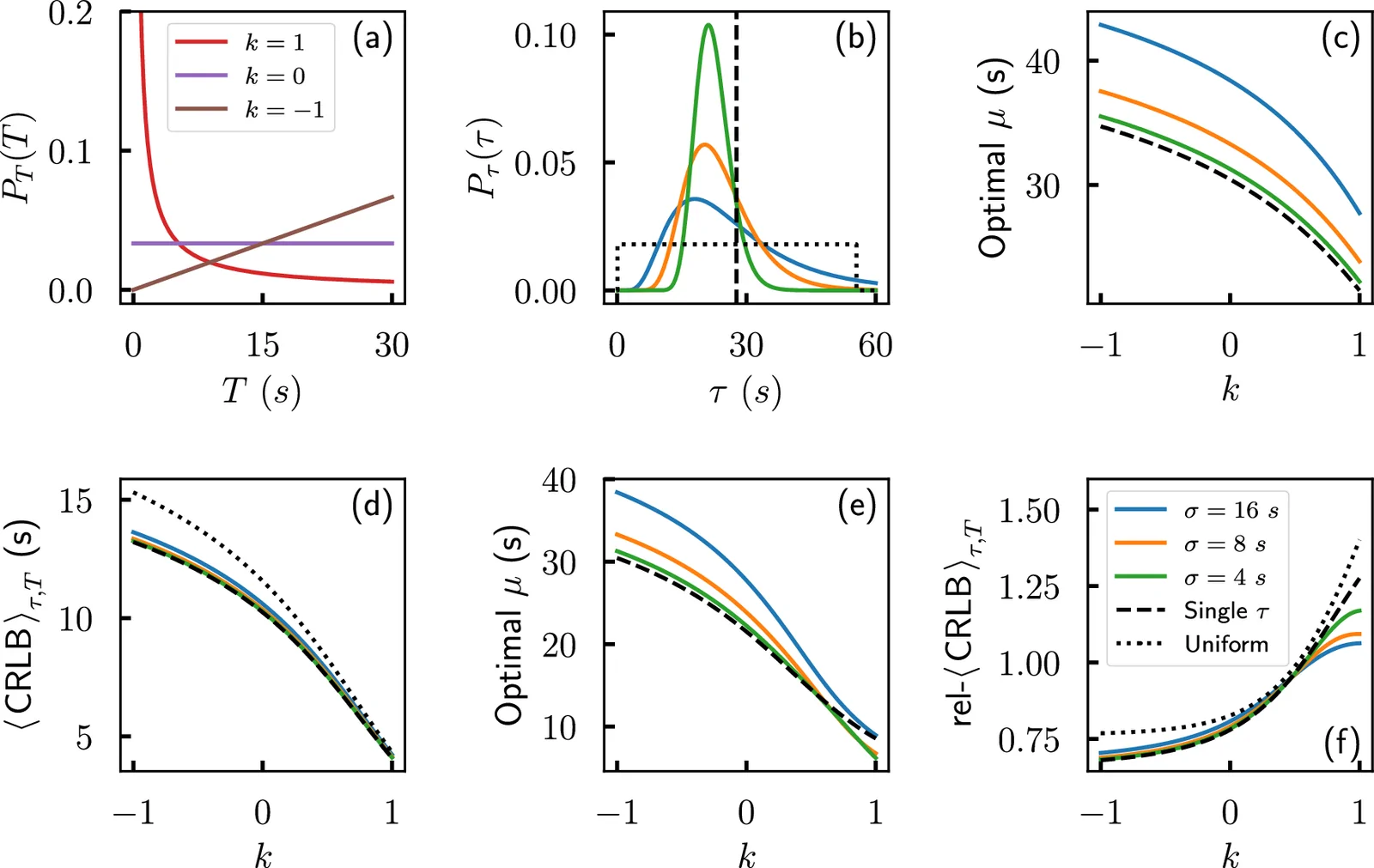
The optimal distribution of time constants can be found by minimizing the average error given a stimulus prior. **a** Power-law distributions for the continuous limit of stimulus prior as defined by Eq. (10). This is meant to represent how time intervals are expected to be distributed in nature. **b** Optimal log-normal distributions of the recovery time parameters for *k* = 1 as defined by Eq. (11). This represents the heterogeneity found in the model adaptive network. **c** Average of log-normal time constant distribution *μ* for fixed standard deviations *σ* that minimizes the average Cramér-Rao lower bound (CRLB) given a power law prior. **d** Average of the CRLB as defined by Eq. (12) of the optimal distributions of *τ* with fixed standard deviations *σ*. For optimal log-normal distributions of *τ*, the difference in performance is minimal even for a large spread of recovery times. However, a uniform distribution of *τ* makes the average error of the estimates significantly larger, indicating a sensible choice of *τ* distribution is necessary. **e** Same as **c**, but *μ* minimizes the average relative CRLB as defined by Eq. (13). **f** Same as **d**, but the average relative CRLB is minimized. Heterogeneity seems to help in the case of large *k*, i.e. when smaller values of *T* are considered more important. **b**–**f** The legend is shared among all panels.

We then looked at what happens when introducing a continuous distribution of values for the recovery times in a network, *P*_*τ*_(*τ*), combined with a continuous weight (or “prior") function for the values of the time intervals of interest *P*_*T*_(*T*). Specifically, we made the weight function a power law defined by Eq. (10) and took the distribution of recovery times to be log-normal^32,33^ defined by Eq. (11). The power law is described by the exponent *k* where a negative value gives more weight to larger time intervals while a positive value gives more weight to smaller time intervals. A value of *k* = 0 gives equal weight to all time interval values in the domain of interest $[{T}_{\min };{T}_{\max }]$. This is represented in Fig. 6a. As for the distribution of *τ*, it is described by mean *μ* and variance *σ*^2^ and is shown in Fig. 6b. The distributions are defined by

10 $${P}_{T}(T)=\alpha {T}^{-k},$$

11 $${P}_{\tau }(\tau )=\frac{1}{\tau s\sqrt{2\pi }}\exp \left(-\frac{{(\ln \tau -m)}^{2}}{2{s}^{2}}\right),$$

where *α* is such that $\int_{{T}_{\min }}^{{T}_{\max }}P(T){{{\rm{d}}}}T=1$, $m=\ln ({\mu }^{2}/\sqrt{{\sigma }^{2}+{\mu }^{2}})$ and $s=\ln (1+{\sigma }^{2}/{\mu }^{2})$ with *μ* and *σ*^2^ being the mean and variance of the *τ* distribution, respectively.

Since there is a wide range of different possible stimuli, we settled on the average of the CRLB sampled from the *P*_*T*_(*T*) distribution as a metric to measure the overall performance of the network over this range of time intervals of interest. The effect of averaging over different time intervals appears to be a flattening of the CRLB around the minimum, making large values of *τ* perform essentially equally, especially when giving more weight to large time intervals as shown in Fig. 5d.

The variance in the parameter distribution is how we introduced heterogeneity in this continuous case. To assess how it affects the performance of MLE, we first computed the CRLB using the FI of a full network containing recovery times distributed as *P*_*τ*_(*τ*) as a function of *T*. This allowed us to get an idea of the performance of the whole network given some stimulus. The average *μ* of this distribution has the most important effect on the error of the estimate as shown in Fig. 5e, while the effect of the variance *σ*^2^ is less noticeable as shown in Fig. 5f.

We then combined both averages to get the overall effect of continuous distributions which yields the expression for the average CRLB:

12 $${\left\langle {{{\rm{CRLB}}}}\right\rangle }_{\tau ,T}=\int_{{T}_{\min }}^{{T}_{\max }}\frac{{P}_{T}(T)}{\sqrt{\int_{0}^{\infty }{P}_{\tau }(\tau ){{{\mathcal{I}}}}(T;\tau ){{{\rm{d}}}}\tau }}{{{\rm{d}}}}T.$$

We also computed the average relative CRLB in a similar manner:

13 $${{{\rm{rel}}}}-{\left\langle {{{\rm{CRLB}}}}\right\rangle }_{\tau ,T}=\int_{{T}_{\min }}^{{T}_{\max }}\frac{{P}_{T}(T)}{T\sqrt{\int_{0}^{\infty }{P}_{\tau }(\tau ){{{\mathcal{I}}}}(T;\tau ){{{\rm{d}}}}\tau }}{{{\rm{d}}}}T.$$

We then found the averages *μ* that minimize these quantities for different values of *σ* and *k* and represented them in Fig. 6c, e. The values of the minima reached during the optimization of Eqs. (12) and (13) were then used to compare the minima for all specified values of *σ* and *k*. This is shown in Fig. 6d, f. The smallest possible average CRLB is reached when there is a single value of *τ* (lower black dashed line) for all values of *k* between  − 1 and 1. However, when optimizing the relative CRLB, heterogeneity is better when shorter time intervals are of greater importance (*k*~1). The improvement is small, but noticeable (see also Supplementary Fig. 1f). Homogeneity is therefore the optimal solution for estimating a sequence of a single time interval in most cases. This goes in accordance with the previously built intuition using six specific cases.

However, it should be noted that the actual difference in CRLB is not that significant between a network that is homogeneous in *τ* vs one that has a well-chosen heterogeneity in *τ*. Indeed, even for a significantly heterogeneous network with *σ* = 16 s, the maximum difference with the error made in the homogeneous case is only about 3%. It is also important to point out that not all heterogeneity is a sensible choice. For example, a completely uniform distribution of *τ* (black dotted line) yields quite a significant error. This is due to the large proportion of neurons with smaller time constants that recover quickly and hence contribute little to the estimation of larger time intervals. This small dependence of the error on the variance therefore means that a reasonable choice in the heterogeneity of time constants can be made almost independently of that for the time intervals expected to be experienced in nature.

### Heterogeneity is necessary for estimating sequences of multiple time intervals

To provide an explanation for the observed heterogeneity in real PG neurons of the electric fish, we looked at the case where a sequence of encounters produces 2 or more time intervals. The simplest such case is when there are 2 time intervals to be estimated with 2 different populations of neurons. In this case, the FI becomes a 2 × 2 matrix whose inverse gives the CRLB. For this matrix to be invertible, its determinant must be non-zero. For these 2 types of neurons, the expression of the determinant of the FI matrix can be reduced quite significantly (see subsection “Computing the determinant for a sequence of 2 time intervals" in Methods):

14 $${{{\rm{det}}}}{{{\mathcal{I}}}}=\frac{{N}^{1}{a}^{1}{N}^{2}{a}^{2}}{{\lambda }_{2}^{1}{\lambda }_{2}^{2}}{\left(\frac{\partial {x}_{2}^{1}}{\partial {T}_{1}}\frac{\partial {x}_{2}^{2}}{\partial {T}_{2}}-\frac{\partial {x}_{2}^{1}}{\partial {T}_{2}}\frac{\partial {x}_{2}^{2}}{\partial {T}_{1}}\right)}^{2}.$$

The only way for the determinant to be non-zero is for both types of cells to have either a different value of recovery time (*τ*^1^ ≠ *τ*^2^) or a different value of memory parameter (*β*^1^ ≠ *β*^2^). Another requirement is to have at least one type of cell that can encode the first time interval in the sequence, i.e., one population with *β* > 0. A different gain (*a*^1^ ≠ *a*^2^) or a different baseline activity (*c*^1^ ≠ *c*^2^) cannot make this determinant non-zero by themselves. Thus, a surprising result arises from our analyses: at least some level of heterogeneity is needed to encode two or more intervals, as opposed to the case of a single time interval.

A simple way to understand why a determinant of 0 for the FI matrix yields invalid estimates is to realize that it is a deterministic measure of a stochastic process. In other words, it is a way to describe how the estimator behaves on average. The existence of a unique solution stems only from the invertibility of the underlying set of equations, which can be summarized here as $({\lambda }_{2}^{1},{\lambda }_{2}^{2})\leftrightarrow ({T}_{1},{T}_{2})$. Therefore, if the dynamical system is not invertible in a local area of (*T*_1_, *T*_2_), then estimates of sequences in this area will give, on average, a large (infinite) error.

Although a non-zero determinant is a necessary condition to effectively retrieve two time intervals from the responses of two populations of neurons, it does not guarantee a valid solution every time. Indeed, there can be a combination of time intervals and responses for which the LL is invalid. One such case is when the average response of a population is larger than *a* + *c*, making the estimate diverge to infinity. However, due to the intrinsic variability of the response of adaptive neurons, the exact same time intervals could later yield an LL with a valid maximum. The former case rarely happens for large populations of neurons, because the averaging effect over multiple cells restrains the possible value of the average response of a population.

**Fig. 7:**
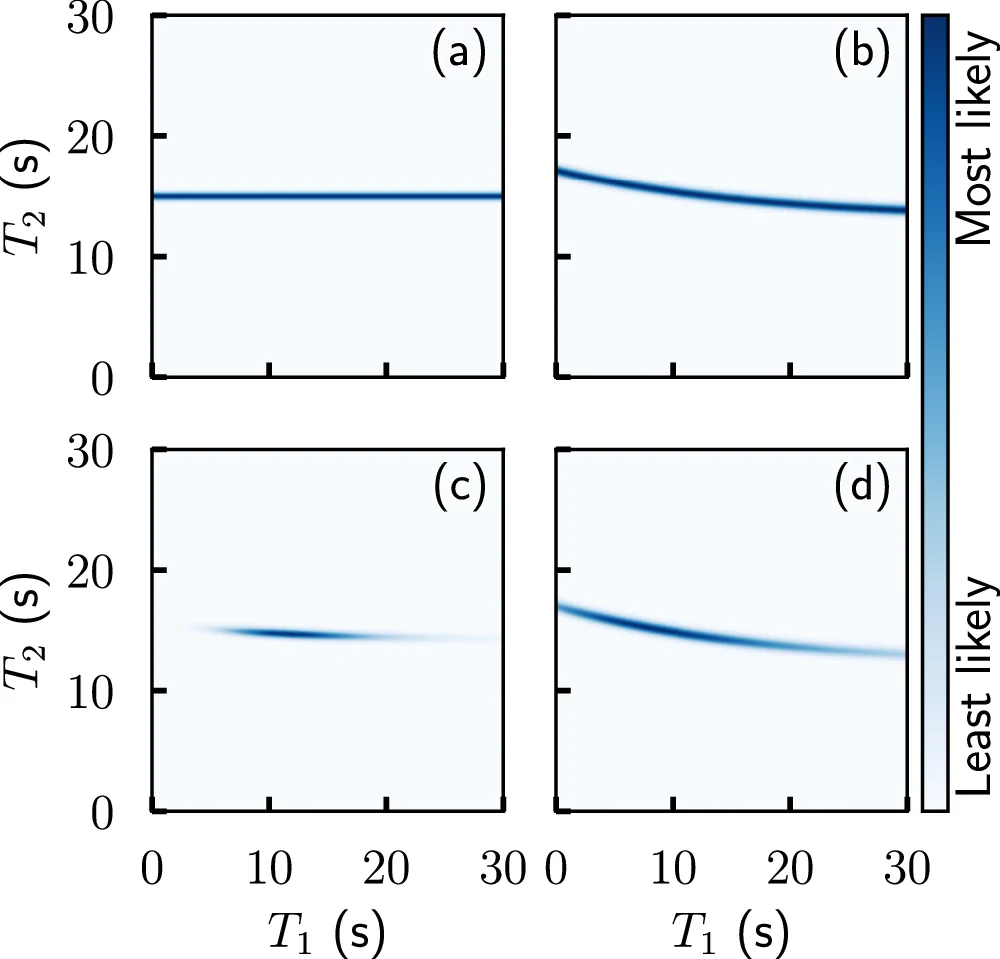
At least two different populations of neurons are necessary to estimate two time intervals. Likelihood as defined by Eq. (5) for a sequence of two time intervals from the response of adaptive cells during the last encounter. The stimuli were an interval of 10 s (*T*_1_) followed by one of 15 s (*T*_2_). **a** Single population of 1000 adaptive neurons with no memory (*β* = 0). **b** Single population of 1000 adaptive neurons with memory (*β* = 0.3). **c** Two populations of 500 adaptive neurons each. One population has memory (*β* = 0.3) while the other does not (*β* = 0). (d) Two populations of 500 adaptive neurons each. Both populations have memory (*β*_1_ = 0.3, *β*_2_ = 0.5). The lines in **a** and **b** show sequences that are equally likely, which indicates at least 2 different populations are necessary to have a single maximum. The maximum in **c** is more pronounced than the one in **d**, which indicates that having a population without memory yields more precise estimates. The x and y-axes are shared by all panels.

Because the determinant is a local measure of curvature around a specific point of (*T*_1_, *T*_2_) space, there can be a combination of different adaptive populations where the determinant is zero in a specific area of sequence space while being non-zero somewhere else. However, when combining a population with memory with one without, this phenomenon is impossible. This is because the memory-less population gives a unique solution for the latest time interval, which in turn gives only one value for the previous interval that maximizes the LL. In fact, the combination of two populations where one of them is memory-less maximizes the determinant of the FI, possibly minimizing the error made on the estimates. We illustrated the phenomenon where the existence of one memory-less population maximizes the determinant by looking at the LL of different combinations of populations in Fig. 7. The maximum of the LL looks sharpest when one of the populations has no memory. This may therefore explain why a large fraction of the cells were observed to be memory-less.

**Fig. 8:**
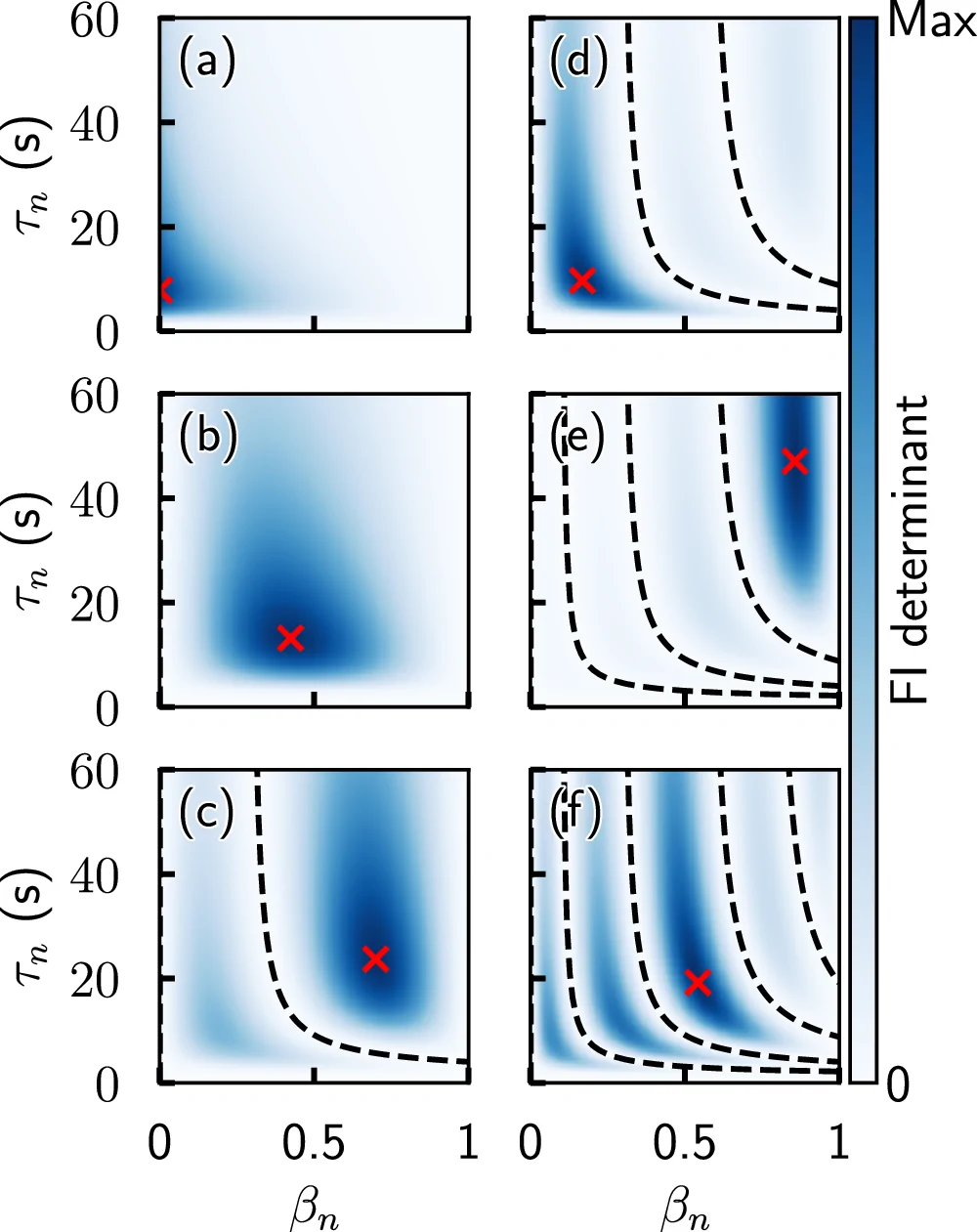
More populations of neurons are necessary to make the determinant of the Fisher information matrix non-zero as sequence increases in size. A sequence of *n* repeated time intervals of 5 s was used to compute the associated Fisher information determinant with *n* populations. Starting with *n* = 1, the parameters of the last added population were changed (*β*_*n*_ and *τ*_*n*_) while other populations were fixed at the maximum of previous determinant computations (*n*−1, *n* − 2,...,1). A determinant of zero indicates that the estimation of the sequence is impossible, which becomes more difficult to avoid as the length of the sequence to estimate increases due to the increasing number of 0 determinant areas represented by the dashed curves. (**a**) to (**f**) 1 to 6 intervals and populations, respectively. The color scheme was rescaled for each situation individually, because the units and scale of the determinant change as the sequence gets longer. The red crosses are the locations of the maxima that were used as population parameters for longer sequences and their values are shown in Supplementary Table 1. The x and y-axes are shared by all panels.

The idea of the necessity of heterogeneity can also be generalized to a sequence of more than two time intervals and populations. Here, we present a numerical argument, because this problem is currently analytically intractable. To do so, we chose a sequence of repeated time intervals of 5 s for which we compute the determinant of the FI matrix. We then proceeded systematically from single to multiple intervals and populations. We started with a “sequence" of 1 interval and an estimator with 1 population. We changed the memory parameter and the recovery time for this population and computed the FI determinant. The maximum determinant is reached when *β*_1_ = 0 and *τ*_1_ ≈ 7.84 s as shown in Fig. 8a, which is what is expected from the theory. We then added another interval to the sequence and a new population to the estimator. The parameters of the first population were set to the maximum that was previously found for the case of a single time interval. We computed the determinant while changing the values of the parameters of the added population. The determinant is always 0 when *β*_2_ = *β*_1_ = 0, and there is a maximum when *β*_2_ ≈ 0.42 and *τ*_2_ ≈ 13.2 s as shown in Fig. 8b.

We repeated the process by adding a third interval to be estimated with a third population. This time, there are two lines in the space (*β*_3_, *τ*_3_) for which the determinant is 0. They are shown in Fig. 8c as black dashed lines. The new line passes through (*β*_2_ = 0.424, *τ*_2_ = 13.2), showing that a new population identical to either previous ones cannot produce a valid estimator for 3 time intervals. There are also other parameter choices that are completely different from either previous populations, but still lead to a non-invertible FI matrix. In other words, these combinations of unique populations will still lead to a degeneracy in the estimation of (*T*_1_, *T*_2_, *T*_3_). In that case, an equivalent observation is that the CRLB diverges to infinity, indicating that different values of time intervals are equally likely. This phenomenon can be observed for longer sequences as well (4 to 6 time intervals shown in Fig. 8d to f, respectively). The choice of population parameters therefore becomes increasingly difficult as the length of the sequence increases, due to the addition of new “invalid lines" in (*β*, *τ*)-space. We also expect this pairwise restriction on the populations to become more complex as the variability in sequences changes (e.g. when they are not simply repeated identical intervals).

The values of the different maxima and their associated parameters are shown in Supplementary Table 1. When studying the problem for one or two intervals, we could visualize the performance of an estimator through the error of the individual elements of the sequence (*T*_1_ and/or *T*_2_). When increasing the dimensionality of the problem, however, this becomes impossible. We therefore calculated the CRLB on the sum of the time intervals in the sequence for each maximum of Fig. 8. To do so, we recall that, assuming no bias, the covariance matrix is bounded by ${{{\rm{cov}}}}[{T}_{ij}]\ge {[{{{{\mathcal{I}}}}}^{-1}]}_{ij}$. Since the variance for the sum *Z*_*n*_ = *T*_1_ + *T*_2_ + ⋯ + *T*_*n*_ is ${\sum }_{i,j=1}^{n}{{{\rm{cov}}}}[{T}_{ij}]$, we get the CRLB on the sum of intervals with

15 $${{{{\rm{CRLB}}}}}_{{Z}_{n}}\ge \sqrt{{\sum }_{i,j=1}^{n}{\left[{{{{\mathcal{I}}}}}^{-1}\right]}_{ij}}.$$

This is a useful metric to treat as a performance indicator for sequences of arbitrary length. Even when using population parameters that maximize the FI determinant, we see that the value of ${{{{\rm{CRLB}}}}}_{{Z}_{n}}$ increases exponentially (see Supplementary Fig. 2), which should be taken as a sign that estimates become increasingly hard to generate as sequences grow in size.

**Fig. 9:**
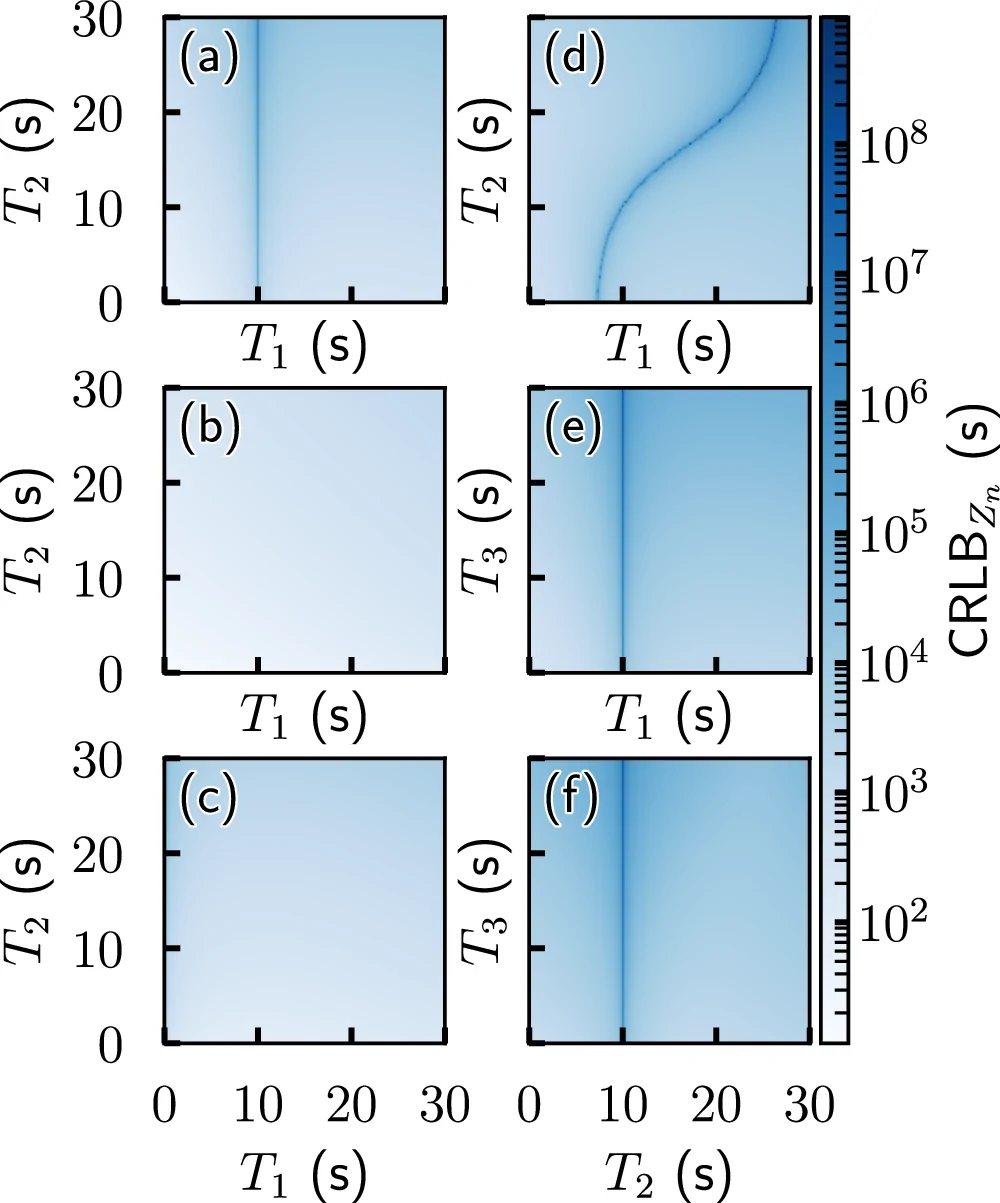
A wrong selection of populations leads to sequences that are impossible to estimate. **a**–**c** Cramér-Rao lower bound (CRLB) on the sum of intervals as defined by Eq. (15) for a sequence of 2 time intervals and 2 populations. **a** Two memory populations with different memory parameters and time constants (*β*_1_ = 0.4, *β*_2_ = 0.7, *τ*_1_ = 15 s, *τ*_2_ = 8.2 s) can yield a zero determinant for some specific time interval values. Here, the parameters are such that the Fisher information matrix is never invertible when *T*_1_ = 10 s and thus leads to an infinite CRLB in that case. **b** A memory-less population combined with a memory population (*β*_1_ = 0, *β*_2_ = 0.4, *τ*_1_ = *τ*_2_ = 15 s) yields a finite CRLB for all values of time intervals. **c** Two memory populations with different time constants (*β*_1_ = *β*_2_ = 0.4, *τ*_1_ = 15 s, *τ*_2_ = 8.2 s) also yield a finite CRLB for all values of time intervals. **d**–**f** CRLB of the sum of intervals for a sequence of 3 time intervals and 3 populations. For all three panels, the population parameters are the same (*β*_1_ = 0.2, *β*_2_ = 0.4, *β*_3_ = 0.7, *τ*_1_ = 25.6 s, *τ*_2_ = 15 s, *τ*_3_ = 8.2 s). Each panel is a slice of (*T*_1_, *T*_2_, *T*_3_)-space with constant *T* = 10 s for the interval not shown on the axes. Only the combination of *T*_1_ and *T*_2_ values affects whether the CRLB is finite. When a sequence starts with specific values of *T*_1_ and *T*_2_ as in **d**, an accurate estimation of the sequence is impossible. In terms of numerical values, the x and y-axes are shared by all panels.

As previously mentioned, the degeneracy phenomenon is dependent on the local nature of the FI. A combination of populations that cannot estimate a specific sequence of time intervals may still be able to lift the degeneracy for another sequence. For example, two populations with different memory parameters (*β*_1_ = 0.4, *β*_2_ = 0.7) and time constants (*τ*_1_ = 15 s, *τ*_2_ = 8.2 s) will make any sequence of two time intervals beginning with *T*_1_ = 10 s impossible to estimate accurately, while still performing adequately for other sequences, as shown in Fig. 9a. Note that this is simply by construction. We chose the values of *β*_1_, *β*_2_ and *τ*_1_, then calculated the value of *τ*_2_ that would give a zero determinant when *T*_1_ = 10 s using Equation (14) to illustrate the problem.

However, having a memory-less population combined with a memory population (*β*_1_ = 0, *β*_2_ = 0.4) with identical time constants (*τ*_1_ = *τ*_2_ = 15 s) completely eliminates this degeneracy as shown in Fig. 9b. When two populations have the same non-zero memory parameter (*β*_1_ = *β*_2_ = 0.4) and different time constants (*τ*_1_ = 15 s, *τ*_2_ = 8.2 s), the degeneracy is also lifted as shown in Fig. 9c. In other words, the degeneracy problem and thus the decoding problem can easily be circumvented for a sequence of two time intervals.

Increasing the number of time intervals to 3 makes it more difficult to remove the degeneracy, because there is no other choice than to have at least 2 populations with different non-zero memory parameters. In this case, a combination of parameters not carefully selected makes it impossible to estimate some sequences. Even when all parameters are different (e.g., *β*_1_ = 0.2, *β*_2_ = 0.4, *β*_3_ = 0.7, *τ*_1_ = 25.6 s, *τ*_2_ = 15 s, *τ*_3_ = 8.2 s), the responses of those populations cause different time interval sequences to have the same likelihood just like in the homogeneous case. This is shown in Fig. 9d–f. It is therefore necessary to have a well selected heterogeneity in the adaptation parameters to cover a wide range of sequences.

### Heterogeneity helps machine learning-based decoders

To make sure the necessity of heterogeneous adaptation is not just a result of the idealized context of MLE, we explored the performance of two additional estimators. The first estimator is a multilayer perceptron (MLP), and the second one is a reservoir computer (RC) made of a recurrent neural network (RNN). We chose such estimators to stay true to the anatomy of the brains of weakly electric fish, where the downstream structure receives independent inputs from PG. Therefore, the input to the decoders is the adaptive responses of *N* = 1000 PG cells during the last encounter in a sequence, and they were trained to estimate the last 2 intervals. For clarity, we refer to each pair of intervals used for training as the “latest" (*T*_*n*_) and the “previous" (*T*_*n*−1_) intervals. We checked whether heterogeneity in the PG dynamics could enhance the prediction in these downstream networks. To do so, we chose an input made of memory-less cells (*β* = 0) combined with a number ⌊*p*_*m*_*N*⌋ of memory cells with a memory parameter *β*_*m*_ ≥ 0, where *p*_*m*_ ∈ [0, 1] is the proportion of memory cells (see subsection “Multilayer perceptron and reservoir computer for time interval estimation" in Methods).

**Fig. 10:**
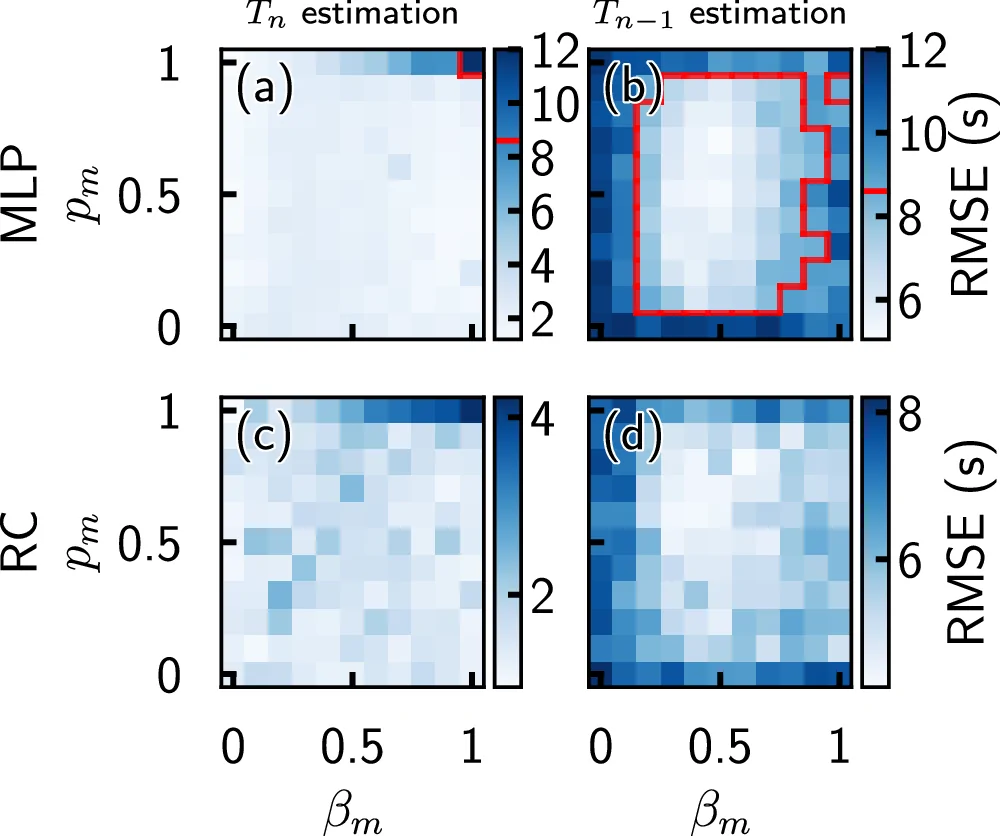
Error of multilayer perceptron and reservoir computer estimators shows that heterogeneity helps with performance. Root mean square error (RMSE) of the latest time interval *T*_*n*_ and the previous one *T*_*n*−1_ estimates during the presentation of random sequences of time intervals. The input to the multilayer perceptron (MLP) and reservoir computer (RC) is from *N* = 1000 simulated adaptive neurons with varying degrees of heterogeneity with respect to the memory *β* of their adaptation process (see Methods). A proportion ⌊*p*_*m*_*N*⌋ have a memory parameter of *β*_*m*_ and a time constant of *τ* = 30 s while the other cells have no memory (*β* = 0) and a time constant of *τ* = 15.55 s. All input cells have a gain *a* = 10 and baseline *c* = 0. **a** Error of trained MLPs for each *β*_*m*_ and *p*_*m*_ pairs made on the estimate of the latest time interval. Over all compositions of cells, there is an average RMSE of 2.0 s. **b** Error of the same MLPs as in **a** made on the estimates of the previous time interval. For a homogeneous composition of cells (lower, left and upper edges of the panel), the average of the RMSE is of 11.0 s, which is no better than chance (see Supplementary Note 3). For the heterogeneous case, the average RMSE is 7.6 s with a minimum of 5.0 s when *β*_*m*_ = 0.5 and *p*_*m*_ = 0.7. **c** Error of trained RCs for each *β*_*m*_ and *p*_*m*_ pairs made on the estimate of the latest time interval. Over all compositions of cells, there is an average RMSE of 1.6 s. **d** Error of the same trained RCs as in **c** made on the estimates of the previous time interval. For a homogeneous composition of cells, the average of the RMSE is of 7.3 s for the memory-less case (lower and left edges of the panel), while the average of the RMSE is of 6.8 s when *β*_*m*_ > 0 (upper edge of the panel). When the composition is heterogeneous, the average of the RMSE is of 5.5 s with a minimum of 4.3 s when *β*_*m*_ = 0.6 and *p*_*m*_ = 0.8. The red lines delimit areas where the RMSE is lower than chance (<8.6 s). Only MLP estimators have compositions of (*β*_*m*_, *p*_*m*_) yielding a performance worse than chance. The x and y-axes are shared by all panels.

For both the MLP and the RC, decoding the latest time interval can be done regardless of heterogeneity as shown in Fig. 10a, c. Performance is similar for most compositions of adaptive memory, although a memory-less network (*β*_*m*_ = 0 or *p*_*m*_ = 0) appears slightly better on average for the case of the MLP with an average RMSE of 1.3 s compared to the average of 1.6 s in the heterogeneous case. This configuration was tested multiple times and can be seen in the bottom row across *β*_*m*_ values and left column across *p*_*m*_ values of all panels of Fig. 10. The variability of the RMSE values comes from the random initialization of the MLP and the RC, and the different training and testing sequences for each point where *β*_*m*_ = 0 or *p*_*m*_ = 0.

Inferring the previous time interval is also impossible in the case of the MLP when the input comes from a homogeneous population, regardless of the memory parameter (see Fig. 10b, bottom, left and top edges of the panel). In fact, the average error in this case is around 11.0 s when estimating the previous time interval, which is no better than always predicting an optimized constant value that yields an error of around 8.6 s (see Supplementary Note 3). This is in accordance with the degeneracy effect that comes with a homogeneous adaptive response: different pairs of time intervals are equally likely. A heterogeneous composition of PG cells gives an average error of 7.6 s with a minimum of 5.0 s around *β*_*m*_ = 0.5 and *p*_*m*_ = 0.7 when estimating the previous interval. However, this minimum is wide, and similar error values are found for similar values of *β*_*m*_ and *p*_*m*_.

The RC estimator can still estimate the previous time interval better than chance with an average error of 7.2 s in the homogeneous case, as shown on the bottom, left, and top edges of Fig. 10d. Similarly to the MLP, a heterogeneous PG input gives a lower average error of 5.5 s with a minimum of 4.3 s around *β*_*m*_ = 0.6 and *p*_*m*_ = 0.8. Again, values of *β*_*m*_ and *p*_*m*_ close to this minimum give similar RMSE values. This further confirms that heterogeneous adaptation helps encode time sequences, since it makes crucial timing information available to more sophisticated downstream decoders.

When *β*_*m*_ = 1 and *p*_*m*_ = 1, the intensity of the input does not contain information about the sequence. Since *β*_*m*_ = 1 for all cells, there is no drop in resources during an encounter. Therefore, even if the initial amount of resources was not already full (*x* = 1), it would quickly reach that point, making the responses during each encounter share the same statistics regardless of the time elapsed between events. In that case, PG cells become non-adaptive cells responding to any event in the same manner. This is reflected accordingly for both the MLP and the RC. Indeed, the MLP has no information about previous time intervals and therefore cannot infer the sequence, not even the latest interval. However, because the RC has recurrent dynamics and therefore some memory capacity, the non-adaptive cells still allow it to encode more than one time interval in a sequence, as can be seen in the upper right corner of Fig. 10c, d. In fact, we used the performance of the RC when *β*_*m*_ = 1 and *p*_*m*_ = 1 to calibrate the values of some parameters of the reservoir (see subsection “Multilayer perceptron and reservoir computer for time interval estimation" in Methods and Supplementary Fig. 3).

Another feature that both the MLP and the RC share with MLE is the decrease in performance for a homogeneous network of PG cells as the memory parameter *β*_*m*_ increases when estimating the latest time interval, as shown on the upper edges of Fig. 10a, c. This is because the response intensity carries progressively less timing information as *β*_*m*_ increases due to the reduced dynamical range of the *x* resource variable.

We also tested RCs with longer time constants *τ*_in_ and *τ*_*R*_ related to the decay of the input signal and the decay of the activity of a unit in the RNN, respectively. In that case, the error is generally lower for both the latest and previous time intervals decoding (see Supplementary Fig. 4). Since the input composition and the coupling to the RNN are the same as the shorter time constant case, the only reason this estimator performs better than the RCs shown previously is its increased memory capacity due to the larger values of *τ*_in_ and *τ*_*R*_. A memory-less homogeneous input gives an average error of 6.4 s (lower and left edges of Supplementary Fig. 4b) while one with only memory cells gives an average error of 5.5 s (upper edge of Supplementary Fig. 4b) when estimating the previous interval. The average error in the heterogeneous case is 4.6 s with a minimum of 3.5 s around *β*_*m*_ = 0.9 and *p*_*m*_ = 0.7. However, this minimum is not as sharp as in the RCs with shorter time constants, and multiple values of *β*_*m*_ and *p*_*m*_ yield similar errors. Nevertheless, heterogeneous adaptation in the input signal still significantly improves performance.

## Discussion

Various mechanisms across brain regions have been proposed that enable the encoding of time^34,35^, involving clocks, activity rampings^36^, neural sequence storage^37^, state-dependent networks^38^, pulse-counting^39^, oscillator-based models^40^, and sequences of neuronal assemblies^14–16^. The storage of interval duration between current and future times, known as prospective timing, may engage neural timers that measure the passage of time^35^. During ramping, timing information is possibly encoded in the increasing firing rates of neurons between start and stop times. Sequence memory could use high-dimensional neural activity trajectories to represent times or time intervals. Analogs of time and ramping cells emerge in deep reinforcement learning models performing simulated interval memory tasks, but these models are agnostic to the actual mechanisms^41^. Our time-stamping mechanism resembles the ramping activity model in that neuronal firing probability builds over time since an event. However, the biophysical origin of its long recovery time scale is unknown. Nevertheless, the relationship between time interval estimation and spatial learning is evident. Numerous strategies have been put forward for spatial learning in teleost fish alone^42^, for which sensory information needs to be combined and then stored in the dorsolateral pallium (DL), much like what happens in the mammalian hippocampus. Path integration, the use of sensory information to estimate distance traveled, likely benefits from downstream combinations of information about intervals, place, heading direction, and velocity from the lateral line organs^19^, making the activity of PG crucial for spatial learning.

Adaptation has been extensively studied for various stimuli^43^ and on a wide range of time scales^44^. For example, stimuli activating visual circuitry give rise to adaptation to contrast, orientation, or motion of the stimulus^45–47^. Closely related paradigms attempt to explain the role of adaptation^48^, such as efficient coding, by which neurons optimize the representation of stimuli given limited resources^49^. Another such paradigm is predictive coding, by which neural systems need to make inferences about possible future stimuli^50^. It has also been proposed by Hopfield in 1996 that sequence classification can be helped by adaptation to ignore non-critical time differences in stimuli^51^. Although similar to what was presented here, the adaptation code Hopfield proposed had cells each respond to one type of syllable, in contrast to PG cells that respond to all encounters. Moreover, the adaptation model we propose is for the burst intensity, rather than a continuous reduction of firing rate intensity. Nonetheless, a bridge between both models can be made, since encoding sequences of time intervals, like we propose, can be used thereafter as a path classifier. Indeed, being able to encode these sequences accurately enables identifying the trajectory taken while disregarding time warps incurred by moving at different velocities, much like the identification of words spoken at different speeds that Hopfield proposed.

The use of MLE was made for the sake of simplicity. It assumes minimal knowledge about how PG cells behave and what kind of prior information electric fish might have about the distribution of time intervals encountered in their natural habitat. Here we have extended the MLE to include such priors, parametric heterogeneity, and memory effects. We do not claim that downstream structures from PG actually implement MLE, though some sort of Bayesian interpretation could be applied^52,53^. In fact, DL and neighboring areas are highly recurrent^54^. It is therefore suspected that interval sequence information can actually be stored in an attractor network, and that more accurate information about sequences may be available than our theory suggests.

We presented a joint dynamical and Bayesian analysis of interval encoding, focusing first on a single time interval between two encounters, and ending with a proof that heterogeneity is needed to encode two or more intervals. MLE combined with Fisher and Cramér-Rao metrics allowed us to quantify how well intervals are represented with the adaptive time-stamp mechanism, given the strong parametric variability, especially the presence of cells with and without memory.

For a single interval, it is best to have no memory beyond the most recent interval, i.e., *β* = 0. This is intuitively satisfying, as it leaves all of the cell’s resources to encode the latest interval. Although a large proportion of the measured PG neurons had no memory, a significant number (~67%) of them had *β* > 0^19^. This suggests that a compromise is made between encoding the latest time interval and encoding the previous ones. No baseline activity (*c* = 0) is also optimal. This may seem to be a stringent requirement, yet most cells were silent between encounters (*c* < 5 spikes for 80% of cells)^19^. In fact, some of them had a negative value for this parameter *c* (which acts like a bias), meaning they were inactive unless a strong enough stimulus could activate them, such as at a long interval. Such a silent coding property has been explored^55^, but a deeper analysis of this bias effect is beyond the scope of this paper. For the gain *a* to be as large as possible is also intuitive, as it is a simple way to increase the dynamic range of the cell. Finally, for any specific value of the current time interval *T* to be estimated from neural activity, there is an optimal recovery time constant *τ* ≈ 1.55*T*. A similar kind of result can be found for any distribution of time interval values. This prompted the exploration of heterogeneity in *τ*. We hypothesized that the optimal parameters should be those that minimize the average CRLB over the interval prior.

We initially introduced heterogeneity using six specific cases of combinations of *T* and *τ* to build intuition about the role of heterogeneity. These cases suggested that a homogeneous network was best for encoding a single time interval. This was further confirmed in the more general setting where network heterogeneity is modeled by a log-normal distribution, and the CRLB is averaged over a power-law interval prior. A suitably chosen heterogeneous network can also have a relatively small average CRLB for a single interval, a desirable property that clearly need not be sacrificed for multiple interval estimation, given the surprising requirement of heterogeneity to encode multiple intervals that is demonstrated. Moreover, we argue that the advantage of a heterogeneous distribution of time constants when minimizing the relative CRLB is minimal. Indeed, having an average relative CRLB of 106% (largest heterogeneity used when *k* = 1) instead of 128% (homogeneous case when *k* = 1), as shown in Fig. 6f is not a practical advantage for such short time intervals.

Our evolving intuition was that more than one value of the recovery time constant is needed to optimally decode a wide range of time intervals, based on the finding that a specific value of *τ* minimizes the CRLB for a specific *T* or distribution of *T*. However, averaging the CRLB over a simple prior distribution for *T* does not yield additional optimal values of *τ*, which may appear counter-intuitive. Although not proven here, our numerical explorations suggest that the CRLB is a convex function of $\left\{{\tau }^{1},{\tau }^{2},...\right\}$, implying a global minimum. The definition of the CRLB implies that this global minimum is at the point where *τ*^1^ = *τ*^2^ = . . . Moreover, averaging over different values of *T* simply has the effect of changing the position of this global minimum, because the sum of convex functions remains convex. This convexity argument makes it easier to understand how a homogeneous network is optimal when minimizing the average CRLB over a range of time intervals, no matter the prior distribution *P*_*T*_(*T*). We also suspect that the error metric is not convex when looking at the relative CRLB for shorter time intervals, making their nonlinear combination when averaging over different time intervals generate additional minima.

Only when analyzing sequences of 2 or more intervals does heterogeneity become necessary. This was proven “in principle" and is an important first step that needs to be followed by calculations of what that heterogeneity should be, including any correlated variability between parameters, to best encode interval sequences. In that direction, we presented different numerical situations where diversity is necessary using the FI determinant and related measures. Indeed, we were able to show that a sensible choice for the parameters of different populations is needed for the estimation problem to be invertible. We showed how an ill-selected combination of adaptation parameters makes some specific sequences of 2 or 3 time intervals impossible to differentiate from each other. We expect a similar effect to happen when increasing the length of the estimated sequence, making it more difficult to find a network of adaptive neurons capable of estimating all time intervals – and combinations thereof – of interest.

One way to solve this would be to increase the number of different populations for the same sequence length. More heterogeneity helps reduce the degeneracy, as a sub-combination of populations incapable of estimating some sequence can be helped by a new population whose degeneracy lies elsewhere. This is effectively what happens in reality, as the parameters of the cells in PG are distributed continuously and not as multiple sets of populations of identical neurons. This problem is, however, quickly eclipsed by the fact that the error made on the estimates grows exponentially fast as the sequence to decode increases in size, regardless of heterogeneity.

We remind the reader of the relation between the magnitude of the determinant of the FI matrix and the CRLB. This was done in order to better appreciate why conditions leading to a null determinant correspond to degeneracy (the problem is not uniquely invertible). Furthermore, they remind us of the difference between a null determinant and one that is close to zero; the former formally implies that intervals can’t be uniquely decoded, while the latter suggests that it may be difficult numerically, and more so with downstream noisy neural wetware.

To move beyond the idealized MLE framework, we trained an MLP and an RC to recover the last two time intervals (the “previous" followed by the “latest") from the adaptive response of PG cells. Both estimators were trained with different compositions of adaptive input. For both networks, the latest interval could be retrieved with low error regardless of heterogeneity. While the MLP required heterogeneity in the responses to estimate the previous interval, just like for MLE, the RC was capable of doing so without, due to its recurrent dynamics. This is further amplified by increasing the time constants of the RC, which decreases the overall error and diminishes the advantage of a diverse input (see Supplementary Fig. 4), hinting at a better memory capacity. If it existed, an RC with perfect memory would not require any adaptation (heterogeneous or otherwise), as this additional timing information would become redundant. However, under realistic assumptions where recurrent memory is limited, such as the pallium of the fish, its interplay with heterogeneous adaptation is an important question and should be investigated further.

Heterogeneity in neural networks has been shown to encode a wide range of time scales^27,56^. It is also widely accepted as a good strategy to increase performance in different tasks, especially time-related ones^28,57^. The results presented for the single interval case suggest otherwise. As such, the fact that recorded cells show a wide range of time constants and memory parameters^19^ implies that the PG population is meant to encode sequences of more than one time interval. Adaptation processes with memory carry more information compared to those that reset upon spiking due to noise-shaping, which raises the possibility that the memory cells have a built-in noise reduction mechanism^58^. It is also of interest to extend our model to include synaptic plasticity, as that can enable single-cell sequence anticipation and responses to unexpected inputs^12^.

## Methods

All numerical simulations were done in the *Julia* programming language.

### Computing the Fisher information and Cramér-Rao lower bound

We first derived the expression for the FI with the general formulation. Recall that, for an estimator with LL $\ell (\left\{{\theta }_{i}\right\})$ and parameters $\left\{{\theta }_{i}\right\}$, the FI is given by

16 $${\left[{{{{\mathcal{I}}}}}_{\theta }\right]}_{ij}=-E\left[\frac{{\partial }^{2}\ell }{\partial {\theta }_{i}\partial {\theta }_{j}}\right],$$

where *E*[ ⋅ ] is the expectation value over the observable data (here, the response of adaptive neurons). In the case of time interval estimation through MLE, the FI for one cell is given by

17 $${\left[{{{{\mathcal{I}}}}}_{T}\right]}_{ij} 	 = -{E}_{{R}_{n}}\left[\frac{\partial }{\partial {T}_{i}}\left(\left(\frac{{R}_{n}}{{\lambda }_{n}}-1\right)\frac{\partial {\lambda }_{n}}{\partial {T}_{j}}\right)\right],\\ 	 = -{E}_{{R}_{n}}\left[\left(\frac{{R}_{n}}{{\lambda }_{n}}-1\right)\frac{{\partial }^{2}{\lambda }_{n}}{\partial {T}_{i}\partial {T}_{j}}-\frac{{R}_{n}}{{\lambda }_{n}^{2}}\frac{\partial {\lambda }_{n}}{\partial {T}_{i}}\frac{\partial {\lambda }_{n}}{\partial {T}_{j}}\right],\\ 	= \frac{1}{{\lambda }_{n}}\frac{\partial {\lambda }_{n}}{\partial {T}_{i}}\frac{\partial {\lambda }_{n}}{\partial {T}_{j}},$$

since ${E}_{{R}_{n}}[{R}_{n}]={\lambda }_{n}$, by definition. For a network of multiple adaptive neurons, the FI simply becomes the sum of the FI of individual cells. This is a consequence of assuming that the adaptive neurons in PG are independent. From this, we find the FI for the complete network to be given by Eq. (8). The CRLB matrix is then given by the inverse of the FI matrix as shown in Eq. (7). In the case of a single time interval, we obtain a scalar value for both. It’s worth noting that, although going from a single to multiple cells in terms of FI can be done by simply adding to the FI matrix, the same is not true for the CRLB. The sum needs to be done prior to inverting the FI matrix.

### Maximizing the Fisher information for a specific time interval

For a single time interval and neuron, we can expand Equation (17) to

18 $${{{\mathcal{I}}}}=\frac{{a}^{2}{(1-\beta {x}_{0})}^{2}{e}^{-2T/\tau }}{{\tau }^{2}{\left[a(1-{e}^{-T/\tau }(1-\beta {x}_{0}))+c\right]}_{+}}.$$

Assuming *c* ≥ 0 (which is the case for 80% of neurons in PG^19^), the linear rectification can be dropped. To look at the behavior of the FI with respect to the parameters, we looked at its partial derivatives. First, the derivative with respect to the baseline activity *c* gives

19 $$\frac{\partial {{{\mathcal{I}}}}}{\partial c}=-\frac{{a}^{2}{(1-\beta {x}_{0})}^{2}{e}^{-2T/\tau }}{{\tau }^{2}{\left[a(1-{e}^{-T/\tau })+c\right]}^{2}}.$$

For any admissible values of the parameters, Eq. (19) is always negative. Therefore, the FI decreases as *c* increases and can be maximized by making *c* as small as possible as shown in Fig. 2c. This means that the FI in this model deteriorates as the spontaneous activity increases. Since we assume *c* ≥ 0, the optimal value for *c* for any time interval value is *c* = 0.

We next looked at the derivative with respect to the memory parameter *β*. When setting *c* = 0, we have

20 $$\begin{array}{rcl}\frac{\partial {{{\mathcal{I}}}}}{\partial \beta }&=&-\frac{a(1-\beta {x}_{0}){x}_{0}{e}^{-2T/\tau }}{{\tau }^{2}\left[1-{e}^{-T/\tau }(1-\beta {x}_{0})\right]}\\ &\times &\left[2+\frac{(1-\beta {x}_{0}){e}^{-T/\tau }}{1-{e}^{-T/\tau }(1-\beta {x}_{0})}\right].\end{array}$$

Again, for any valid values of the parameters, Eq. (20) is negative. The optimal memory parameter is therefore *β* = 0 when estimating a single time interval. After simplifying the expression with *c* = *β* = 0, it is straightforward to show that the derivative with respect to the gain parameter *a* is always positive. Indeed, we have

21 $$\frac{\partial {{{\mathcal{I}}}}}{\partial a}=\frac{{e}^{-2T/\tau }}{{\tau }^{2}(1-{e}^{-T/\tau })}.$$

The FI is therefore unbounded with respect to *a*, i.e., a value of the gain as large as possible is optimal.

Finally, computing the derivative with respect to the recovery time constant *τ* yields a subtler result. In that case, we obtain the expression

22 $$\frac{\partial {{{\mathcal{I}}}}}{\partial \tau }=-\frac{a}{{\tau }^{3}\left({e}^{2T/\tau }-{e}^{T/\tau }\right)}\left[2-\frac{T\left(2{e}^{2T/\tau }-{e}^{T/\tau }\right)}{\tau \left({e}^{2T/\tau }-{e}^{T/\tau }\right)}\right].$$

In contrast to the previous parameters, the derivative of the FI with respect to *τ* is not always positive or negative. There is a maximum which can be found by setting the derivative to 0. With a change of variable *x* = *T*/*τ* and using Newton’s root finding algorithm, the optimum is found at the point *τ* ≈ 1.55*T*.

### Computing the root mean square error of the estimator

The need to be careful when using the FI and CRLB is well-known. This is because the FI is valid only for unbiased estimators. In the limit of an infinite number of responses, the estimator is expected to have exactly the same error as the CRLB. However, the minimum number of responses needed to effectively assume there is no bias in the estimator is not a trivial task^59^. We therefore needed to compute the RMSE of the estimator with a Monte Carlo simulation and compare it to the CRLB. To do so, we generated the response of *N* adaptive neurons resulting from encounters separated by an interval *T*. From these *N* responses, we maximized the LL given by Eq. (6) with respect to *T* to find the estimate *T*^*MLE*^. This process was repeated for a given number of samples *s* such that the RMSE is given by

23 $$RMSE(T)=\sqrt{{\sum }_{k=1}^{s}{\left(T-{T}_{k}^{MLE}\right)}^{2}.}$$

Convergence is assumed when the difference in the average and variance of *T*^*MLE*^ after 100 new estimates is smaller than 10^−6^. This allowed us to compare the actual error trend when estimating a single time interval (RMSE) with what we used to optimize the adaptation parameters (CRLB). For large enough networks, both quantities are essentially the same as shown in Fig. 4.

### Averaging the Cramér-Rao lower bound over multiple time interval values

When looking at a single value of time interval *T*, optimizing the FI is equivalent to optimizing the CRLB. However, the optimum becomes different when multiple values of time intervals are considered. To show that this is the case, we can simply look at the uniformly weighted sums for 2 time interval values ${T}_{1}^{* }$ and ${T}_{2}^{* }$ with a single type of cell for both the FI and the CRLB as defined by Eqs. (24) and (25), respectively. The nonlinear sum of both quantities in Equation (25) causes the optimal *τ* to be at a different location than for Eq. (24):

24 $${{{{\mathcal{I}}}}}_{{{{\rm{total}}}}}=\frac{1}{2}\left({{{\mathcal{I}}}}({T}_{1}^{* },\tau )+{{{\mathcal{I}}}}({T}_{2}^{* },\tau )\right),$$

25 $$CRL{B}_{{{{\rm{total}}}}}=\frac{1}{2}\left(\frac{1}{\sqrt{{{{\mathcal{I}}}}({T}_{1}^{* },\tau )}}+\frac{1}{\sqrt{{{{\mathcal{I}}}}({T}_{2}^{* },\tau )}}\right).$$

Therefore, one has to choose for which of these two quantities the actual value of *τ* is optimal for. We argue that the choice of the CRLB is a natural one, since it lets us compare the actual error computed through Monte Carlo simulations to what is found from the FI. Moreover, as mentioned in subsection “Heterogeneity is necessary for estimating sequences of multiple time intervals" in Results, for sequences of multiple time intervals, the CRLB of the total time traveled (sum of all time intervals in the sequence) is the sum of all elements in the inverted FI matrix (non-diagonal terms included), which makes it a convenient metric with which to optimize the cell parameters. Therefore, optimizing the CRLB makes the most sense. When adding new populations of cells and values of time intervals, the CRLB can easily be adapted to the general form

26 $$CRL{B}_{{{{\rm{total}}}}}={\sum }_{k=1}^{n}\frac{{q}_{k}}{\sqrt{{\sum }_{j = 1}^{N}{p}^{j}{{{\mathcal{I}}}}({T}_{k}^{* },{\tau }^{j})}},$$

where $\mathop{\sum }_{k = 1}^{n}{q}_{k}=\mathop{\sum }_{j = 1}^{N}{p}^{j}=1$. The weights *q*_*k*_ allow the assignment of importance to specific values of time intervals, while the weights *p*^*j*^ represent the proportion of cells with time recovery parameter *τ*^*j*^ in the network of PG cells. The continuous limit of this equation was used in the optimization of the parameters (see Eq. (12)). The integral was computed numerically with an adaptive Gauss-Kronrod integration technique from the *QuadGK.jl* library and was then optimized with the L-BFGS method from the *Optim.jl* library while keeping the value of the average *τ* between 0.1 s and 80 s (box-constrained).

### Computing the determinant for a sequence of 2 time intervals

The FI matrix needs to be invertible to have a finite lower bound on the error made on the estimates of time intervals. For two populations of sizes *N*^1^ and *N*^2^, the determinant of the FI matrix is given by

27 $${{{\rm{det}}}}{{{\mathcal{I}}}} = 	 \left[\frac{{N}^{1}}{{\lambda }_{2}^{1}}{\left(\frac{\partial {\lambda }_{2}^{1}}{\partial {T}_{1}}\right)}^{2}+\frac{{N}^{2}}{{\lambda }_{2}^{2}}{\left(\frac{\partial {\lambda }_{2}^{2}}{\partial {T}_{1}}\right)}^{2}\right]\\ 	 \times \left[\frac{{N}^{1}}{{\lambda }_{2}^{1}}{\left(\frac{\partial {\lambda }_{2}^{1}}{\partial {T}_{2}}\right)}^{2}+\frac{{N}^{2}}{{\lambda }_{2}^{2}}{\left(\frac{\partial {\lambda }_{2}^{2}}{\partial {T}_{2}}\right)}^{2}\right]\\ 	 -\left[\frac{{N}^{1}}{{\lambda }_{2}^{1}}\left(\frac{\partial {\lambda }_{2}^{1}}{\partial {T}_{1}}\right)\left(\frac{\partial {\lambda }_{2}^{1}}{\partial {T}_{2}}\right)\right.\\ 	 {\left.+\frac{{N}^{2}}{{\lambda }_{2}^{2}}\left(\frac{\partial {\lambda }_{2}^{2}}{\partial {T}_{1}}\right)\left(\frac{\partial {\lambda }_{2}^{2}}{\partial {T}_{2}}\right)\right]}^{2}.$$

Expanding the multiplication of the terms in brackets yields

28 $${{{\rm{det}}}}{{{\mathcal{I}}}} = 	 \frac{{({N}^{1})}^{2}}{{({\lambda }_{2}^{1})}^{2}}{\left(\frac{\partial {\lambda }_{2}^{1}}{\partial {T}_{1}}\right)}^{2}{\left(\frac{\partial {\lambda }_{2}^{1}}{\partial {T}_{2}}\right)}^{2}+\frac{{N}^{1}{N}^{2}}{{\lambda }_{2}^{1}{\lambda }_{2}^{2}}{\left(\frac{\partial {\lambda }_{2}^{2}}{\partial {T}_{1}}\right)}^{2}{\left(\frac{\partial {\lambda }_{2}^{1}}{\partial {T}_{2}}\right)}^{2}\\ 	 +\frac{{({N}^{2})}^{2}}{{({\lambda }_{2}^{2})}^{2}}{\left(\frac{\partial {\lambda }_{2}^{2}}{\partial {T}_{1}}\right)}^{2}{\left(\frac{\partial {\lambda }_{2}^{2}}{\partial {T}_{2}}\right)}^{2}+\frac{{N}^{1}{N}^{2}}{{\lambda }_{2}^{1}{\lambda }_{2}^{2}}{\left(\frac{\partial {\lambda }_{2}^{1}}{\partial {T}_{1}}\right)}^{2}{\left(\frac{\partial {\lambda }_{2}^{2}}{\partial {T}_{2}}\right)}^{2}\\ 	 -\frac{{({N}^{1})}^{2}}{{({\lambda }_{2}^{1})}^{2}}{\left(\frac{\partial {\lambda }_{2}^{1}}{\partial {T}_{1}}\right)}^{2}{\left(\frac{\partial {\lambda }_{2}^{1}}{\partial {T}_{2}}\right)}^{2}-\frac{{({N}^{2})}^{2}}{{({\lambda }_{2}^{2})}^{2}}{\left(\frac{\partial {\lambda }_{2}^{2}}{\partial {T}_{1}}\right)}^{2}{\left(\frac{\partial {\lambda }_{2}^{2}}{\partial {T}_{2}}\right)}^{2}\\ 	-2\frac{{N}^{1}{N}^{2}}{{\lambda }_{2}^{1}{\lambda }_{2}^{2}}\left(\frac{\partial {\lambda }_{2}^{1}}{\partial {T}_{1}}\right)\left(\frac{\partial {\lambda }_{2}^{1}}{\partial {T}_{2}}\right)\left(\frac{\partial {\lambda }_{2}^{2}}{\partial {T}_{1}}\right)\left(\frac{\partial {\lambda }_{2}^{2}}{\partial {T}_{2}}\right).$$

Many terms can be canceled and putting ${N}^{1}{N}^{2}/{\lambda }_{2}^{1}{\lambda }_{2}^{2}$ in front of the expression and splitting the last term of Eq. (28) in two, we obtain

29 $${{{\rm{det}}}}{{{\mathcal{I}}}} = 	 \frac{{N}^{1}{N}^{2}}{{\lambda }_{2}^{1}{\lambda }_{2}^{2}}\left[{\left(\frac{\partial {\lambda }_{2}^{2}}{\partial {T}_{1}}\right)}^{2}{\left(\frac{\partial {\lambda }_{2}^{1}}{\partial {T}_{2}}\right)}^{2}\right.\\ 	 -\left(\frac{\partial {\lambda }_{2}^{1}}{\partial {T}_{1}}\right)\left(\frac{\partial {\lambda }_{2}^{1}}{\partial {T}_{2}}\right)\left(\frac{\partial {\lambda }_{2}^{2}}{\partial {T}_{1}}\right)\left(\frac{\partial {\lambda }_{2}^{2}}{\partial {T}_{2}}\right)\\ 	 +{\left(\frac{\partial {\lambda }_{2}^{1}}{\partial {T}_{1}}\right)}^{2}{\left(\frac{\partial {\lambda }_{2}^{2}}{\partial {T}_{2}}\right)}^{2}\\ 	 \left.-\left(\frac{\partial {\lambda }_{2}^{1}}{\partial {T}_{1}}\right)\left(\frac{\partial {\lambda }_{2}^{1}}{\partial {T}_{2}}\right)\left(\frac{\partial {\lambda }_{2}^{2}}{\partial {T}_{1}}\right)\left(\frac{\partial {\lambda }_{2}^{2}}{\partial {T}_{2}}\right)\right].$$

Then, we factor one of the squares to get

30 $${{{\rm{det}}}}{{{\mathcal{I}}}} = 	 \frac{{N}^{1}{N}^{2}}{{\lambda }_{2}^{1}{\lambda }_{2}^{2}}\left[\left(\frac{\partial {\lambda }_{2}^{2}}{\partial {T}_{1}}\right)\left(\frac{\partial {\lambda }_{2}^{1}}{\partial {T}_{2}}\right)\left\{\left(\frac{\partial {\lambda }_{2}^{2}}{\partial {T}_{1}}\right)\left(\frac{\partial {\lambda }_{2}^{1}}{\partial {T}_{2}}\right)-\left(\frac{\partial {\lambda }_{2}^{1}}{\partial {T}_{1}}\right)\left(\frac{\partial {\lambda }_{2}^{2}}{\partial {T}_{2}}\right)\right\}\right.\\ 	 \left.+\left(\frac{\partial {\lambda }_{2}^{1}}{\partial {T}_{1}}\right)\left(\frac{\partial {\lambda }_{2}^{2}}{\partial {T}_{2}}\right)\left\{\left(\frac{\partial {\lambda }_{2}^{1}}{\partial {T}_{1}}\right)\left(\frac{\partial {\lambda }_{2}^{2}}{\partial {T}_{2}}\right)-\left(\frac{\partial {\lambda }_{2}^{2}}{\partial {T}_{1}}\right)\left(\frac{\partial {\lambda }_{2}^{1}}{\partial {T}_{2}}\right)\right\}\right].$$

Finally, we note that the terms in the curly brackets can be factored out to retrieve the result shown in Eq. (14). This shows the necessity of at least two types of cell – i.e. of parametric heterogeneity – to encode two successive time intervals.

### Multilayer perceptron and reservoir computer for time interval estimation

The MLP consists of an input layer of 1000 units, a first hidden layer of 125 units followed by a second one of 1000 units, and an output layer formed of two units that retrieve *T*_1_ and *T*_2_ ("previous" and “last" time intervals, respectively). The hidden layers have a rectified linear transformation (ReLU) applied to them. The input consists of the values of the responses of the simulated adaptive PG cells during each encounter, identical to what was used for the MLE. The structure was meant to be an autoencoder, though we have not seen a large improvement over other MLP configurations we have tested. The MLP was built and trained using *Lux.jl* with stochastic gradient descent. For each composition of adaptive input, an MLP was instantiated with randomly chosen weights and trained in a supervised manner on 5000 random pairs of time intervals uniformly distributed between 0.1 s and 30 s for 3000 epochs. In other words, 5000 random sequences (*T*_1_, *T*_2_) were generated along with the corresponding responses of all cells $({R}_{2}^{1},{R}_{2}^{2},...,{R}_{2}^{1000})$. These responses were used as input to the MLP to estimate *T*_1_ and *T*_2_ and the mean square error was used as the loss function to propagate the gradient for learning. This was repeated 3000 times with the same set of 5000 pairs of time intervals. The RMSE was then calculated on a new set of sequences of 1000 pairs of time intervals to generate Fig. 10a,b. For the adaptive responses fed to the MLP, all of the simulated PG cells had *a* = 10, *c* = 0, *τ* = 15.55 s for the memory-less cells (*β* = 0) and *τ* = 30 s for the memory cells (*β* > 0).

As mentioned previously, the RC is based off the activity of an RNN. The state *s*_*i*_ of a neuron *i* of this RNN is given by

31 $${\tau }_{R}\frac{{{{\rm{d}}}}{s}_{i}}{{{{\rm{d}}}}t}=\mathop{\sum }_{j=1}^{{N}_{R}}{W}_{ij}\tanh \left({s}_{j}+{\sum }_{k=1}^{N}{J}_{ik}{I}_{k}(t)\right)-{s}_{i},$$

where *τ*_*R*_ = 5 s is the time constant of the neurons in the RC, *N*_*R*_ = 250 is the number of neurons in the reservoir, *W*_*ij*_ is the weight linking neuron *j* to neuron *i* and *I*_*i*_(*t*) is the external input to neuron *i* of the RNN at time *t*.

Since the dynamical system given by Eq. (31) is now continuous in time, we needed to convert the event-based adaptive response process used for the previous estimators to a time-continuous equivalent. To do so, we used an exponentially decaying signal whose value is increased on each encounter:

32 $${\tau }_{{{{\rm{in}}}}}\frac{{{{\rm{d}}}}I}{{{{\rm{d}}}}t}=-I+{\sum }_{{t}_{k}}{R}_{k}\delta (t-{t}_{k}),$$

where *I*(*t*) is the signal of a PG cell at time *t*, *τ*_in_ = 0.1 s is the input layer decay rate and *δ*(*x*) is the Dirac delta. During an event at time *t*_*k*_, the value of *I*(*t*) is instantly increased by an amount *R*_*k*_, corresponding to the adaptive response of the PG cell as defined previously in Eq. (3).

A neuron *i* in the RNN then combines these responses into the input ${\sum }_{k=1}^{N}{J}_{ik}{I}_{k}(t)$. The input coupling matrix *J* of size *N*_*R*_ × *N* is block-diagonal such that memory cells drive a different subset of neurons in the RNN from memory-less cells. Values of the elements of the two blocks are taken from a uniform distribution between 0 and the scale factor *S* = 0.1 and divided by the square-root of the number of elements in each block: $\sqrt{\lfloor {p}_{m}N\rfloor \lfloor {p}_{m}{N}_{R}\rfloor }$ for the memory block and $\sqrt{\lfloor (1-{p}_{m})N\rfloor \lfloor (1-{p}_{m}){N}_{R}\rfloor }$ for the memory-less block.

The parameters of the adaptive response are the same as in the MLP method, i.e., *a* = 10, *c* = 0, *τ* = 15.55 s for the memory-less cells (*β* = 0) and *τ* = 30 s for the memory cells (*β* > 0).

The weights *W* were initialized using a normal distribution centered at 0 with variance 1. We made sure the reservoir was close to the chaotic regime by dividing the weight matrix *W* by its largest eigenvalue and then multiplying it by a spectral radius of *λ*_spectral radius_ = 1.05, leading to chaos. Moreover, we randomly disconnected 10% of neurons in the RNN such that the resulting connectivity is not all-to-all as a means of regularization.

For each value of (*β*_*m*_, *p*_*m*_), an RNN was driven by a continuous sequence of 750 time intervals during training. We also added a transient of 5 time intervals before the training sequence started. This was done to make sure the RNN settles out of transient behaviors and to minimize the effect of the initial values of the adaptive processes in the PG populations. The resulting activity was computed by integrating Eq. (31) with Euler’s method using an integration step of d*t* = 0.01 s and then saved. The activity was then separated into intervals between events. For each interval, we computed an exponentially weighted sum of the activity to get a pooled value for each neuron in the RNN. For example, between events *t*_*n*−1_ and *t*_*n*_ (i.e. during interval *T*_*n*_), we get the pooled activity for neuron *i*:

33 $${Y}_{i}^{n}=\mathop{\sum }_{j=1}^{\lfloor \frac{{T}_{n}}{{{{\rm{d}}}}t}\rfloor }\tanh \left({s}_{i}({t}_{n-1}+(\,j-1){{{\rm{d}}}}t)\right){e}^{(\,j-1){{{\rm{d}}}}t/\gamma }$$

where *γ* = 0.8 s is the decay rate of the exponential pool. The assumption is that this pooled activity encodes the time intervals *T*_*n*−1_ (latest interval) and *T*_*n*−2_ (previous interval). Ridge regression^60^ was then performed on the pooled activity of all neurons of the RNN during each interval. The resulting output weights *W*_out_ were then used to estimate the last two time intervals during a new testing sequence of 250 time intervals. This new sequence was appended to the previous 750 time intervals presented during training, i.e., we kept the states of the RNN and PG cells right after training to continue the estimation process. The RMSE was computed on the output *W*_out_*Y*^*n*^ during testing and was used to generate Fig. 10c,d. See Supplementary Figs. 5 and 6 for a graphical representation of the estimation process.

The time constants *τ*_*R*_, *τ*_in_ and input scale *S* were calibrated with *β*_*m*_ = 1 and *p*_*m*_ = 1. To do so, we computed the RMSE on the previous interval of the RC trained on a continuous sequence of 750 time intervals as previously described for different combinations of *τ*_in_, *τ*_*R*_ and *S*. Specifically, we tried all combinations of *τ*_in_ ∈ {0.1, 0.5, 1.0}, *τ*_*R*_ ∈ {0.5, 1, 2, 5, 10} and *S* ∈ {0.01, 0.1, 1} as shown in Supplementary Fig. 3. We found that the RMSE seems minimal when the ratio *τ*_in_/*τ*_*R*_ is between 0.1 and 0.3 with a scale of *S* = 0.1. Moreover, the configuration that gave the lowest error among all configuration was when *τ*_in_ = 1 s, *τ*_*R*_ = 5 s and *S* = 0.1. This set of parameters was used for the “augmented" RC in Supplementary Fig. 4, while the more “limited" (but still adequate) set of parameters of *τ*_in_ = 0.1 s, *τ*_*R*_ = 1 s and *S* = 0.1 was used when comparing to the MLP in Fig. 10. Since the input signal is independent of time elapsed between events (*β*_*m*_ = 1 and *p*_*m*_ = 1), calibrating the RNN parameters for this type of input makes it a great baseline to compare against when later injecting more information about intervals through adaptation.

## Supplementary information

Transparent Peer Review file

Supplementary Information

## Data Availability

The data that was generated for all the figures of this study is available in a Zenodo repository: https://doi.org/10.5281/zenodo.17479414.

## Code availability

All computer code necessary to generate and plot data is available on Github: https://github.com/raphlaf/InferringTimeSequences.git.
