# Supplementary material for: Neural heterogeneity enables adaptive encoding of time sequences
Source: Commun Phys. 2025 Nov 24;8(1):504. doi: 10.1038/s42005-025-02408-3 (PMC12805876; doi:10.1038/s42005-025-02408-3)
Supplement: Supplementary file 1 [file 42005_2025_2408_MOESM1_ESM.pdf]

nature portfolio

Peer Review File

Neural heterogeneity enables adaptive encoding of time sequences

Corresponding Author: Mr Raphaël Lafond-Mercier

This file contains all reviewer reports in order by version, followed by all author rebuttals in order by version.

Version 0:

Reviewer comments:

Reviewer #1

(Remarks to the Author)
The authors investigate theoretically the possibility of encoding time intervals between events by means of history-dependent adaptation of single-neuron responses. The proposal is motivated by experimental observations in weakly-electric fishes, as discussed in the manuscript. It is worth noting that a proposal conceptually very similar to the present one can be found in Hopfield (1996).

The manuscript addresses an important problem as 'encoding of time' in the brain remains an outstanding major question.

The main result is "a proof that heterogeneity is needed to encode two or more time intervals" (lines 1204-1205) with this mechanism. It seems to me that, without further qualifications, this claim is incorrect. This is easy to see.

Let us consider a single neuron without memory and let us try to estimate two intervals, T1 and T2, from its responses, R1 and R2. The Fisher information matrix (FIM) of a single neuron is the same as the FIM of a homogeneous population of (independent) neurons, apart from an immaterial prefactor. The two responses are statistically independent (because there is no memory), and hence the log-likelihood, LL(T1,T2; R1,R2), is simply the sum of the log-likelihood of the two responses, LL(T1,T2; R1,R2)=LL(T1;R1)+LL(T2;R2). The FIM is well defined and invertible; in fact, it is diagonal and the elements on the diagonal are the Fisher information for the single intervals (which are all different from zero, or a set of parameters can be found so that they are all different from zero). Obviously, the same argument can be extended to a neuron with memory.

Minor:

In the model, the depletion of x is independent of the actual response of the neuron (i.e., the number of spikes fired). It is hard to imagine how this could happen at the biophysical level. More importantly, it is unclear how the introduction of this biophysical constraint will affect the performances of the proposed mechanism.

It is never said that the parameter \beta is between 0 and 1.

The 'firing parameter' \lambda is simply the average number of spikes fired during an encounter. Increasing a is the same as increasing the 'observation time', while increasing c is the same as increasing 'noise'.

On lines 239-240, the authors promise that "parentheses will be used to differentiate indices from exponents". Indeed, this is never done.

Reviewer #2

(Remarks to the Author)

Lanfond-Mercier and colleagues aim to put forward a general theory as to how neural adaptation encodes the time elapsed between identical stimuli. This work is inspired from recent findings that fish preglomerular complex (PG) cells produce bursts of firings in response to the fish encountering an object, whereby the number of spikes in the burst depend on the time taken since the previous encounter. Specifically, PG cells have heterogeneity in their sensitivity to historical encounters and the authors aim to determine if this heterogeneity is optimal. They purport to prove that this heterogeneity is required.

The authors do well to outline when homogeneous time constants would be optimal (estimating single interval estimation) and when heterogeneous time constants would be optimal (estimating multiple time intervals).

The insight that for the determinant of the FI matrix to be non-zero, there needs to be some degree of heterogeneity to encode two or more intervals in addition to at least one cell type that can encode the first-time interval in the sequence, is impressive. The work is theoretically sound, and I am impressed with the overall work.
I would like the authors to deal with the following points. These can largely be summarised as ensuring that the claim rather than being that "heterogeneity is necessary to encode temporal sequences" it is not "Under ideal conditions with perfect MLE decoding and this specific model, heterogeneity is necessary to achieve optimal parameter estimation for 2 intervals":

• To what extent their findings generalise to N intervals or M populations.
• Real temporal sequences involve many more intervals, how does their findings scale with many intervals.
• To what extent their conclusions depend on the specific resource depletion equations.
• Under what other adaptation assumptions would this not hold?
• "Recovering the sequence of time intervals from the responses of PG cells can be achieved through maximum likelihood estimation (MLE), which is an efficient estimator given enough cells." Can you justify this further?
• How does your model deal with noise? I would like to see interval estimates under noisy conditions.

We now can train spiking neural networks with learnable time constants to be able to solve tasks (e.g., Perez-Neives, et al. 2021 Nature Communications). To strengthen the theoretical findings, I recommend the authors comprehensively validate their work. One experiment would implement the resource-based adaptation model in trainable SNNs, creating both homogeneous and heterogeneous networks with exact parameter distributions to test core predictions under realistic conditions: specifically, that homogeneous networks should excel at single-interval tasks while heterogeneous networks should outperform on multi-interval sequences, unlike the analytical approach with seemingly specific assumptions. An analogous test would estimate whether heterogeneity emerges naturally by starting with homogeneous networks where relevant parameters (τ, β, gain) are identical, then training on temporal sequence reconstruction tasks using gradient descent while monitoring whether parameter diversity emerges spontaneously compared to networks where heterogeneity is explicitly prevented. If heterogeneity emerges without being explicitly designed through optimization pressure alone, this will provide strong that the theoretical predictions translate to practical advantages.

Perez-Nieves, N., Leung, V.C.H., Dragotti, P.L. et al. Neural heterogeneity promotes robust learning. Nat Commun 12, 5791 (2021). https://doi.org/10.1038/s41467-021-26022-3

Version 1:

Reviewer comments:

Reviewer #1

(Remarks to the Author)
The authors have satisfactorily addressed all my concerns with the previous version of the manuscript and clarified the points that were not completely clear to me. I think that the clarity and the quality of the manuscript has improved as a result of addressing the comments of the reviewers. The manuscript addresses an important, unsolved problem and, in doing so, provides valuable ideas.

Reviewer #2

(Remarks to the Author)
I thank the authors for their comprehensive response & I have no further comments. Well done to the authors.

Open Access This Peer Review File is licensed under a Creative Commons Attribution 4.0 International License, which permits use, sharing, adaptation, distribution and reproduction in any medium or format, as long as you give appropriate credit to the original author(s) and the source, provide a link to the Creative Commons license, and indicate if changes were made.
In cases where reviewers are anonymous, credit should be given to 'Anonymous Referee' and the source.
The images or other third party material in this Peer Review File are included in the article's Creative Commons license, unless indicated otherwise in a credit line to the material. If material is not included in the article's Creative Commons license and your intended use is not permitted by statutory regulation or exceeds the permitted use, you will need to obtain permission directly from the copyright holder.
To view a copy of this license, visit https://creativecommons.org/licenses/by/4.0/

Reviewer #1

The authors investigate theoretically the possibility of encoding time intervals between events by means of history-dependent adaptation of single-neuron responses. The proposal is motivated by experimental observations in weakly-electric fishes, as discussed in the manuscript. It is worth noting that a proposal conceptually very similar to the present one can be found in Hopfield (1996).

The manuscript addresses an important problem as 'encoding of time' in the brain remains an outstanding major question.

Thank you for taking the time to review our paper and for providing such valuable feedback and comments. They were helpful in making the manuscript clearer in terms of notation and explanation of the problem.

Thanks for this reference, which we now discuss in the paper. What Hopfield proposed is a way to encode sequences of syllables in analog variables, namely through the adaptive firing rate of neurons which should be proportional to the time elapsed since the beginning ($\tau[A_s]$) or ending ($\tau[A_e]$) of the utterance of a syllable A. The classification of words then depends on the comparison between the set of time intervals of uttered syllables $\tau_b = \{\tau[A_s], \tau[A_e], \tau[B_s], \tau[B_e], \ldots\}$ since classification time t and a known (learned) occurrence of such set with a possible time warp λ (e.g. a word that was learned when spoken more slowly) such that $\tau[K] \approx \lambda\tau_{learned}[K]$ for all symbols K. This is then compared to how olfactory systems solve a mathematically similar problem to recognize odors. Word recognition would therefore not need circuitry that much different from olfaction.

The method for encoding such time-dependent sequences is indeed relevant to the present paper. The adaptation to encode time is similar to our model, although there are some key differences. The first one being that a cell is said to be specialized to encode the time since a specific syllable onset and will not be active if another syllable is uttered. This would be equivalent to some PG cells responding to a specific landmark in a sequence rather than all of them, which is not what was observed experimentally.

Second, regarding the specific adaptation process put forward, it is quite different to what is analyzed in this paper. Indeed, we are looking at the adaptive *intensity* of short bursts happening during an encounter, while Hopfield's model looks at the adaptation of the firing rate over time since the syllable utterance onset. However, he does mention without going into much detail different possible types of sequence encoders, bursting neurons being one of them.

Finally, the use of adaptation in Hopfield's paper is more about classifying sequences into words in spite of timing differences, rather than using those sequences to encode something directly related to the time spent on a syllable. Our model specifically looks at the time spent between encounters to relate it directly to distances and build a mental map of the surroundings.

Interestingly, however, there may be a higher-level processing of such sequences that can relate to what Hopfield proposed. For example, this maybe be relevant if the fish revisits its surroundings at a faster pace than before. In that case, the responses of PG cells would help

with the time warp incurred and help successfully classify such sequences of time intervals as a previously learned path. Our work therefore provides a thorough analysis of the level of confidence that can be achieved in such a context. Performance in interval estimation should be directly related to the performance of path classification. The description of the Hopfield paper, its connection to our work and its potential use for path learning is now mentioned in the DISCUSSION of the revised manuscript at lines 1841-1873.

The main result is "a proof that heterogeneity is needed to encode two or more time intervals" (lines 1204-1205) with this mechanism. It seems to me that, without further qualifications, this claim is incorrect. This is easy to see.

Let us consider a single neuron without memory and let us try to estimate two intervals, T1 and T2, from its responses, R1 and R2. The Fisher information matrix (FIM) of a single neuron is the same as the FIM of a homogeneous population of (independent) neurons, apart from an immaterial prefactor. The two responses are statistically independent (because there is no memory), and hence the log-likelihood, LL(T1,T2; R1,R2), is simply the sum of the log-likelihood of the two responses, LL(T1,T2; R1,R2)=LL(T1;R1)+LL(T2;R2). The FIM is well defined and invertible; in fact, it is diagonal and the elements on the diagonal are the Fisher information for the single intervals (which are all different from zero, or a set of parameters can be found so that they are all different from zero). Obviously, the same argument can be extended to a neuron with memory.

The point you are making is something that we have pondered and worked on for a while now, although in another context completely. Indeed, given the fact that adaptive neurons respond to each encounter, why not use the entirety of these responses to estimate the complete sequence of time intervals? Following the paper's notation, the response of a single neuron R_1 during encounter 1 (which happens at time $t_1 = t_0 + T_1$) contains information about T_1, while its response R_2 during encounter 2 (happening at time $t_2 = t_1 + T_2$) contains information about T_2 and possibly T_1, depending on the adaptation parameters of the neuron. Therefore, as you have clearly stated, computing the maximum log-likelihood from both R_1 and R_2 shouldn't require more than one type of neuron to accurately retrieve T_1 and T_2.

However, the major consideration in this case is that the previous response R_1 must be stored somewhere else than in the adaptation mechanism to be able to retrieve it at estimation time t_2. This requires a separate model of memory, which we did not address here. That is a different problem. Instead, in the context of adaptive memory only, we explored what can be said about T_1 and T_2 only from the response R_2 available during the latest encounter event at t_2. In other words, maximizing $\ell(T_1, T_2|R_2)$. In this case, heterogeneity in the adaptation parameters is absolutely necessary to retrieve both time intervals accurately, as is shown in the paper. Thus, beyond a proof of principle that information about the prior "sequence" persists in some form in every response R, our paper quantifies what this information is, and how it is best extracted. We have made this clearer now in the Abstract and Introduction, contrasting this more difficult task to one that uses all the responses that have occurred up to a given time, with would require accompanying additional assumptions and computations. Our focus on this difficult task has enabled us to expose this unexpected necessity of heterogeneity.

Following one of Reviewer #2's comments, we now provide new analysis using other downstream estimators, one of which is a reservoir computer (RC) made of the activity of a recurrent neural network (RNN). This is relevant to the observation you have made here. Indeed, the RC could estimate two successive time intervals (the "latest" one T_2 and the "previous" one T_1) better than chance even with a homogeneous memory-less population of adaptive neurons, while a multilayer perceptron (with no external memory) could not. This is due to the recurrence of the RC, making it capable of storing information about previous responses externally from the adaptation mechanism. In other words, the RC can use previous responses (to some extent, at least) to estimate T_1 and T_2.

However, we also show that heterogeneous adaptation still has better performance when estimating the previous time interval T_1. The interaction between adaptation and this type of external memory warrants a new study altogether, especially since it heavily depends on the model of external memory used. The conclusions of the present paper are the foundations of such future elaborations of adaptive time coding.

Furthermore, as we argue in response to Reviewer 2's comments, a recurrent approach was not pursued for the encoding of interval sequences into response sequences because the structure where this is computed in the electric fish is not recurrent. Thus the question became: how does a feedforward ensemble of heterogeneous adaptive neurons encode sequences, and what are the limits on its downstream decoding? That is the problem we set out to answer. Again, we have clarified this point further in our resubmission.

With respect to your last sentence: "Obviously, the same argument can be extended to a neuron with memory.": Even in the context where all responses are available, this would not be true because of the fact that the FI matrix is diagonal only in the case of memory-less cells. And when using ONLY the last response to decode the sequence, only the last element I_{22} is non-zero. However, memory cells render the FI matrix non-diagonal. This can easily be seen when looking at Equation (16). If we were to use both responses for a single cell, we get the off-diagonal element:

$$\frac{1}{\lambda_1}\frac{\partial\lambda_1}{\partial T_1}\frac{\partial\lambda_1}{\partial T_2}+\frac{1}{\lambda_2}\frac{\partial\lambda_2}{\partial T_1}\frac{\partial\lambda_2}{\partial T_2}.$$

The first term goes to 0 as λ_1 cannot depend on T_2. However, the second term is not 0, as λ_2 does depend on both T_1 and T_2 since the cell has memory. Therefore, the invertibility of the matrix is not as obvious as in the memory-less case.

Minor:

In the model, the depletion of x is independent of the actual response of the neuron (i.e., the number of spikes fired). It is hard to imagine how this could happen at the biophysical level. More importantly, it is unclear how the introduction of this biophysical constraint will affect the performances of the proposed mechanism.

Since we do not know exactly where the adaptation with such time constants comes from, we assume that the resource-based dynamics is the result of pre-synaptic activity. Indeed, we suspect that it is not a functional property of a PG cell itself, but rather a result of interactions between multiple brain regions in the fish. Although, as we now also clarify in the manuscript, we can not exclude contributions from long-lived intrinsic currents in the PG cells. We added the following to the RESULTS section regarding this point (lines 265-280):

"It is important to note that the specific response value R_n of one cell does not directly influence future responses R_{n+1}, R_{n+2},... Specifically, they are conditionally uncorrelated, but become dependent upon marginalizing over the prior of intervals through the memory in the x-process. Indeed, we assume that the resource-based dynamics is a result of pre-synaptic activity, although we can not exclude contributions from intrinsic currents in PG cells. It is also possible that other nuclei downstream from PG – such as the pallium – further influence adaptation time scales through e.g. feedback loops [43]. Therefore, the statistics of all PG neurons dictate future responses, which is what is modeled here."

It is never said that the parameter \beta is between 0 and 1.

Fixed in the manuscript.

The 'firing parameter' \lambda is simply the average number of spikes fired during an encounter. Increasing a is the same as increasing the 'observation time', while increasing c is the same as increasing 'noise'.

In the context of a Poisson process representing the spiking response of a neuron, increasing the observation time is indeed equivalent to increasing a. In fact, this resource-based adaptation can be approached from the perspective of an inhomogeneous Poisson process, where the instantaneous firing rate changes with time (see Tsodyks & Markram 1997). To make the conversion to an event-based representation, we implicitly chose a fixed time window during which we count the number of spikes happening. Then, since this time window is kept fixed for all cells, increasing a is the same as increasing the overall firing rate during the encounter. In other words, some cells may have a higher sensitivity to available resources than others, which is why we called it the gain of the cell. This is now mentioned in the revised manuscript following your query (lines 241-246).

The baseline activity $c > 0$ could indeed be called noise, as it does not relate to adaptation activity. However, when $c < 0$, this appellation is not as appropriate. Indeed, it indicates the need for a stronger stimulus (longer interval) before the cells are activated. There were some neurons found to be silent like that, but we did not explore their coding capacity. This is the main reason we did not call c noise, though we now mention it in passing in the revised manuscript (lines 246-250).

On lines 239-240, the authors promise that "parentheses will be used to differentiate indices from exponents". Indeed, this is never done.

The placement of this notice might not have been ideal. We changed it so it is said right after superscripts are used to denote neurons for the first time in Equation (4). We also changed the wording to make things a bit clearer. Lines 309-318 in the revised manuscript now read:

“When treating multiple neurons or populations, the superscript will be the index of an adaptive neuron or population, while the subscript will be the index of an encounter. Parentheses will be used to differentiate indices from exponents when necessary. However, to lighten up the notation, expressions with only one set of neuron parameters (i.e. single homogeneous population) will not have this differentiation; superscripts will be considered as exponents.”

Hopefully this makes the notation used throughout the manuscript clearer (e.g. the y-axis of Figure 3D-F is not τ squared, while terms in parentheses in Equation (28) are squared).

Reviewer #2:

Lanfond-Mercier and colleagues aim to put forward a general theory as to how neural adaptation encodes the time elapsed between identical stimuli. This work is inspired from recent findings that fish preglomerular complex (PG) cells produce bursts of firings in response to the fish encountering an object, whereby the number of spikes in the burst depend on the time taken since the previous encounter. Specifically, PG cells have heterogeneity in their sensitivity to historical encounters and the authors aim to determine if this heterogeneity is optimal. They purport to prove that this heterogeneity is required.

The authors do well to outline when homogeneous time constants would be optimal (estimating single interval estimation) and when heterogeneous time constants would be optimal (estimating multiple time intervals).

The insight that for the determinant of the FI matrix to be non-zero, there needs to be some degree of heterogeneity to encode two or more intervals in addition to at least one cell type that can encode the first-time interval in the sequence, is impressive. The work is theoretically sound, and I am impressed with the overall work.

I would like the authors to deal with the following points. These can largely be summarised as ensuring that the claim rather than being that “heterogeneity is necessary to encode temporal sequences” it is not "Under ideal conditions with perfect MLE decoding and this specific model, heterogeneity is necessary to achieve optimal parameter estimation for 2 intervals":

• To what extent their findings generalise to N intervals or M populations.

• Real temporal sequences involve many more intervals, how does their findings scale with many intervals.

Thank you for your time and insightful feedback regarding our manuscript. It made us explore a new direction and allowed us to present a more extensive analysis of multiple interval encoding.

We will discuss your first two points together here. A generalization of the results shown in Equation (14) to n intervals and N neurons or populations of identical neurons would be ideal. Despite our efforts, this could not be done analytically. The main takeaway from our attempts is that it is not obvious what constraints a non-zero determinant puts on the adaptation parameters for more than 2 time intervals. However, we propose a numerical analysis of the situation to show how the conclusion seems to be identical for larger sequences, i.e. there needs to be at least the same number of different populations as the length of the sequence to estimate ($N \geq n$).

We have added the following in the RESULTS section (lines 1243-1456):

"The idea of the necessity of heterogeneity can also be generalized to a sequence of more than two time intervals and populations. Here, we present a numerical argument, because this problem is currently analytically intractable. Since this was done numerically, we needed to choose a specific sequence of time intervals for which to compute the determinant of the FI matrix. To make things simple, we chose a sequence of repeated intervals of 5 s, and proceeded systematically from single to multiple intervals and populations. We started with a "sequence" of 1 interval and an estimator with 1 population. We changed the memory parameter and the recovery time for this population and computed the FI determinant. The maximum determinant is reached when $\beta_1 = 0$ and $\tau_1 \approx 7.84$ s (see Figure 8A), which is what is expected from the theory. We then added another interval to the sequence and a new population to the estimator. The parameters of the first population were set to the maximum that was previously found for the case of a single time interval. We computed the determinant while changing the values of the parameters of the added population. The determinant is always 0 when $\beta_1 = \beta_2 = 0$, and there is a maximum when $\beta_2 \approx 0.42$ and $\tau_2 \approx 13.2$ s (see Figure 8B).

We repeated the process by adding a third interval to be estimated as well as a third population. This time, there are two lines in the space (β_3, τ_3) for which the determinant is 0 (see Figure 8C). Those lines pass through $(\beta_1 = 0, \tau_1 = 7.84)$ and$(\beta_2 = 0.424, \tau_2 = 13.2)$, showing that a new population identical to either previous ones cannot produce a valid estimator for 3 time intervals. There are also other populations whose parameters are completely different from either previous populations, but still lead to a non-invertible FI matrix. In other words, these combinations of unique populations will still lead to a degeneracy in the estimation of (T_1, T_2, T_3). In that case, an equivalent observation is that the CRLB diverges to infinity, indicating that difference values of time intervals are equally likely. This phenomenon can be observed for longer sequences as well (4 to 6 time intervals shown in Figure 8D to F, respectively). The choice of population parameters therefore becomes increasingly difficult as the length of the sequence increases, due to the addition of new "invalid lines" in (β, τ)-space (dashed lines in Figure 8). We also expect this pairwise restriction on the populations to become more complex as the variability in sequences changes (e.g. when they are not simply repeated identical intervals).

The values of the different maxima and their associated parameters are shown in Table 1. When studying the problem for one or two intervals, we could visualize the performance of an

estimator through the error of the individual elements of the sequence (T_1 and/or T_2). When increasing the dimensionality of the problem, however, this becomes impossible. We therefore calculated the CRLB on the sum of the time intervals in the sequence for each maximum of Figure 8. To do so, we recall that, assuming no bias, the covariance matrix is bounded by $cov[T_{ij}] \geq [I^{-1}]_{ij}$. Since the variance for the sum $Z_n = T_1 + T_2 + \cdots + T_n$ is $\sum_{i,j=1}^{n} cov[T_{ij}]$, we get the CRLB on the sum of intervals with

$$CRLB_{Z_n} \geq \sqrt{\sum_{i,j=1}^{n} [I^{-1}]_{ij}}.$$

This is a useful metric to treat as a performance indicator for sequences of arbitrary length. Even when using population parameters that maximize the FI determinant, we see that the value of $CRLB_{Z_n}$ increases exponentially (see also Figure S3), which should be taken as a sign that estimates become increasingly hard to generate as sequences grow in size, which is not surprising.

As previously mentioned, the degeneracy phenomenon is dependent on the local nature of the FI. A combination of populations that cannot estimate a specific sequence of time intervals may still be able to lift the degeneracy for another sequence. For example, two populations with different memory parameters ($\beta_1 = 0.4, \beta_2 = 0.7$) and time constants ($\tau_1 = 15$ s, $\tau_2 = 8.2$ s) will make any sequence of two time intervals beginning with $T_1 = 10$ s impossible to estimate accurately, while still performing adequately for other sequences (see Figure 9C). Note that this is simply by construction. We chose values of β_1,β_2 and τ_1, then calculated the value of τ_2 that would give a zero determinant when $T_1 = 10$ s using Equation (14) to illustrate the problem.

However, having a memory-less population combined with a memory population ($\beta_1 = 0, \beta_2 = 0.4$) with identical time constants ($\tau_1 = \tau_2 = 15\ s$) completely eliminates this degeneracy (see Figure 9A), as expected. When two populations have the same non-zero memory parameter ($\beta_1 = \beta_2 = 0.4$) and different time constants ($\tau_1 = 15\ s, \tau_2 = 8.2\ s$), the degeneracy is also lifted (see Figure 9B). In other words, the degeneracy problem and thus the decoding problem can easily be circumvented for a sequence of two time intervals.

Increasing the number of time intervals to 3 makes it more difficult to remove the degeneracy, because there is no other choice than to have at least 2 populations with different non-zero memory parameters. In this case, a combination of parameters that are not carefully selected makes it impossible to estimate some sequences. Even when all parameters are different (e.g. $\beta_1 = 0.2, \beta_2 = 0.4, \beta_3 = 0.7, \tau_1 = 25.6\ s, \tau_2 = 15\ s, \tau_3 = 8.2\ s$), the responses of those populations lead to different time interval sequences to have the same likelihood just like in the homogeneous case (see Figure 9D-F). It is therefore necessary to have a well selected heterogeneity in the adaptation parameters to cover a wide range of sequences."

The following was also added to the DISCUSSION section (lines 2033-2083):

“We remind the reader of the relation between the magnitude of the determinant of the FI matrix and the CRLB. This was done in order to better appreciate why conditions leading to a null determinant correspond to degeneracy (the problem is not uniquely invertible). Furthermore, they remind us of the difference between a null determinant and one that is close to zero; the former formally implies that intervals can’t be uniquely decoded, while the latter suggest that it may be difficult numerically, and more so with downstream noisy neural wetware.

We made steps towards generalizing these ideas through numerical explorations, given the analytical complexity – and possibly the analytic insolubility – of the problem. By computing the determinant of the FI matrix for a sequence of repeated intervals, we were able to show that a sensible choice for the parameters of different populations is needed for the estimation problem to be invertible. This choice becomes more difficult as the number of previous time intervals to estimate in a sequence increases, as the possible values of the parameters (β, τ) of the last added population that yields a positive determinant become scarcer. We showed how an ill-selected combination of adaptation parameters makes some specific sequences of 2 or 3 time intervals impossible to differentiate from each other. We expect a similar effect to happen when increasing the length of the estimated sequence, making it more difficult to find a network of adaptive neurons capable to estimate all time intervals – and combinations thereof – of interest.

One way to solve this would be to increase the number of different populations for the same sequence length. More heterogeneity helps reduce the degeneracy, as a sub-combination of populations incapable of estimating some sequence can be helped by a new population whose degeneracy lies elsewhere. This is effectively what happens in reality, as the parameters of the cells in PG are distributed continuously and not as multiple sets of populations of identical neurons. This problem is, however, quickly eclipsed by the fact that the actual error made on the estimates grows exponentially fast as the sequence to estimate increases with size, regardless of heterogeneity.”

Here are the new Figures 8 and 9 for convenience:

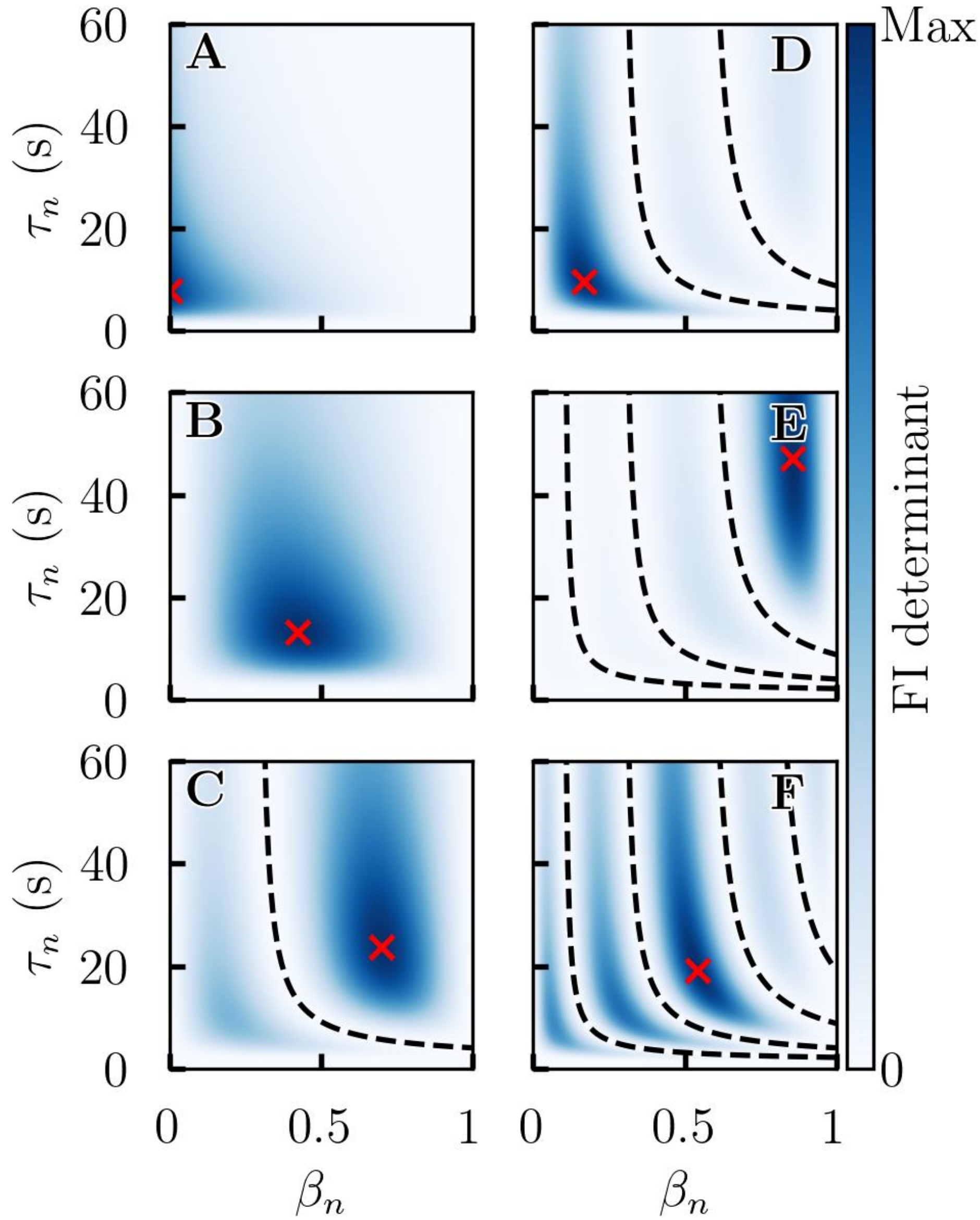

Fig. 8: More populations of neurons are necessary to make the determinant of the Fisher information matrix non-zero as sequence increases in size. A sequence of n repeated time intervals of 5 s was used to compute the associated FI determinant with n populations. Starting with $n = 1$, the parameters of the last added population were changed (β_n and τ_n) while other populations were fixed at the maximum of previous determinant computations ($n-1, n-2, \ldots, 1$). A determinant of zero indicates that the estimation of the sequence is impossible, which becomes more difficult to avoid as the length of the sequence to estimate increases due to the increasing number of 0 determinant areas (dashed curves). (A) to (F) 1 to 6 intervals and populations, respectively. The color scheme was rescaled for each situation individually, because the units and scale of the determinant changes as the sequence gets longer. The red crosses are the locations

of maxima that were used as population parameters for longer sequences and their values are shown in Table 1. The x and y-axes are shared by all panels.

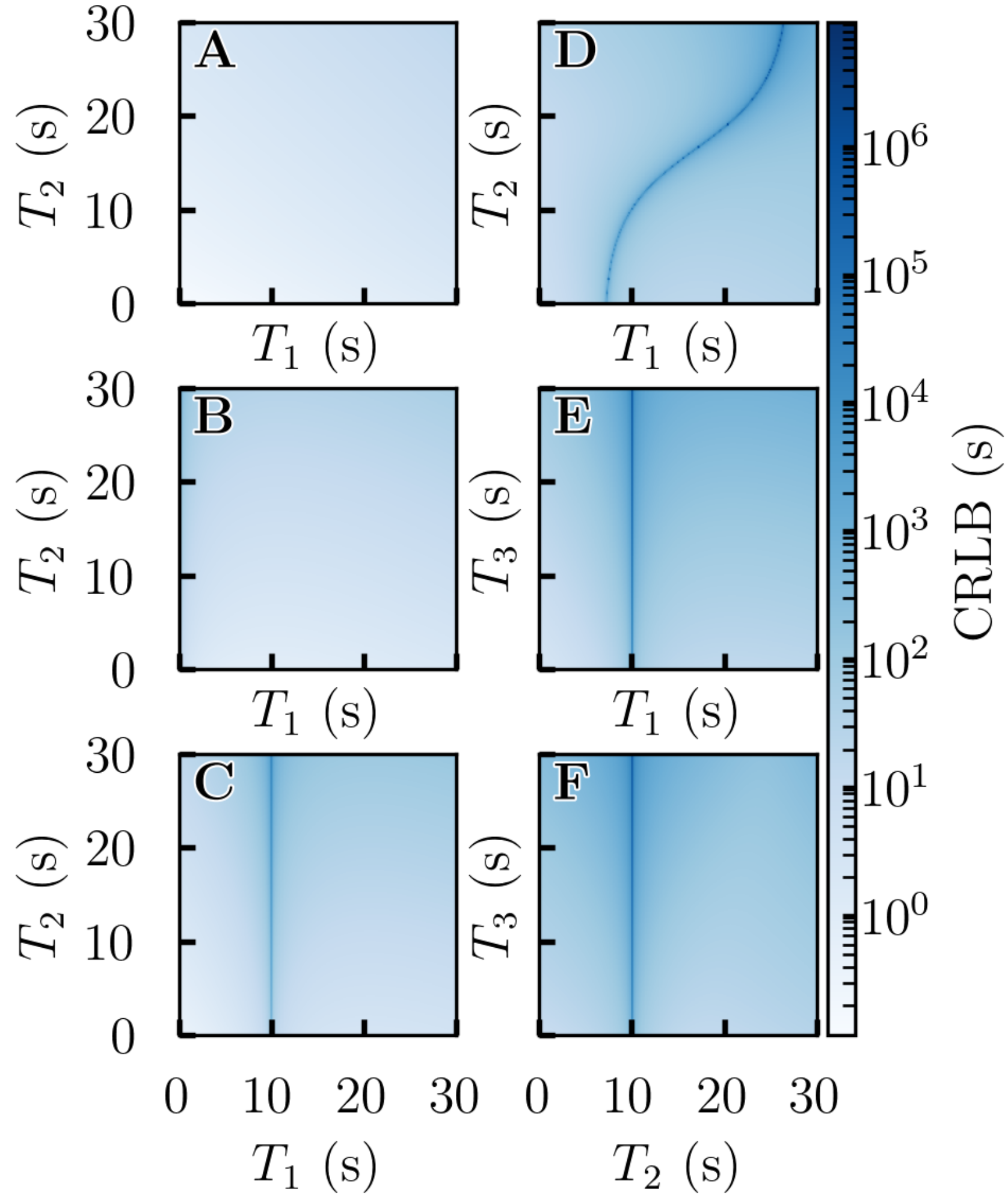

Fig. 9: A wrong selection of populations leads to sequences that are impossible to estimate. (A) to (C) Cramér-Rao lower bound (CRLB) of the sum of intervals (see Equation (15)) for a sequence of 2 time intervals and 2 populations. (A) A memory-less population combined with a memory population ($\beta_1 = 0, \beta_2 = 0.4, \tau_1 = \tau_2 = 15\ s$) yields a finite CRLB for all values of time intervals. (B) Two memory populations with different time constants ($\beta_1 = \beta_2 = 0.4, \tau_1 = 15\ s, \tau_2 = 8.2\ s$) also yields a finite CRLB for all values of time intervals. (C) Two memory populations with different memory parameters and time constants ($\beta_1 = 0.4, \beta_2 = 0.7, \tau_1 = 15\ s\ , \tau_2 = 8.2\ s$) can yield a zero determinant for some specific time interval values. Here, the parameters are such that the FI matrix is never invertible when $T_1 = 10$ s and thus leads to an infinite CRLB in that case. (D) to (F) CRLB of the sum of intervals for a sequence of 3 time intervals and 3 populations. For all three panels, the population parameters are the same ($\beta_1 = 0.2, \beta_2 = 0.4, \beta_3 = 0.7, \tau_1 =$

$25.6\ s, \tau_2 = 15\ s, \tau_3 = 8.2\ s)$. Each panel is a slice of (T_1, T_2, T_3)-space with constant $T = 10$ s for the interval not shown in the axes. Only the combination of T_1 and T_2 values affects whether the CRLB is finite. When a sequence starts with specific values of T_1 and T_2 as in (D), an accurate estimation of the sequence is impossible. In terms of numerical values, the x and y-axes are shared by all panels.

• To what extent their conclusions depend on the specific resource depletion equations.

The heterogeneity requirement does not depend on the actual resource depletion equation. The main requirement to have a non-zero determinant is when

$$\frac{\partial x_2^1}{\partial T_1}\frac{\partial x_2^2}{\partial T_2} - \frac{\partial x_2^2}{\partial T_1}\frac{\partial x_2^1}{\partial T_2} \neq 0.$$

Any resource depletion equation describing x should be ok, so long as there are enough parameters that can be changed from one population to another such that the above equation is respected.

A different way to view this requirement is to see the FI as a deterministic description of a stochastic process. In other words, it is a way to describe how the estimator behaves on average. For example, if we are given the responses of cells from 2 different populations of identical neurons after a sequence of 2 time intervals, the average response of each population will be an estimate of the firing parameters $\lambda_2^1(T_1, T_2)$ and $\lambda_2^2(T_1, T_2)$. Then, the existence of a unique solution stems only from the invertibility of the system of equations $(\lambda_2^1, \lambda_2^2) \leftrightarrow (T_1, T_2)$. The non-zero determinant of the FI matrix argument is then a restatement of the inverse function theorem, which states that a function is locally invertible if the determinant of the Jacobian of said function is non-zero. Therefore, we believe that any resource depletion equations for which the Jacobian is invertible should arrive at the same conclusions, regardless of the stochasticity around such process. This is now briefly mentioned in the revised manuscript at lines 1118-1128.

• Under what other adaptation assumptions would this not hold?

As long as the adaptation process exhibits some memory, the results presented here are expected to be generic. They may vary in their quantitative strength, such that if they are too weak w.r.t. e.g. the noise level, the adaptation with memory may not be able to provide enough support to the non-memory cells. This is so regardless of whether the adaptation is a consequence of intrinsic currents in PG cells, properties of their synapses, network properties, or a combination of all of these factors.

• "Recovering the sequence of time intervals from the responses of PG cells can be achieved through maximum likelihood estimation (MLE), which is an efficient estimator given enough cells." Can you justify this further?

An estimator is efficient when the error reaches the CRLB. MLE is always an efficient estimator given an infinite amount of sample. In our case, the error becomes close to the CRLB when the

number of adaptive neurons is large enough (around 1,000 neurons, see Figure 4). We mentioned this specification in the revised manuscript at lines 297-299.

• How does your model deal with noise? I would like to see interval estimates under noisy conditions.

The model is quite noisy already with the Poisson distribution of responses, making it almost a worse case scenario. This makes it quite difficult to differentiate between a large response due to a long interval and one due to the long-tailed nature of such distribution. Our results align with the idea that averaging over multiple responses allows for a great accuracy of the time intervals, even if individual responses are quite noisy. The variability of time intervals between encounters is also explored extensively using different types of prior distributions.

We now can train spiking neural networks with learnable time constants to be able to solve tasks (e.g., Perez-Neives, et al. 2021 Nature Communications). To strengthen the theoretical findings, I recommend the authors comprehensively validate their work. One experiment would implement the resource-based adaptation model in trainable SNNs, creating both homogeneous and heterogeneous networks with exact parameter distributions to test core predictions under realistic conditions: specifically, that homogeneous networks should excel at single-interval tasks while heterogeneous networks should outperform on multi-interval sequences, unlike the analytical approach with seemingly specific assumptions. An analogous test would estimate whether heterogeneity emerges naturally by starting with homogeneous networks where relevant parameters (τ, β, gain) are identical, then training on temporal sequence reconstruction tasks using gradient descent while monitoring whether parameter diversity emerges spontaneously compared to networks where heterogeneity is explicitly prevented. If heterogeneity emerges without being explicitly designed through optimization pressure alone, this will provide strong that the theoretical predictions translate to practical advantages.

Perez-Nieves, N., Leung, V.C.H., Dragotti, P.L. et al. Neural heterogeneity promotes robust learning. Nat Commun 12, 5791 (2021). https://doi.org/10.1038/s41467-021-26022-3

Thank you for bringing this great paper to our attention, we have now cited it. This is a relevant direction to confront the theory to other realistic contexts, which differ from the one we are addressing (see below). Further, building a trainable model of an adaptive spiking neuron and doing a rigorous analysis of such SNN in a variety of tasks (to be defined) would be a huge effort. Although we are positive this kind of architecture as explored in that paper (globally recurrent network) would benefit from adaptation, we wish to stay true to the anatomy of the fish in our analysis, as it motivated our study of this general adaptive coding problem. Indeed, PG is believed to not have local recurrent connections (Maler, unpub. anatomy). The target of PG (dorsolateral pallium, DL), however, has been shown to be a recurrent network and its connectivity has been quantified (Trinh et al. 2015). The dynamics of the DL cells has been identified (Trinh et al. 2019) and is complicated given that they have a long timescale threshold adaptation. The synaptic properties are, however, unknown.

Therefore, at this point in time, developing an SNN loosely based on realistic data would be an overwhelming and possibly ill-advised task; rather, we need to approach the general problem in stages, the first one being this manuscript. In other words, extending the problem addressed in

our manuscript to a recurrent (rather than parallel feedforward) structure WITH ADAPTIVE UNITS would be at odds with our current understanding of the circuitry. This is apart from the myriad choices we would have to make to even start modeling the encoding and decoding of interval sequences with various priors using adaptive cells that are recurrently connected (e.g. gain issues and the possible inclusion of interneurons, sparseness, plasticity, etc…). As it stands, our 2018 eLife paper where the parallel feedforward model is presented provides plausible explanations for both electrophysiological and behavioural (search accuracy) data.

Nevertheless, inspired by your suggestion, and as a first step towards this objective, we developed our paper significantly to put forward two additional estimators. Both use the adaptive response of simulated PG cells to predict past intervals in a sequence of encounters. The first is a multi-layer perceptron which allows us to go beyond the MLE estimation. The second is a recurrent network, albeit with non-adaptive units – future work will look into the complex problem of adaptive recurrent decoding. For now, we present results about how heterogeneity in the PG population upstream from these two decoders facilitates their decoding (i.e. retrieval) of interval sequences.

We have added the following to the RESULTS section (with new decoder descriptions in Methods) at lines 1544-1692:

"To make sure the necessity of heterogeneous adaptation is not just a result of the idealized context of MLE, we explored the performance of two additional estimators. The first estimator is a multilayer perceptron (MLP) and the second estimator is a reservoir computer (RC) made of a recurrent neural network (RNN). We chose such estimators to stay true to the anatomy of the brains of weakly electric fish, where the downstream structure receives independent inputs from PG. Therefore, the input to the MLP is simply the adaptive spiking responses of $N = 1{,}000$ PG cells during one encounter in a sequence, while the input to the RC is a time-continuous equivalent. Similarly to MLE, the MLP was fed the response of PG cells $\{R_n^j\}_{j=1}^N$ during encounter n and was trained in a supervised manner to predict the underlying sequence of $n = 2$ intervals. The RC was trained on a slightly different signal. A long sequence was applied to the PG cells and their subsequent responses were fed to the RC, which then had to predict the last two time intervals in the sequence at any time t during simulation (see Methods). For clarity, we refer to each pair of intervals used for training as the "latest" (T_n) and the "previous" (T_{n-1}) intervals. We then checked whether heterogeneity in the PG dynamics could enhance the prediction in these downstream networks. For each method, the RMSE was computed on the last two time intervals of another continuous sequence never seen during training, as a function of the composition of the adaptive input. We chose an input made of memory-less cells ($\beta = 0$) combined with a number $\lfloor p_m N \rfloor$ of memory cells with a memory parameter $\beta_m \geq 0$, where $p_m \in [0,1]$ is the proportion of memory cells. For each pair of (β_m, p_m) values, we trained an MLP with stochastic gradient descent and the activity of the RC with ridge regression (see Methods) to output the last two time intervals.

For both the MLP and RC, decoding the latest time interval T_n can be done regardless of heterogeneity (see Figure 10A,C). Performance is similar for most compositions of adaptive memory, although a completely memory-less network ($\beta_m = 0$ or $p_m = 0$) appears slightly better

on average for the case of the MLP with an average RMSE of 1.3 s compared to the average of 1.6 s in the heterogeneous case. This configuration was tested multiple times and can be seen in the bottom rows and left columns of all panels of Figure 10. The variability of the RMSE values comes from the random initialization of the MLP and RC and the different training and testing sequences for each point where $\beta_m = 0$ or $p_m = 0$.

Estimating the previous time interval is also impossible in the case of the MLP when the input comes from a homogeneous population, regardless of the memory parameter (see Figure 10B, bottom, left and top edges of the panel). In fact, the average error in this case is around 11.0 s when estimating the previous time interval. The error values are no better than always predicting an optimized constant value which yields an error of around 8.6 s (see section 3 of Supplementary Information). This is in accordance with the degeneracy effect that comes with a homogeneous adaptive response: different pairs of time intervals are equally likely. A heterogeneous composition of PG cells gives an average error of 7.6 s with a minimum of 5.0 s around $\beta_m = 0.5$ and $p_m = 0.7$ when estimating the previous interval. However, this minimum is wide and similar error values are found for similar values of β_m and p_m.

Interestingly, the RC estimator can still estimate the previous time interval better than chance with an average error of 7.3 s in the homogeneous case without memory, i.e. when all PG cells composing the input to the RC have $\beta = 0$ (see Figure 10D, bottom and left edges of the panel). In the homogeneous case with memory, there is an average error of 6.8 s (see Figure 10D, top edge of the panel), which indicates that having a non-zero memory parameter helps encoding the previous time interval. Similarly to the MLP, a heterogeneous PG input gives a lower average error of 5.5 s with a minimum of 4.3 s around $\beta_m = 0.6$ and $p_m = 0.8$. Again, values of β_m and p_m close to this minimum give similar RMSE values. This further confirms that heterogeneous adaptation helps encode time sequences, since it makes crucial timing information available to more sophisticated downstream decoders, especially one based on a RNN that keeps traces of past intervals.

When $\beta_m = 1$ and $p_m = 1$, the intensity of the input does not contain information about the sequence. Since $\beta_m = 1$ for all cells, there is no drop in resources during an encounter. Therefore, even if the initial amount of resources was not already full ($x = 1$), it would quickly reach that point, making the responses during each encounters share the same statistics regardless of the time elapsed between events. In that case, PG cells become non-adaptive, i.e. cells responding to any event in the same manner without depleting any resources x. This is reflected accordingly for both the MLP and the RC. Indeed, the MLP has no information about previous time intervals and therefore cannot infer the sequence; not even the latest interval. However, because the RC has recurrent dynamics and therefore some memory capacity, the non-adaptive cells still allow it to encode more than one time interval in a sequence, as can be seen in the upper right corner of Figure 10C,D. In fact, we used the performance of the RC when $\beta_m = 1$ and $p_m = 1$ to calibrate the values of some parameters of the reservoir (see Methods).

Another feature of both the MLP and the RC is the decrease in performance for a homogeneous network of PG cells as the memory parameter β_m increases when estimating the

latest time interval (see upper rows of Figure 10A,C). This is because the response intensity carries progressively less timing information as β_m increases due to the reduced dynamical range of the x resource variable.

We also tested RCs with longer time constants τ_{in} and τ_R (see Methods) related to the decay of the input signal and the decay of the activity of a unit in the RNN, respectively. In that case, the error is generally lower for both the latest and previous time intervals decoding (see Figure S4). Since the input composition and the coupling to the RNN are the same as the shorter time constant case, the only reason this estimator performs better is its increased memory capacity due to the larger values of τ_{in} and τ_R. A memory-less homogeneous input gives an average error of 6.4 s (lower and left edges of Figure S4B) while one with only memory cells gives an average error of 5.5 s (upper edge of Figure S4B). The average error in the heterogeneous case is of 4.6 s with a minimum of 3.5 s around $\beta_m = 0.9$ and $p_m = 0.7$. However, this minimum is not as sharp as in the RCs with shorter time constants and multiple values of β_m and p_m yield similar errors. Nevertheless, even with an increased memory capacity compared to the RCs with shorter time constants, heterogeneous adaptation in the input signal still significantly helps the RC in inferring sequences of multiple time intervals."

Additionally, the following is now in the DISCUSSION section at lines 2084-2135:

"We also distanced ourselves from the idealized case of the MLE by training an MLP and a RC to recover the last two time intervals (the "previous" followed by the "latest" time interval) downstream from the adaptive responses of PG cells. Both estimators were trained with different compositions of adaptive input. For both networks, as expected, the latest time interval in the sequence of encounters could be retrieved with low error regardless of heterogeneity, although a homogeneous population without memory (i.e. with $\beta_m = 0$ or $p_m = 0$) was best for the MLP estimator. We also showed how heterogeneity is necessary for estimating the previous time interval in the case of the MLP, since the error associated with an input from a homogeneous ensemble of PG cells is no better than chance.

In contrast, the RC can still recover the previous time interval even with a homogeneous PG input, although a heterogeneous PG input clearly increases the performance. We suspect that this is due to the fact that, by definition, the RNN constituting the RC keeps track to some extent of previous responses through the reverberation of activity in its recurrent connections. The increased performance in the homogeneous case compared to the MLP then comes from the stored information about the previous time intervals derived from these previous responses. This interaction between adaptive memory and recurrent memory is an interesting problem in itself; the potential synergy between these cellular and system properties should be studied further to explore how the combination of adaptive and non-adaptive circuits with varying levels of heterogeneity can best encode and decode time sequences.

Increasing the memory capacity of the RC by using larger decay rates for the input signal and the activity of the RNN not only increased the overall performance, but also made the heterogeneity of the PG input have a more subtle effect (see Figure S4). This makes sense since a RC with perfect memory could simply receive non-adaptive input (e.g. when $\beta = 1$ for all PG cells) and still encode timing information in its own recurrent activity. In that case, adaptation

of the input would simply be redundant information and would be of no use. However, this redundant information becomes useful when the memory of the RC has a limited capacity, which is the case for structures such as the pallium of the fish.

This interaction between adaptive memory and recurrent memory is an interesting problem in itself; the potential synergy between these cellular and system properties should be studied further to explore how the combination of adaptive and non-adaptive circuits with varying levels of heterogeneity can best encode and decode time sequences. “

Here is the new Figure 10 for convenience:

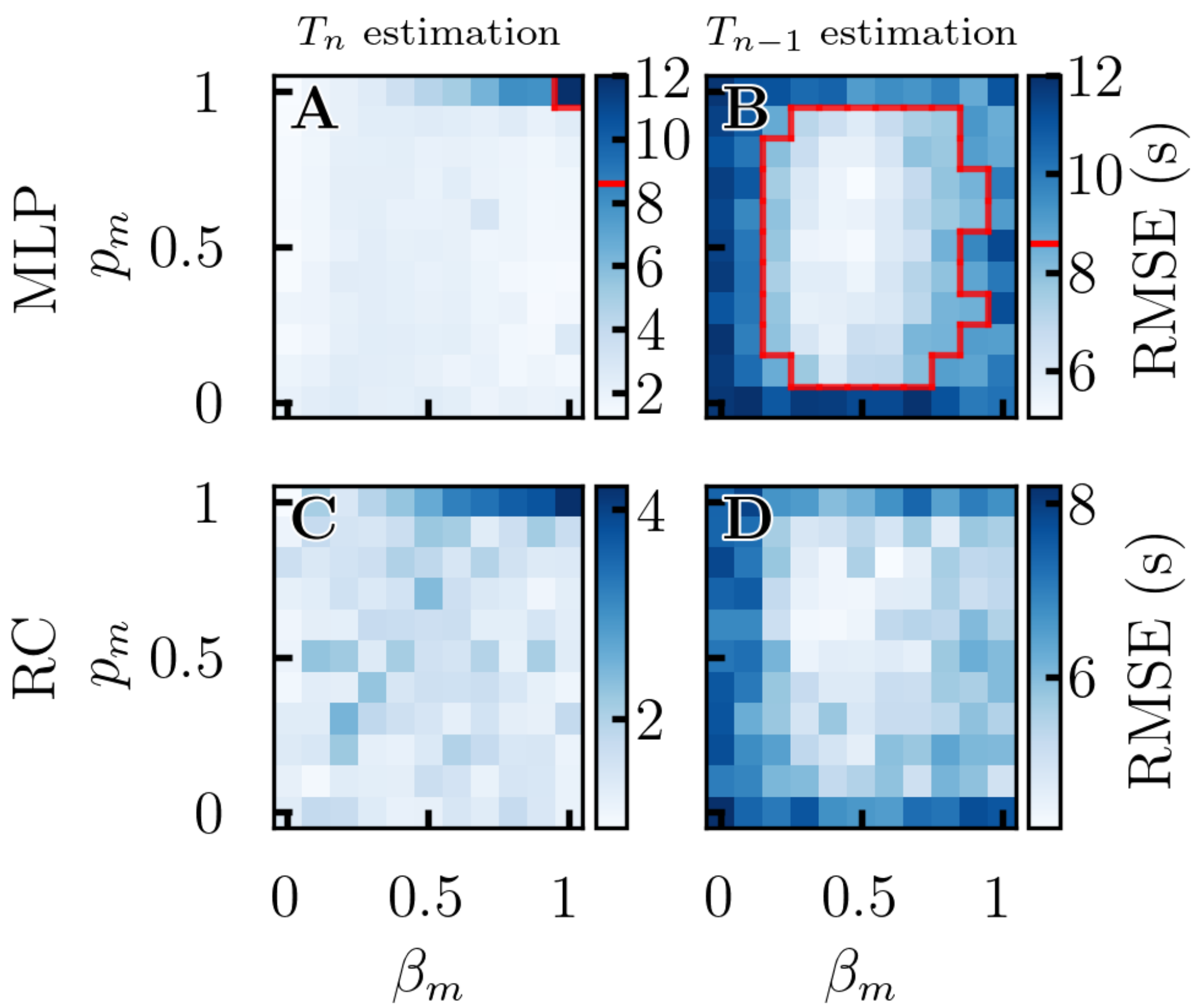

Fig. 10: Error of multilayer perceptron and reservoir computer estimators shows that heterogeneity helps with performance. Root mean square error (RMSE) on the latest time interval T_n and the previous one T_{n-1} during the presentation of random sequences of time intervals. The input to the multilayer perceptron (MLP) and reservoir computer (RC) is from $N = 1{,}000$ simulated adaptive PG neurons with varying degrees of heterogeneity with respect to the memory of their adaptation process (see Methods). A proportion $\lfloor p_m N \rfloor$ of which have a memory parameter of β_m and a time constant of $\tau = 30$ s while the other cells have no memory ($\beta = 0$) with a time constant of $\tau = 15.55$ s. All input cells have a gain $a = 10$ and baseline $c = 0$ (A) Error

of trained MLPs (for each β_m and p_m pairs) made on the estimate of the latest time interval. Over all compositions of PG cells, there is an average RMSE of 2.0 s. (B) Error of the same MLPs as in (A) made on the estimates of the previous time interval. For a homogeneous composition of PG cells (lower, left and upper edges of the panel), the average of the RMSE is of 11.0 s, which is no better than chance (see section 3 of Supplementary Information). For the heterogeneous case, the average RMSE is of 7.6 s with a minimum of 5.0 s when $\beta_m = 0.5$ and $p_m = 0.7$. (C) Error of trained RCs (for each β_m and p_m pairs) made on the estimate of latest time intervals. Over all compositions of PG cells, there is an average RMSE of 1.6 s. (D) Error of the same trained RCs as in (C) made on the estimates of the previous time interval. For a homogeneous composition of PG cells, the average of the RMSE is of 7.3 s for the memory-less case (lower and left edges of the panel) while the average of the RMSE is of 6.8 s when $\beta_m > 0$ (upper edge of the panel). When the composition is heterogeneous, the average of the RMSE is of 5.5 s with a minimum of 4.3 s when $\beta_m = 0.6$ and $p_m = 0.8$. The red lines delimit areas where the RMSE is lower (inside) than chance (< 8.6 s). Only MLP estimators have compositions of (β_m, p_m) yielding a performance worse than chance. The x and y-axes are shared by all panels.
