# Supplementary material for: Neural heterogeneity enables adaptive encoding of time sequences
Source: Commun Phys. 2025 Nov 24;8(1):504. doi: 10.1038/s42005-025-02408-3 (PMC12805876; doi:10.1038/s42005-025-02408-3)
Supplement: Supplementary file 2 [file 42005_2025_2408_MOESM2_ESM.pdf]

Supplementary Information for Neural heterogeneity enables adaptive encoding of time sequences

Supplementary Note 1. Optimal time constant for uniform and exponential distributions of time intervals. For a uniform distribution of time intervals between T_{min} and T_{max}, the moment generating function is

$$M_T(x) = \frac{e^{xT_{max}} - e^{xT_{min}}}{x(T_{max} - T_{min})} \,. \qquad (S1)$$

Taking the derivative w.r.t. τ in Equation (9) then yields

$$\frac{\partial E_T}{\partial \tau} = \frac{\tau^2}{aN}\left\{\left(\frac{3}{2} - \frac{T_{max}}{\tau}\right)e^{\frac{2T_{max}}{\tau}} - \left(\frac{3}{2} - \frac{T_{min}}{\tau}\right)e^{\frac{2T_{min}}{\tau}} - \left(3 - \frac{T_{max}}{\tau}\right)e^{\frac{T_{max}}{\tau}} + \left(3 - \frac{T_{min}}{\tau}\right)e^{\frac{T_{min}}{\tau}}\right\}. \qquad (S2)$$

We can simplify the problem by setting $T_{min} = 0$ and making the derivative zero to then solve

$$\left(\frac{3}{2} - x\right)e^{2x} - (3 - x)e^{x} + \frac{3}{2} = 0\,, \qquad (S3)$$

where $x = T_{max}/\tau$. A numerical solution can be found using Newton's root finding method. We then find that the optimal value of τ to encode this distribution of time intervals is $\tau \approx 1.14T_{max}$.
For an exponential distribution for which the expected value is given by $E_R[T] = \hat{T}$, the moment generating function is

$$M_T(x) = \frac{1}{1 - x\hat{T}} \,. \qquad (S4)$$

To get the optimal value of τ for this distribution, we need to solve

$$\frac{\tau^2}{aN}\left(\frac{\tau}{\tau - 2\hat{T}} - \frac{\tau}{\tau - \hat{T}}\right) = 0. \qquad (S5)$$

This can be simplified to solving for τ (assuming $\tau > 2\hat{T}$) in the equation

$$\tau^2 - 6\hat{T}\tau + 6\hat{T}^2 = 0\,, \qquad (S6)$$

which yields

$$\tau = \left(3 + \sqrt{3}\right)\hat{T}\,. \qquad (S7)$$

Supplementary Note 2. The optimum of the CRLB is unique for any distribution of time interval prior. Starting from the definition of the CRLB as a function of the moment generating function of the prior distribution of time intervals, an optimum in τ is found by setting the derivative to 0:

$$\frac{\partial}{\partial \tau}\left(\frac{\tau^2}{aN}\left[M_T\left(\frac{2}{\tau}\right) - M_T\left(\frac{1}{\tau}\right)\right]\right) = 0, \qquad (S8)$$

$$\Leftrightarrow 2\tau\left[M_T\left(\frac{2}{\tau}\right) - M_T\left(\frac{1}{\tau}\right)\right] + \tau^2\left[M'_T\left(\frac{2}{\tau}\right)\left(\frac{-2}{\tau^2}\right) - M'_T\left(\frac{1}{\tau}\right)\left(\frac{-1}{\tau^2}\right)\right] = 0, \qquad (S9)$$

$$\Leftrightarrow 2\tau = \frac{\left[2M'_T\left(\frac{2}{\tau}\right) - M'_T\left(\frac{1}{\tau}\right)\right]}{\left[M_T\left(\frac{2}{\tau}\right) - M_T\left(\frac{1}{\tau}\right)\right]} = g(\tau), \qquad (S10)$$

where the prime superscripts indicate a derivative w.r.t. τ. This then becomes a root-finding problem unique to each prior distribution of time intervals. However, we can leverage some properties of the moment generating function to comment on the number of roots of $2\tau = g(\tau)$. First, note that

$$\frac{\partial g}{\partial \tau} = -\frac{\left(\left[4M''_T\left(\frac{2}{\tau}\right) - M''_T\left(\frac{1}{\tau}\right)\right]\left[M_T\left(\frac{2}{\tau}\right) - M_T\left(\frac{1}{\tau}\right)\right] + \left[2M'_T\left(\frac{2}{\tau}\right) - M'_T\left(\frac{1}{\tau}\right)\right]^2\right)}{\tau^2\left[M_T\left(\frac{2}{\tau}\right) - M_T\left(\frac{1}{\tau}\right)\right]^2}. \qquad (S11)$$

Then, recall that

$$M_T(x) = \sum_{k=0}^{\infty} \frac{x^k}{k!} E_T[T^k] \qquad (S12)$$

and note that

$$M''_T(x) = \frac{\partial^2 x}{\partial \tau^2} E_T[T] + \sum_{k=2}^{\infty}\left[\frac{x^{k-2}}{(k-2)!}\left(\frac{\partial x}{\partial \tau}\right)^2 + \frac{x^{k-1}}{(k-1)!}\frac{\partial^2 x}{\partial \tau^2}\right] E_T[T^k]. \qquad (S13)$$

Therefore, we have

$$M_T\left(\frac{2}{\tau}\right) - M_T\left(\frac{1}{\tau}\right) = \sum_{k=0}^{\infty} \frac{2^k - 1}{\tau^k k!} E_T[T^k] \geq 0 \qquad (S14)$$

for distributions for which all moments are positive. This is the case for priors with positive values of T only. Moreover, we have

$$4M''_T\left(\frac{2}{\tau}\right) - M''_T\left(\frac{1}{\tau}\right) = \frac{14}{\tau^3} E_T[T] + \sum_{k=2}^{\infty} \frac{(2^{k+2} - 1)(k+1)}{\tau^{k+2}(k-1)!} E_T[T^k] \geq 0. \qquad (S15)$$

Therefore,

$$\left[4M''_T\left(\frac{2}{\tau}\right) - M''_T\left(\frac{1}{\tau}\right)\right]\left[M_T\left(\frac{2}{\tau}\right) - M_T\left(\frac{1}{\tau}\right)\right] \geq 0 \qquad (S16)$$

and $g(\tau)$ is monotonic decreasing because

$$\frac{\partial g}{\partial \tau} \leq 0. \qquad (S17)$$

Consequently, there can be at most one point where $2\tau = g(\tau)$ and, therefore, a unique optimal value of τ for any real prior distribution of time intervals.

Supplementary Note 3. A constant guess is optimal when no information is available. To assess chance-level performance of an estimator and compare it to the performance of the multi-layer perceptron (MLP) and reservoir computer (RC), we can try to optimize the mean square error (MSE) of a random estimator. Since the time intervals are uniformly distributed between $T_{min} = 0.1$ s and $T_{max} = 30$ s, we want to minimize

$$MSE = \int_{T_{min}}^{T_{max}} \int_{-\infty}^{\infty} (T - T')^2 P_{est}(T) P(T') dT dT', \qquad (S18)$$

where $P_{est}(T)$ is the estimator's probability distribution and $P(T)$ is the actual distribution of time interval values. Choosing a normal distribution with mean μ and variance σ^2 for $P_{est}(T)$, we get

$$MSE = (\mu^2 + \sigma^2) - \frac{\mu(T_{max}^2 - T_{min}^2)}{(T_{max} - T_{min})} + \frac{(T_{max}^3 - T_{min}^3)}{3(T_{max} - T_{min})}. \quad (S19)$$

Taking the partial derivative of this expression w.r.t. σ^2 and setting it to 0, we get that the optimal MSE is reached when $\sigma^2 = 0$. Then, taking the partial derivative w.r.t. μ and setting it to 0, the minimum of the MSE is found when

$$\mu_{opt} = \frac{(T_{max} + T_{min})}{2}. \quad (S20)$$

For our values, the optimal value of μ is 15.05 s which yields a root mean square error (RMSE) of 8.6 s. Therefore, we consider any value of RMSE in the MLP and RC tests above 8.6 s to be worse than chance.

Supplementary Table 1: Maximum determinant values with associated optimal population parameters

n	β_n	τ_n (s)	$detI_n$ (s^{-2n})	CRLBz_n (s)
1	0.00	7.84	9.63×10^{-2}	3.22×10^{0}
2	0.424	13.2	1.48×10^{-4}	3.94×10^{1}
3	0.699	23.7	7.56×10^{-9}	2.97×10^{2}
4	0.169	9.68	3.89×10^{-14}	1.50×10^{3}
5	0.856	47.1	1.33×10^{-20}	5.75×10^{3}
6	0.543	19.2	1.95×10^{-29}	4.42×10^{4}

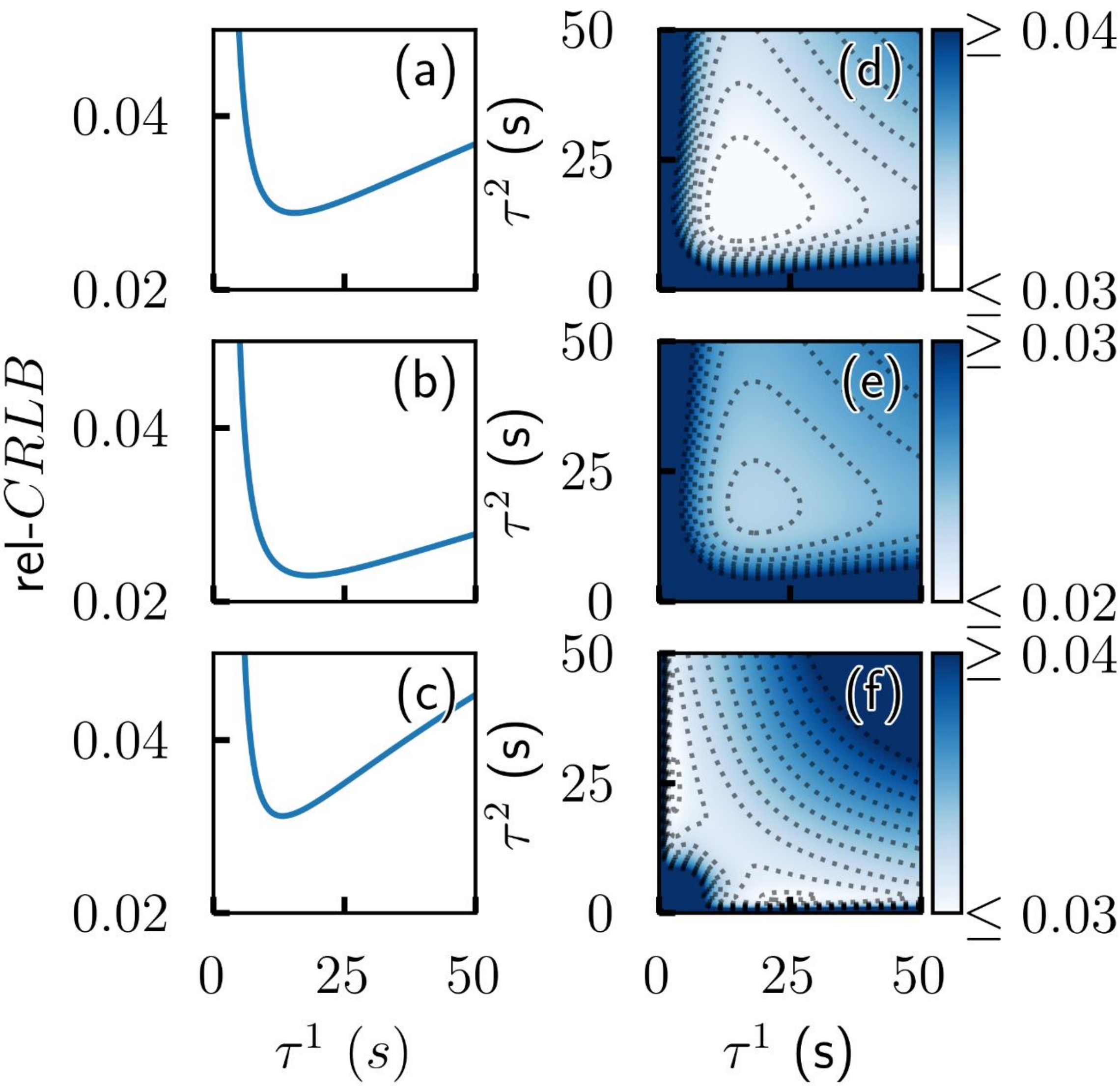

Supplementary Figure 1. Effect of heterogeneity on relative error made on estimates. (a) to (c) Single population with only one recovery parameter τ^1, gain $a = 5$, baseline activity $c = 0$ and memory parameter $\beta = 0$. (a) Relative Cramér-Rao lower bound (CRLB) as defined by Equation (13) for a time interval of $T = 10$ s. (b) Average of the relative CRLB for two time interval values of $T = 10$ s and $T = 15$ s. (c) Same as (b), but for intervals of $T = 2$ s and $T = 20$ s. (d) to (f) Two populations with two recovery parameters τ^1 and τ^2, gain parameters $a^1 = 5$ and $a^2 = 15$, baseline activity $c^1 = c^2 = 0$ and memory parameters $\beta^1 = \beta^2 = 0$. (d) Relative CRLB for a time interval of $T = 10$ s. (e) Average of the relative CRLB for two time intervals of $T = 10$ s and $T = 15$ s. (f) Average of the relative CRLB for two time intervals of $T = 2$ s and $T = 20$ s. For (d) and (e), the minimum is on the line $\tau^1 = \tau^2$ with the same value as in (a) and (b), respectively. For (f), there are two identical minima with different values of τ^1 and τ^2 due to the inclusion of a short time interval in the average relative CRLB. The minima are also different from the one in (c), which became a saddle. The x-axis is shared by all panels while the y-axis is shared column-wise.

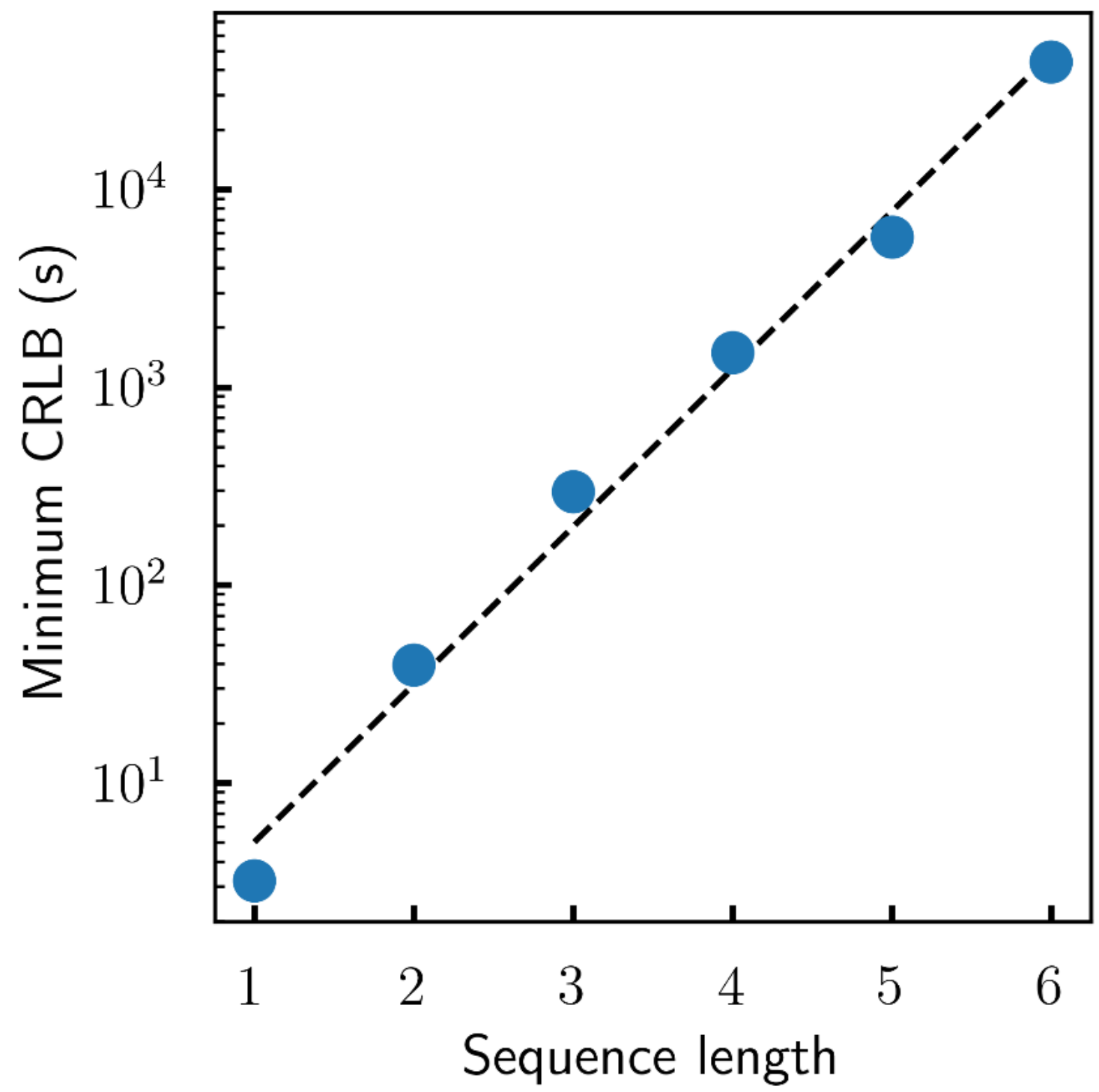

Supplementary Figure 2. The Cramér-Rao lower bound for parameters that maximize the determinant of the Fisher information matrix increases exponentially with sequence length. Cramér-Rao lower bound (CRLB) for the estimation of the sum of all time intervals in a sequence of varying length. Adding a new population with different parameters when the length of the sequence increases by one is necessary to have a finite CRLB due to the degeneracy effect. Therefore, we calculated the CRLB for a network of "effectively one neuron", i.e. the Fisher information (FI) matrix was divided by the number of cells (same as the length of the sequence, in this case) before calculating the CRLB using Equation (15). This was to make sure the values of the CRLB were for networks with identical amounts of cells. The error is expected to decrease as $1/\sqrt{N}$ for all sequence lengths. The values correspond to the sets of populations $(\beta_1, \beta_2, \dots, \tau_1, \tau_2, \dots)$ described by the red crosses of Figure 8 and the values in Supplementary Table 1.

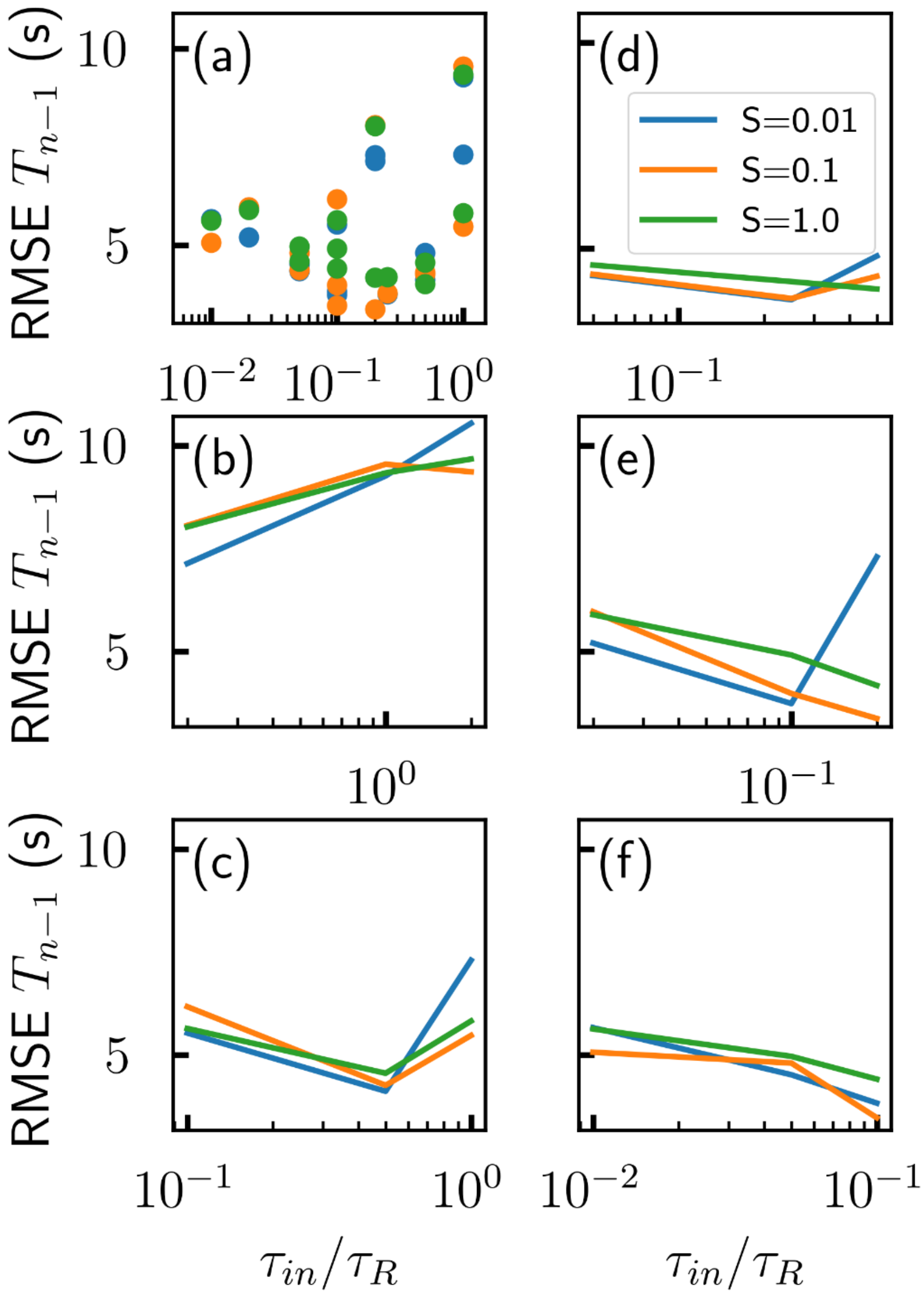

Supplementary Figure 3. Calibration of decay parameters of reservoir computer. The root mean square error of reservoir computers (RC) with different parameters for the decay of the input signal τ_{in} and the decay of the RC unit activity τ_R was computed for multiple scales of the input signal S. (a) All the RCs tested. (b) $\tau_R = 0.5$ s. (c) $\tau_R = 1.0$ s. (d) $\tau_R = 2.0$ s. (e) $\tau_R = 5.0$ s. (f) $\tau_R = 10.0$ s. A ratio of τ_{in}/τ_R between 0.1 to 0.3 appears optimal at a scale of $S = 0.1$. The lines have no significance and are there only to guide the eye. The x and y-axes are shared by all panels. The legend and colors are shared by all panels.

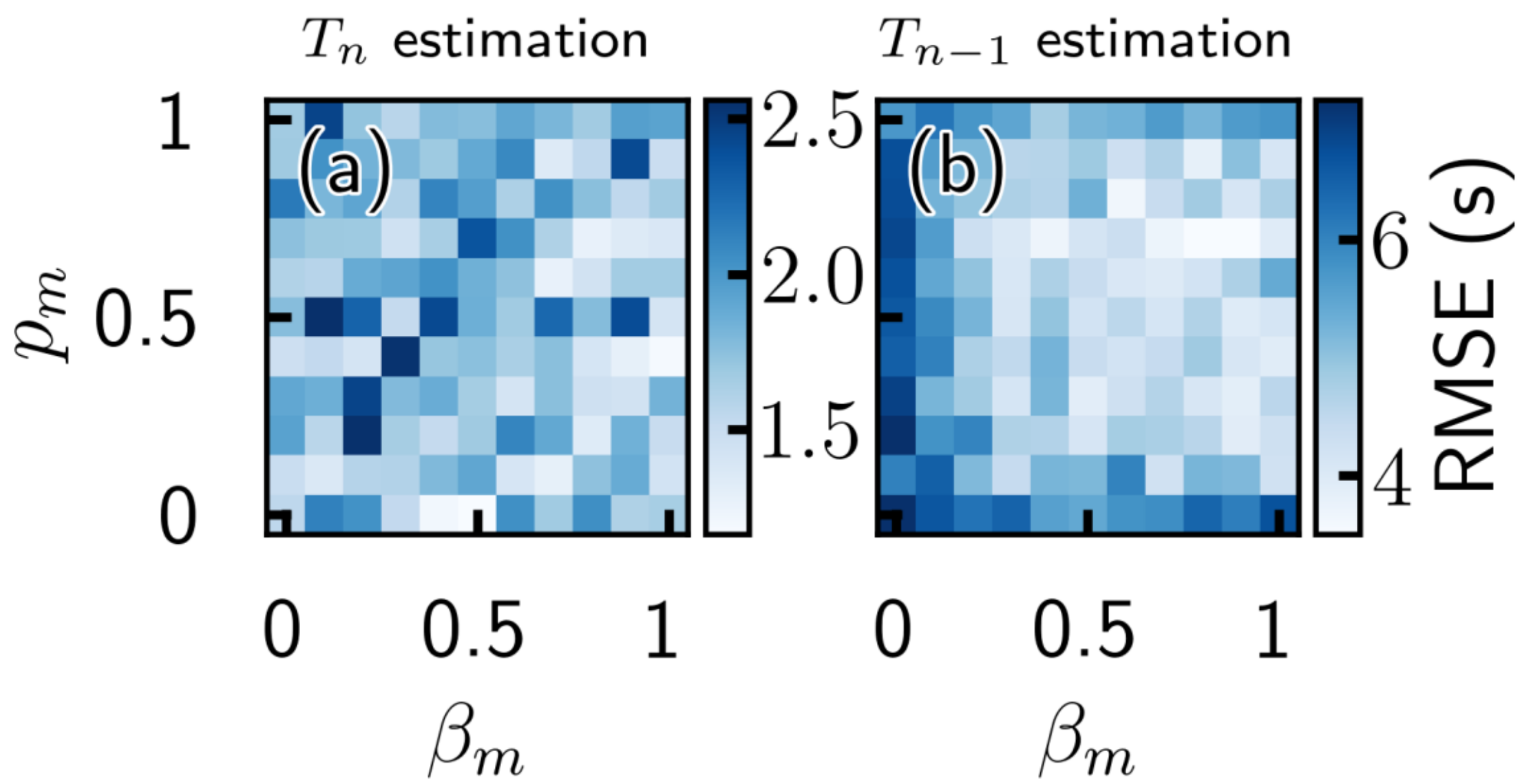

Supplementary Figure 4. Larger decay rates in the input signal and activity of a reservoir computer leads to lower error and a more subtle effect of input heterogeneity. A reservoir computer (RC) with $\tau_R = 5$ s for all units of its recurrent neural network and a decay rate of $\tau_{in} = 1\ s$ for the input layer has been trained to retrieve the last two time intervals in a long sequence of encounters. The input to the RC is from $N = 1{,}000$ simulated adaptive PG neurons with varying degrees of heterogeneity with respect to the memory of their adaptation process (see subsection "Multilayer perceptron and reservoir computer for time interval estimation" of Methods). A proportion $\lfloor p_m N \rfloor$ of which have a memory parameter of β_m and a time constant of $\tau = 30$ s while the other cells have no memory ($\beta = 0$) with a time constant of $\tau = 15.55$ s. All input cells have a gain $a = 10$ and baseline $c = 0$. For both panels, the color represents the testing RMSE in seconds. (a) The performance of the estimates of the latest time interval appears unaffected by heterogeneity and memory parameter with an average root mean square error (RMSE) of 1.7 s. (b) When estimating the previous time interval, the average RMSE for the homogeneous input is of 6.4 s for a memory-less population (lower and left edges of the panel) and of 5.5 s for a population made up 100% of memory cells (upper edge of the panel). Still, a heterogeneous input signal yields an average RMSE of 4.6 s, indicating there is still a use for heterogeneous adaptation even in the case where the RC has an increased timing information storage capacity. The x and y-axes are shared by all panels.

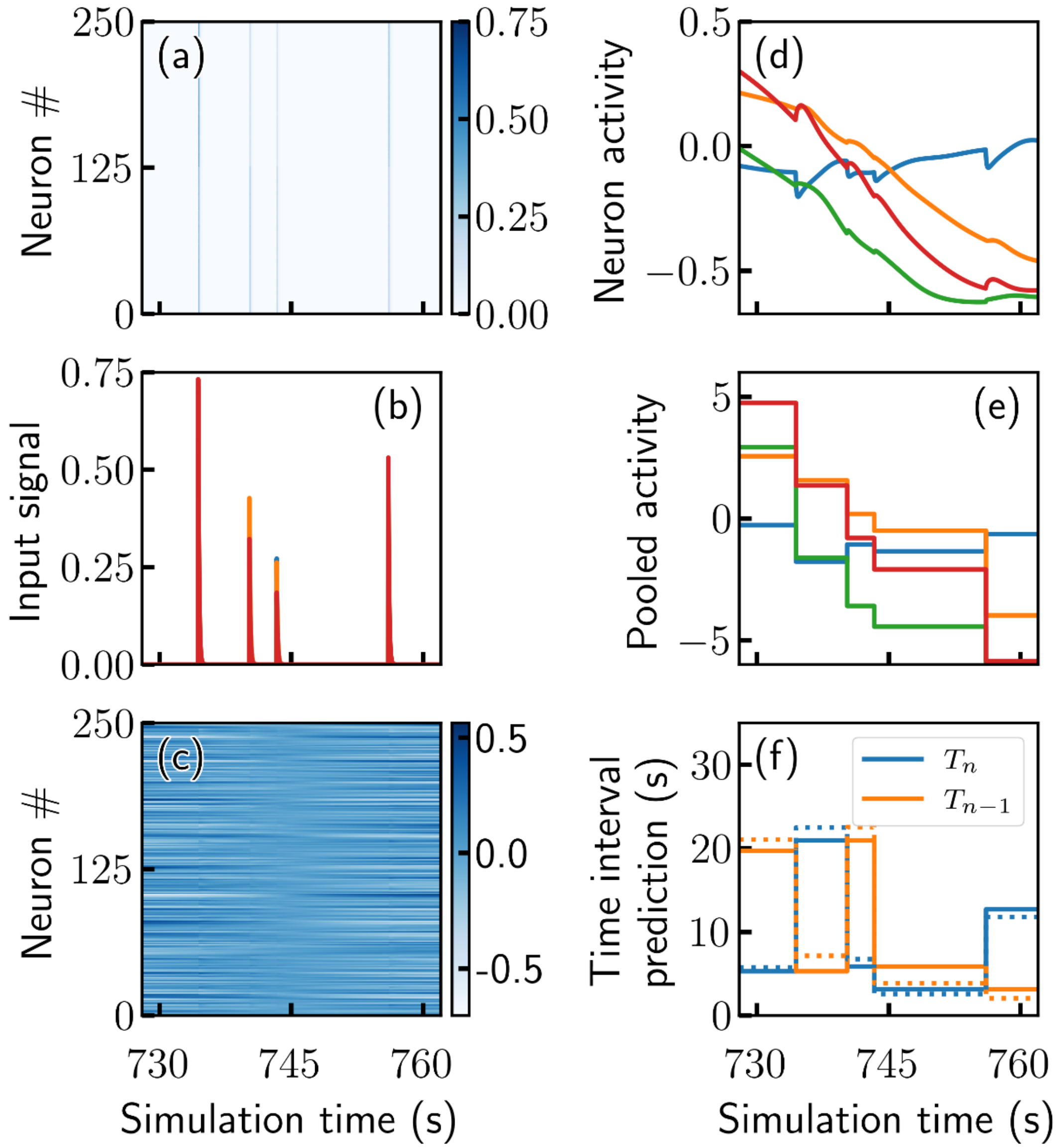

Supplementary Figure 5. Trained reservoir computer can accurately decode (i.e. infer) the latest and previous time intervals in a continuous sequence of encounters. This is data taken from the training period where a continuous sequence of 750 time intervals (i.e. 751 events) are fed to the reservoir computer (RC). Each encounters are separated by a random interval uniformly distributed between 0.1 s and 30 s. The input to the RC is from 1,000 PG cells, half of which are memory-less ($\beta = 0$) with a time constant of $\tau = 15.55$ s while the other half has memory ($\beta = 0.5$) and a time constant of $\tau = 30$ s. For both populations, the gain is $a = 10$ and the baseline is $c = 0$. For (b), (d) and (e), 4 sampled units – each corresponding to a color – were chosen from the 250 units in the RC. (a) Input $I_i(t)$ to each of the 250 neurons in the recurrent neural network (RNN). Each unit receives a random combination of the responses of the 1,000 simulated PG cells with a scaling factor of $S = 0.1$. Because the firing parameter is between 0 and 10 (maximum when $x = 1$), the input intensity should approximately be between 0 and 1. Input units to cells 1-125 come from PG memory cells (bottom), while the input to 126-250 comes from memory-less cells (top). For shorter intervals, the intensity of input coming from memory-less cells is lower as the x resource variable was completely depleted recently, whereas the input coming from memory cells is of higher intensity because the x resource variable was not depleted as much. (b) Sample of the input $I(t)$ of 4 units in the RNN. The adaptive nature of the input can be seen clearly, as a small interval

between subsequent encounters generates a weaker response. Two units (red and green) are driven by memory-less cells while the other two (blue and orange) are driven by memory cells. (c) Activated state $\tanh x_i$ of all 250 neurons in the RNN during and between encounters. The values were centered around 0 for each cell by subtracting the mean of the activity during the simulation time window that is shown. (d) Sample of the activity of 4 units in the RNN during and between encounters. (e) The activity of each unit is pooled with an exponentially weighted sum (see subsection “Multilayer perceptron and reservoir computer for time interval estimation” of Methods) between two encounters. This pooled activity is then used to train the output weights W_{out} to estimate the latest and previous time intervals. (f) Estimates (dotted lines) of the latest (blue lines) and previous (orange lines) time intervals compared with the actual time intervals of the sequence (solid lines). The x-axis is shared by all panels.

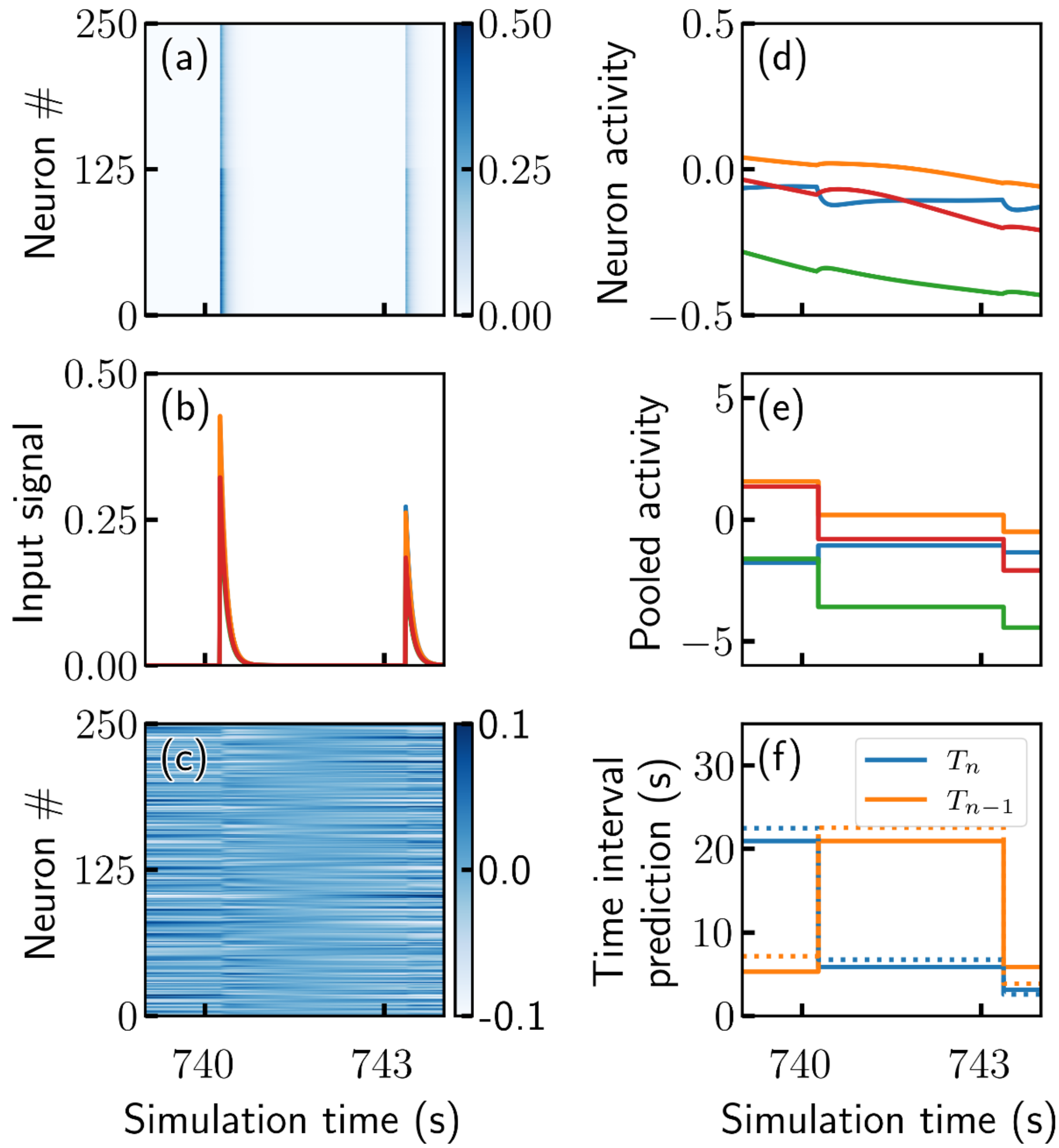

Supplementary Figure 6. Close-up view of reservoir computer activity. Same as Supplementary Figure 5, but for two encounters only. This is data taken from the training period where a continuous sequence of 750 time intervals (i.e. 751 events) are fed to the reservoir computer (RC). Each encounters are separated by a random interval uniformly distributed between 0.1 s and 30 s. The input to the RC is from 1,000 PG cells, half of which are memory-less ($\beta = 0$) with a time constant of $\tau = 15.55$ s while the other half has memory ($\beta = 0.5$) and a time constant of $\tau = 30$ s. For both populations, the gain is $a = 10$ and the baseline is $c = 0$. For (b), (d) and (e), 4 sampled units – each corresponding to a color – were chosen from the 250 units in the RC. (a) The inputs to the neurons in the recurrent neural network (RNN) are separated such that half of the units are driven by memory-less simulated PG cells (top) while the other half is driven by memory cells (bottom). (b) The input to each neuron decays exponentially back to zero until the next encounter. (c) Activated state $\tanh x_i$ of the neurons in the RNN. The values were centered around 0 for each cell by subtracting the mean of the activity during the simulation time window that is shown. (d) Sample of the activity of 5 neurons in the RNN. (e) The pooled activity is used to generate estimates as seen in (f); latest interval is in blue, previous one in orange. All panels share

the same x-axis representing the real time elapsed during the simulation. The x-axis is shared by all panels.

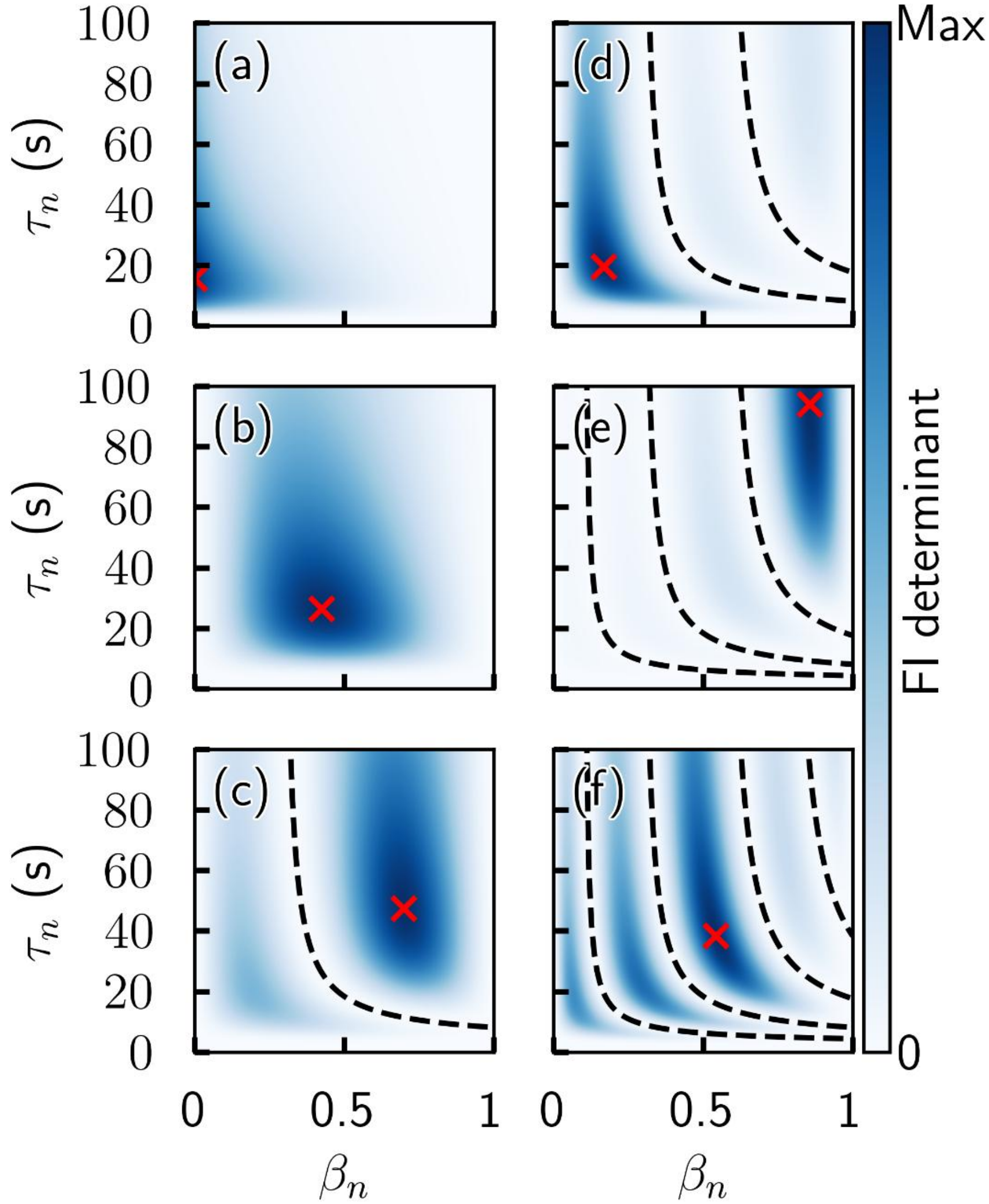

Supplementary Figure 7. A larger time interval value increases the value of τ maximizing the determinant of the FI matrix. Same as Figure 8, but with a sequence of repeated time intervals of 10 s. The number of intervals in the sequence and populations increases from 1 to 6 from panels (a) to (f). The location of the maximum (red cross) does not change much with respect to the memory parameter β, but increases significantly with τ. The locations where the determinant is zero, corresponding to the dashed lines, therefore shifts as well. The x and y-axes are shared by all panels.
